# Supplementary material for: Shape-assisted self-assembly
Source: Nat Commun. 2022 Jun 27;13:3681. doi: 10.1038/s41467-022-31482-2 (PMC9237116; doi:10.1038/s41467-022-31482-2)
Supplement: Supplementary file 1 — Supplementary Information [file 41467_2022_31482_MOESM1_ESM.pdf]

Supplementary Information for:

Shape-Assisted Self-Assembly

Joseph F. Woods[†,1], Lucía Gallego[†,1], Pauline Pfister[1], Mounir Maaloum[2], Andreas Vargas Jentzsch[†,2], Michel Rickhaus[1]*

[1]Department of Chemistry, University of Zurich; Zurich, Switzerland.
[2]SAMS Research Group, University of Strasbourg, Institut Charles Sadron, CNRS; 67200 Strasbourg, France.
[†]these authors contributed equally
*Corresponding author. Email: michel.rickhaus@chem.uzh.ch

Contents:

1. **Supplementary Figures**
2. **Experimental Procedures**
3. **NMR Characterization**
4. **Variable Temperature ^{1}H NMR Spectra**
5. **Variable Concentration ^{1}H NMR Spectra**
6. **^{1}H DOSY Spectra**
7. **Spectroscopic Data**
8. **Microscopy**
9. **Dynamic Light Scattering**
10. **Differential Scanning Calorimetry**
11. **Infrared Spectroscopy**
12. **DFT and semi-empirical calculations**
13. **X-ray Diffraction**
14. **References**

Supplementary Figures

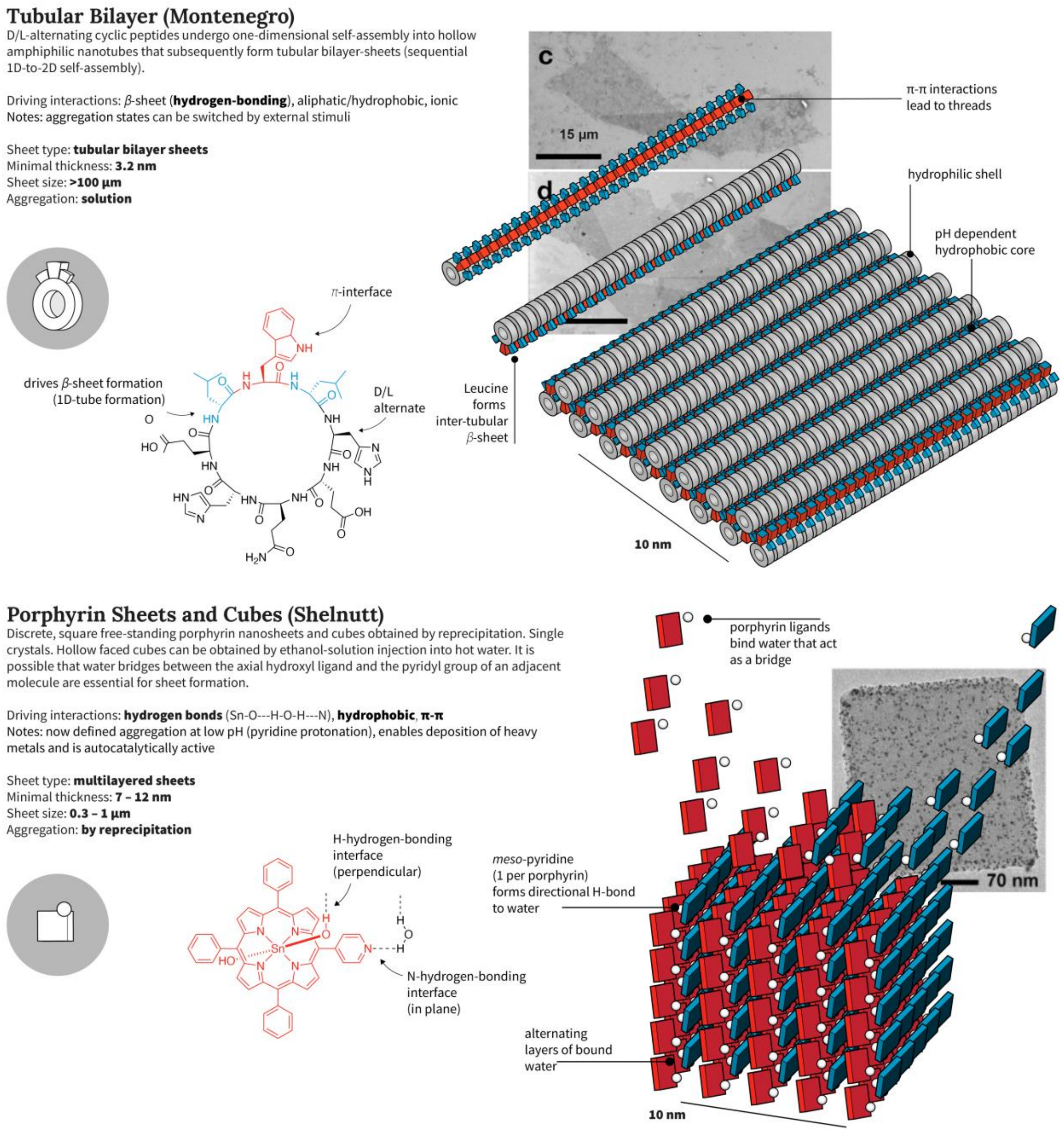

Supplementary Figure 1a. Examples of previous works on self-assembled 2D sheets from small molecules covering a range of different interactions and dimensions[S1,S2] in addition to the work reported herein.

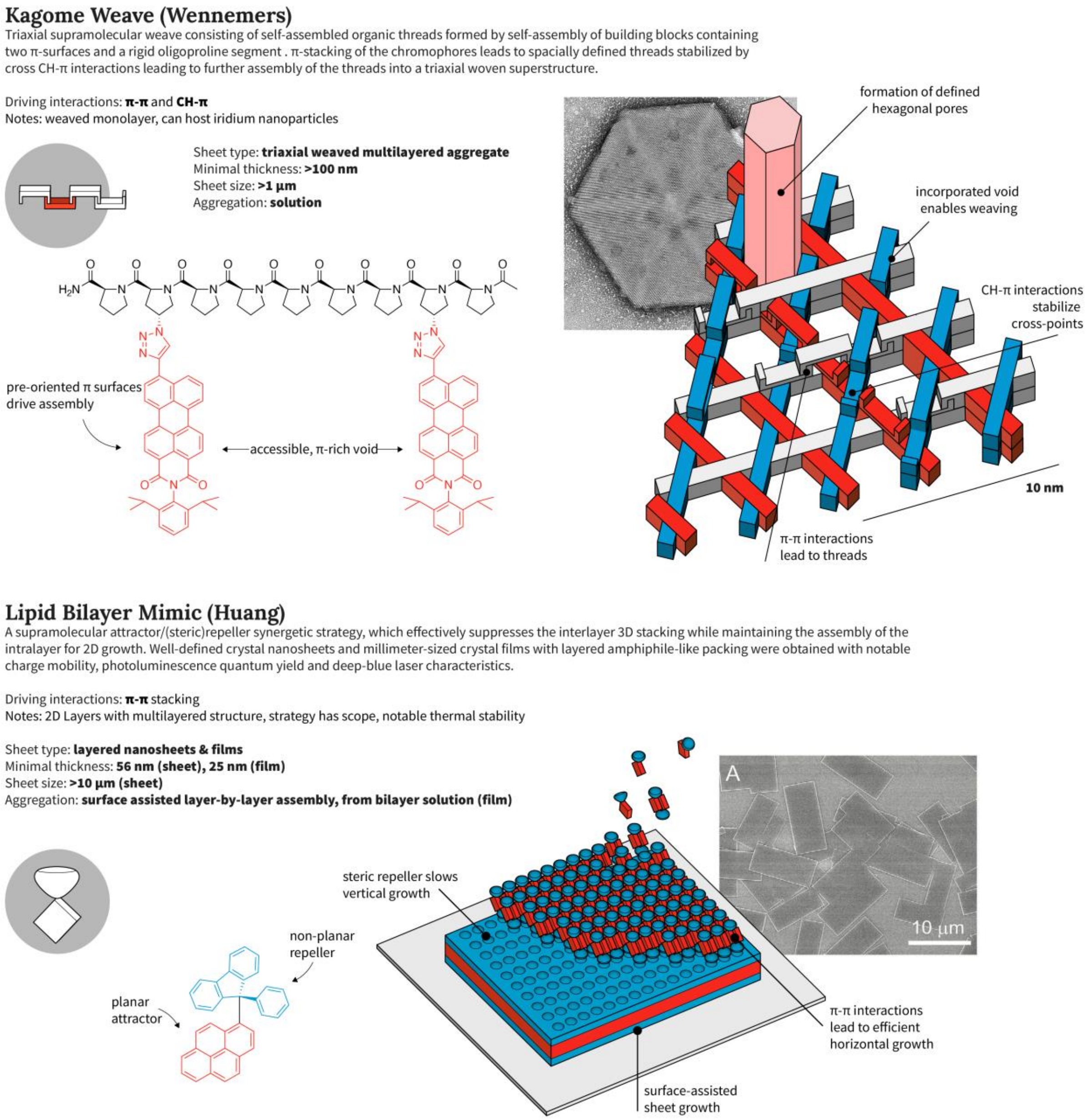

Supplementary Figure 1b. Examples of previous works on self-assembled 2D sheets from small molecules covering a range of different interactions and dimensions[S3,S4] in addition to the work reported herein.

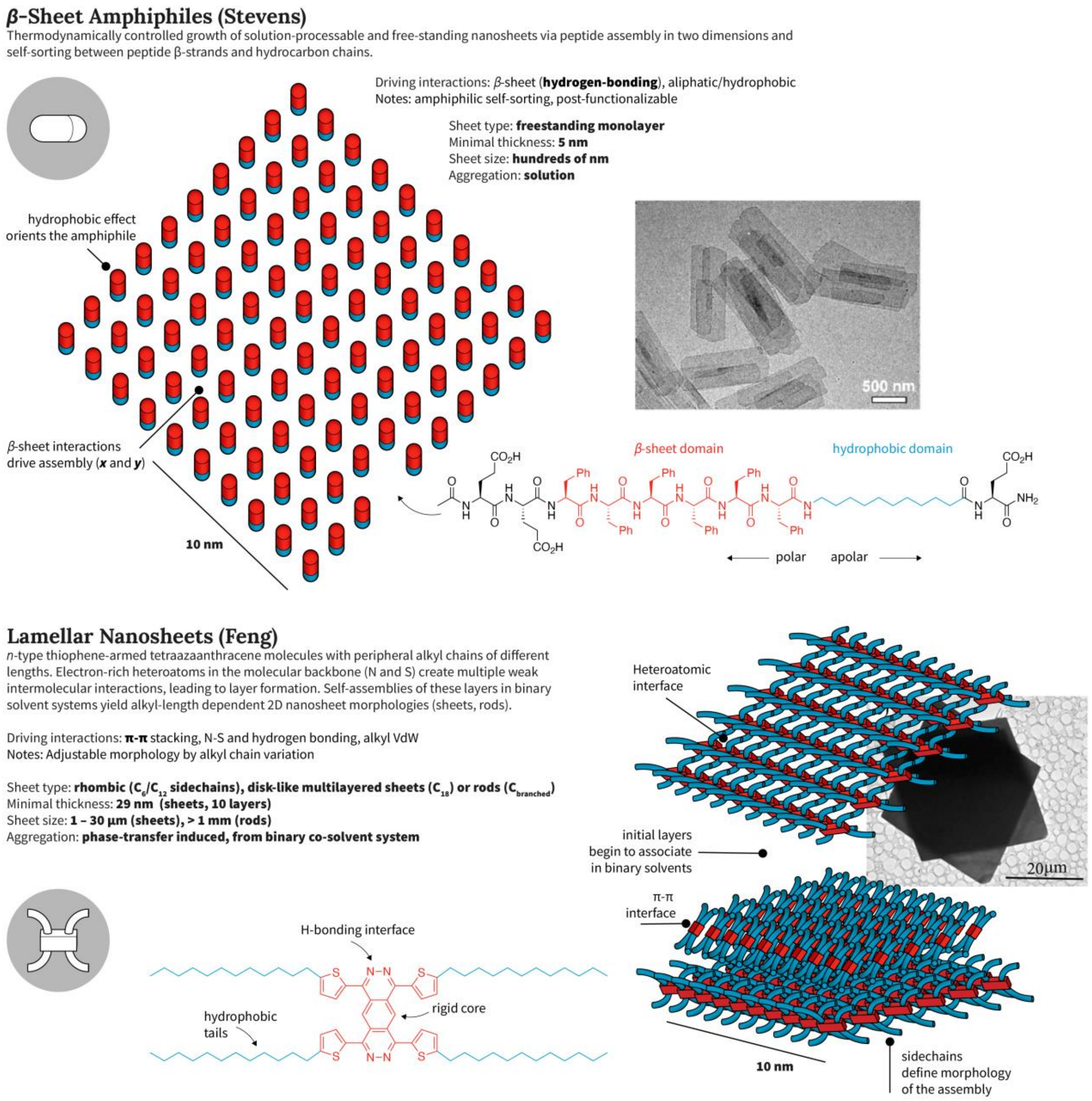

Supplementary Figure 1c. Examples of previous works on self-assembled 2D sheets from small molecules covering a range of different interactions and dimensions[S5,S6] in addition to the work reported herein.

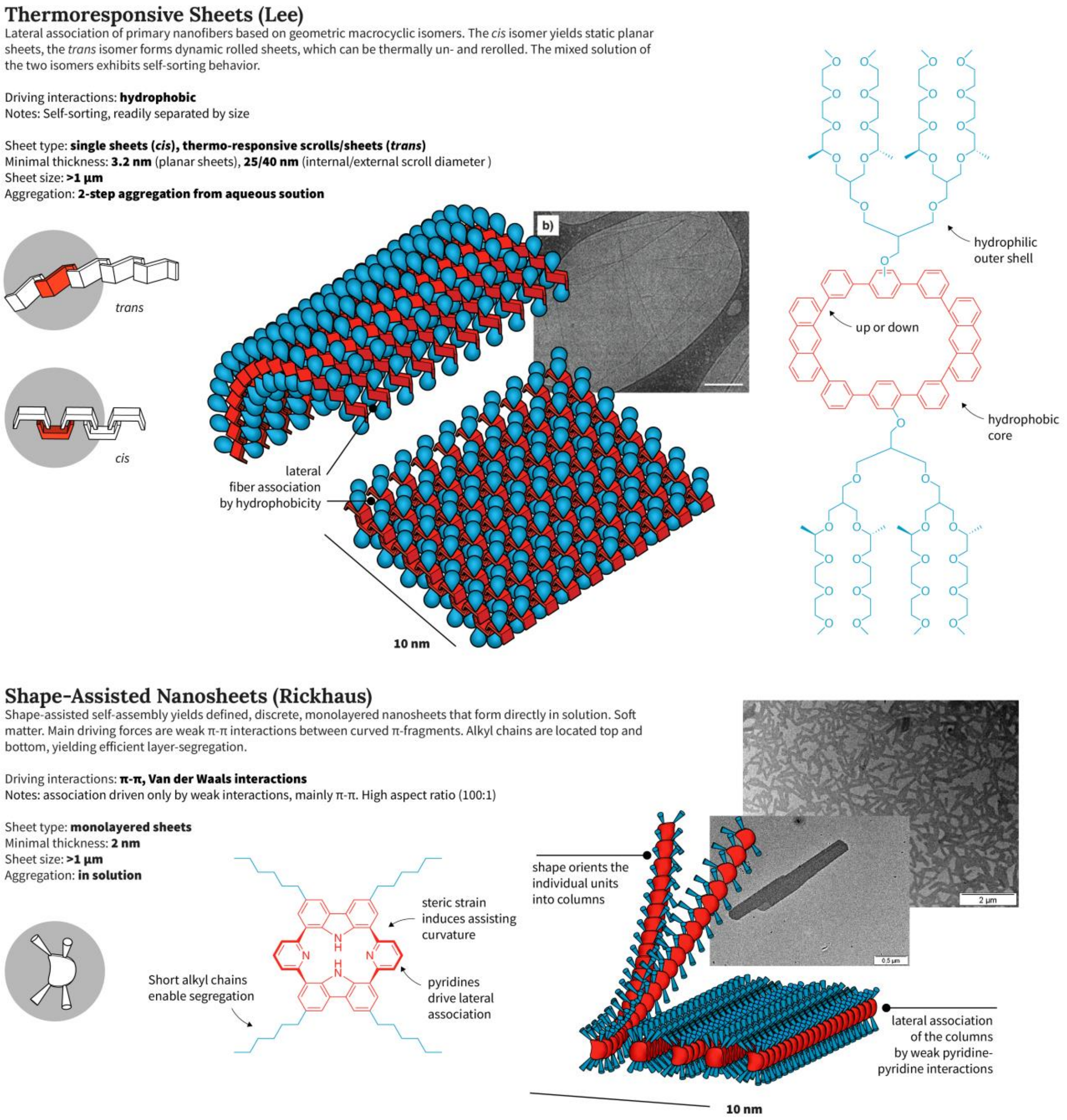

Supplementary Figure 1d. Examples of previous works on self-assembled 2D sheets from small molecules covering a range of different interactions and dimensions[S7] in addition to the work reported herein.

Experimental Procedures

Overview Schemes

a) **2H-Car-C6**, **2H-Car-C8** and **2H-Car-C12**

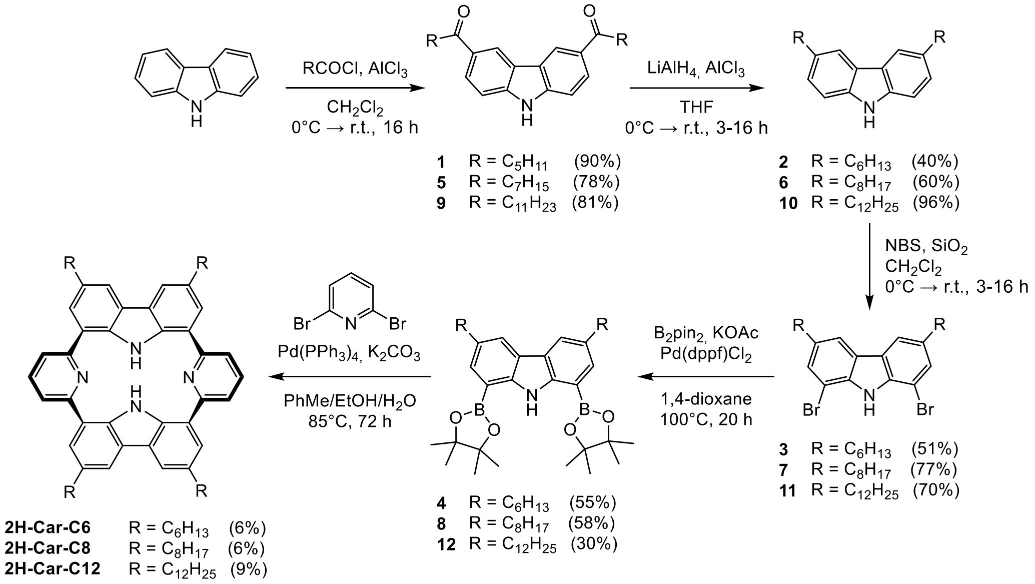

b) **2H-Car-*t*Bu** and **2H-Car-H**

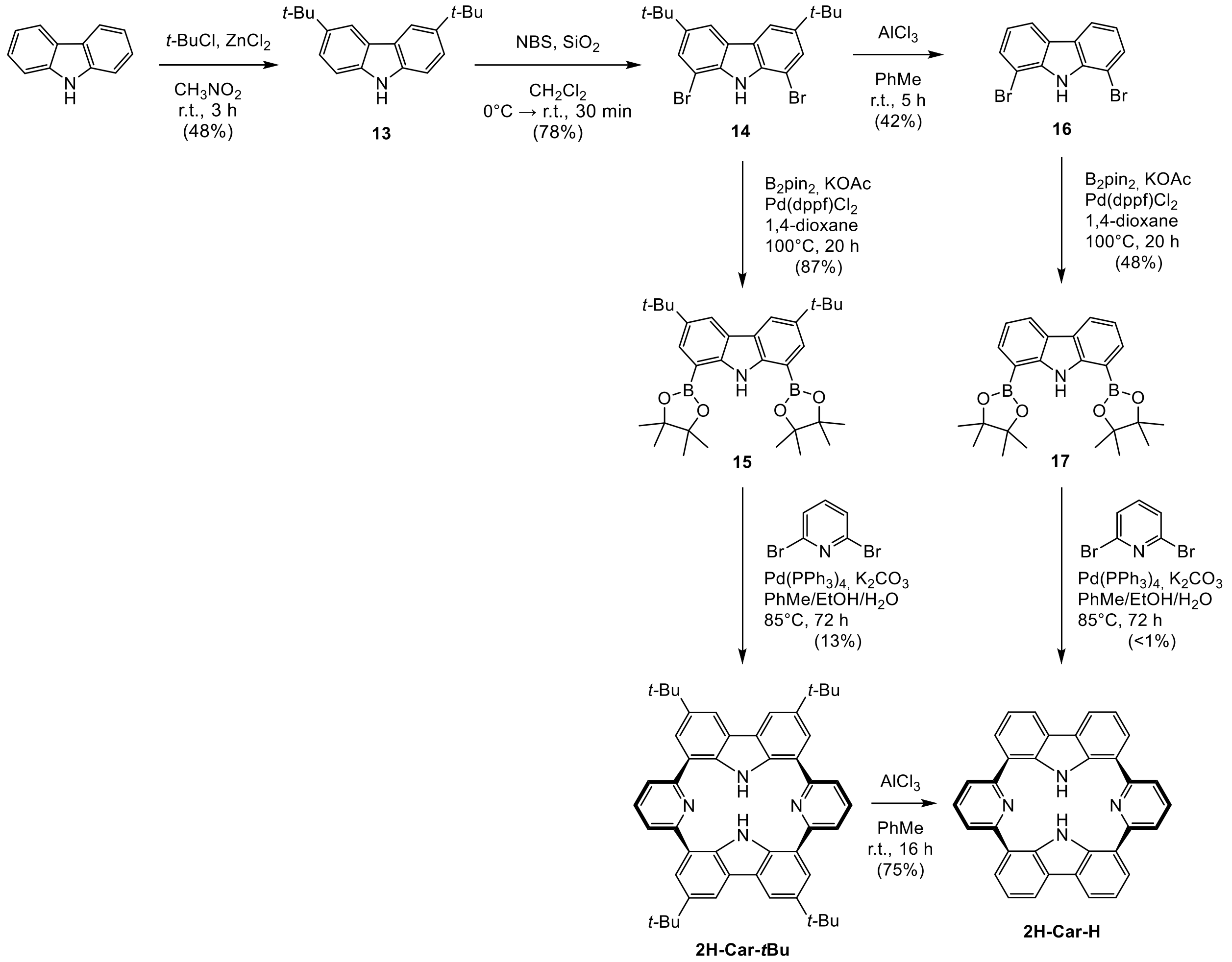

The synthesis of compounds **1**[S8], **2**[S8], **3**[S8], **9**[S9], **10**[S9], **13**[S10], **14**[S11] and **16**[S12] was performed following previously reported procedures. **2H-Car-*t*Bu** was synthesized following a modified reported procedure[S13]. Analytical data were in agreement with those reported in literature.

3,6-dihexyl-1,8-bis(4,4,5,5-tetramethyl-1,3,2-dioxaborolan-2-yl)-9*H*-carbazole (**4**)

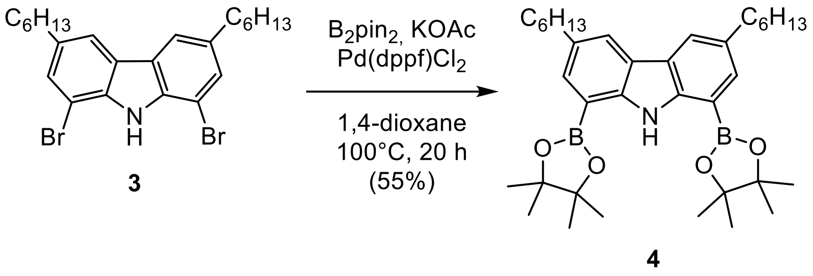

A stirring solution of **3** (2.44 g, 4.95 mmol, 1.0 eq.), B_2pin_2 (3.02 g, 11.9 mmol, 2.4 eq.) and KOAc (3.89 g, 39.6 mmol, 8.0 eq.) in 1,4-dioxane (50 mL) was degassed in a heat-gun dried Schlenk flask by bubbling N_2 through over 15 minutes. $Pd(dppf)Cl_2$ (724 mg, 0.99 mmol, 20 mol%) was added and the reaction mixture was heated to 100 °C for 20 h. The crude product was passed over a silica plug eluted with EtOAc and the solvent was removed *in vacuo*. Purification by column chromatography on silica (petroleum ether/CH_2Cl_2 6:1 to 2:1) yielded **4** (1.59 g, 55%) as an orange solid.

^{1}H NMR (400 MHz, 298 K, $CDCl_3$): δ = 10.02 (s, 1H, N-H), 7.99 (d, J = 1.7 Hz, 2H, H_{Ar}), 7.68 (d, J = 1.7 Hz, 2H, H_{Ar}), 2.79 (dd, J = 8.9, 6.7 Hz, 4H, H_{C6}), 1.77–1.69 (m, 4H, H_{C6}), 1.47 (s, 24H, H_{Bpin}), 1.38–1.31 (m, 12H, H_{C6}), 0.91 (t, J = 7.0 Hz, 6H, H_{C6}) ppm.
^{13}C NMR (101 MHz, 298 K, $CDCl_3$): δ = 144.01, 133.13, 132.79, 123.29, 122.15, 83.77, 36.21, 32.63, 31.98, 29.31, 25.30, 22.80, 14.27 ppm (the aromatic C next to the B is not observed due to the relaxation time of B).
HR-ESI-MS: m/z = 588.43992 $[M+H]^+$ ($C_{36}H_{56}O_4NB_2^+$ requires 588.43900).

2H-Car-C6

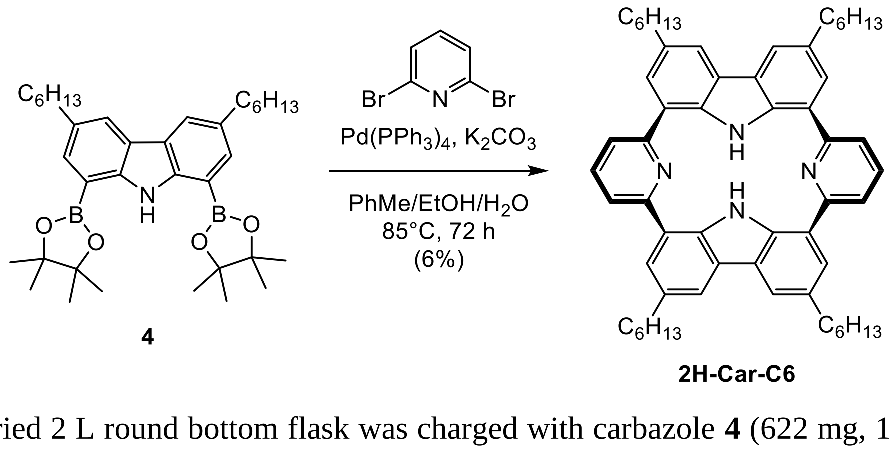

A heat gun-dried 2 L round bottom flask was charged with carbazole **4** (622 mg, 1.06 mmol, 1.0 eq.), 2,6-dibromopyridine (251 mg, 1.06 mmol, 1.0 eq.) and K_2CO_3 (1.17 g, 8.48 mmol, 8.0 eq.) in toluene (1 L), ethanol (400 mL) and water (150 mL). N_2 was bubbled through the biphasic mixture for 15 minutes and $Pd(PPh_3)_4$ (25 mg, 21 μmol, 2 mol%) was added. The flask was placed in a pre-heated oil bath and stirred at 85 °C for 72 h. The solvent was removed *in vacuo* and the crude product was dissolved in CH_2Cl_2 (100 mL) and washed with brine (3×50 mL). The organic layer was dried over Na_2SO_4 and the solvent was removed under reduced pressure. The crude mixture was purified by column chromatography on silica (petroleum ether/CH_2Cl_2 5:1), size

exclusion chromatography ($CHCl_3$) and a second silica column (petroleum ether/CH_2Cl_2 2:1). This yielded **2H-Car-C6** (38 mg, 6%) as an orange solid.

^{1}H NMR (400 MHz, 298 K, $CDCl_3$): δ = 9.67 (s, 2H, N-H), 7.98 (d, J = 1.6 Hz, 4H, H_{Ar}), 7.93 (t, J = 7.8 Hz, 2H, H_{Ar}), 7.61 (d, J = 7.8 Hz, 4H, H_{Ar}), 7.34 (d, J = 1.6 Hz, 4H, H_{Ar}), 2.85 (t, J = 7.7 Hz, 8H, H_{C6}), 1.80–1.72 (m, 8H, H_{C6}), 1.44–1.33 (m, 24H, H_{C6}), 0.92 (t, J = 7.1 Hz, 12H, H_{C6}) ppm.
^{13}C NMR (101 MHz, 298 K, $CDCl_3$): δ = 159.48, 138.29, 136.27, 134.46, 128.48, 124.80, 124.07, 122.25, 120.21, 36.16, 32.40, 31.99, 29.17, 22.80, 14.29 ppm.
HR-ESI-MS: m/z = 821.55022 $[M+H]^+$ ($C_{58}H_{69}N_4^+$ requires 821.55167).
$\lambda_{abs,max}$ (toluene) / nm (log ε): 314 (4.46), 376 (4.17).

1,1'-(9*H*-carbazole-3,6-diyl)bis(octan-1-one) (**5**)

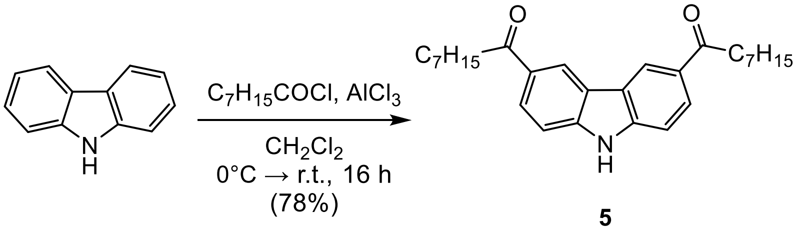

5

Octanoyl chloride (11.7 mL, 68.8 mmol, 2.3 eq.) was added to a stirring solution of $AlCl_3$ (9.17 g, 68.8 mmol, 2.3 eq.) in CH_2Cl_2 (150 mL) at 0 °C before adding carbazole (5.00 g, 29.9 mol, 1.0 eq.) in portions. The reaction mixture was warmed to room temperature and stirred for 16 h before being poured onto ice. The solid was filtered off and washed with water and MeOH which yielded compound **5** (9.75 g, 78%) as a white solid.

^{1}H NMR (400 MHz, 298 K, $CDCl_3$): δ = 8.79 (d, J = 1.1 Hz, 2H, H_{Ar}), 8.62 (s, 1H, N-H), 8.15 (dd, J_1 = 8.5 Hz, J_2 = 1.1 Hz, 2H, H_{Ar}), 7.50 (d, J = 8.7 Hz, 2H, H_{Ar}), 3.11 (t, J = 7.3 Hz, 4H, H_{C8}), 1.82 (p, J = 7.4 Hz, 4H, H_{C8}), 1.48–1.29 (m, 20H, H_{C8}), 0.90 (t, J = 6.8 Hz, 6H, H_{C8}) ppm.
^{13}C NMR (101 MHz, 298 K, $CDCl_3$): δ = 200.30, 142.99, 130.25, 127.20, 123.49, 121.80, 110.98, 38.81, 31.91, 29.62, 29.36, 24.99, 22.80, 14.25 ppm.
HR-ESI-MS: m/z = 420.28968 $[M+H]^+$ ($C_{28}H_{38}O_2N^+$ requires 420.28971).

3,6-dioctyl-9*H*-carbazole (**6**)

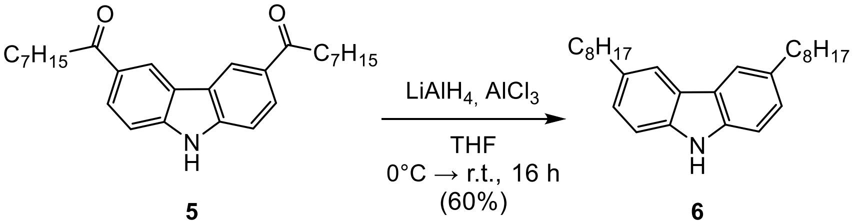

5 **6**

Lithium aluminium hydride solution (28.6 mL, 28.6 mmol, 1 M in THF, 4.0 eq.) was slowly added to $AlCl_3$ (1.91 g, 14.3 mmol, 2.0 eq.) stirring in THF (150 mL) at 0 °C under N_2. In portions, **5** (3.00 g, 7.15 mmol, 1.0 eq.) was added before the reaction mixture was warmed to room temperature and allowed to stir for 16 h. The reaction was quenched at 0 °C by adding 5% aqueous

HCl (10 mL) followed by EtOAc (30 mL) and then passed over a celite plug to remove solids, eluting with EtOAc. The mixture was concentrated *in vacuo* before being extracted with CH_2Cl_2 (100 mL) and washed with brine (100 mL). The organic phase was dried over $MgSO_4$ and the solvent was removed *in vacuo* to afford **6** (1.69 g, 60 %) as an off-white solid.

^{1}H NMR (400 MHz, 298 K, $CDCl_3$): δ = 7.89 (s, 1H, N-H), 7.85 (d, J = 0.9 Hz, 2H, H_{Ar}), 7.31 (d, J = 8.2 Hz, 2H, H_{Ar}), 7.22 (dd, J_1 = 8.3 Hz, J_2 = 1.6 Hz, 2H, H_{Ar}), 2.77 (t, J = 7.6 Hz, 4H, H_{C8}), 1.70 (p, J = 7.6 Hz, 4H, H_{C8}), 1.41–1.23 (m, 20H, H_{C8}), 0.88 (t, J = 6.6 Hz, 6H, H_{C8}) ppm.
^{13}C NMR (101 MHz, 298 K, $CDCl_3$): δ = 138.38, 133.99, 126.56, 123.57, 119.69, 110.32, 36.22, 32.51, 32.07, 29.72, 29.54, 29.46, 22.84, 14.27 ppm.
HR-ESI-MS: m/z = 392.33144 $[M+H]^+$ ($C_{28}H_{42}N^+$ requires 392.33118).

1,8-dibromo-3,6-dioctyl-9*H*-carbazole (**7**)

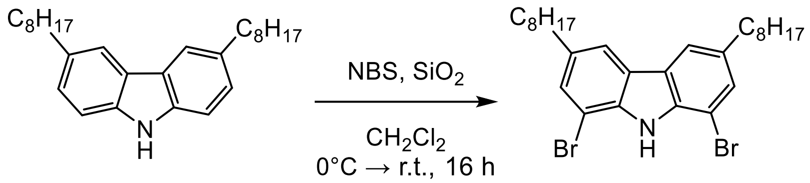

6 **7**

N-bromosuccinimide (1.61 g, 9.05 mmol, 2.1 eq.) was dissolved in CH_2Cl_2 (50 mL) and slowly added to a stirring solution of silica gel (10 g) and **6** (1.69 g, 4.31 mmol, 1.0 eq.) in CH_2Cl_2 (250 mL) at 0 °C in the dark. After addition, the reaction mixture was warmed to room temperature and stirred for 16 h. The mixture was then filtered and washed with aqueous NaOH (100 mL, 1 M) and with brine (100 mL). The organic layer was dried over $MgSO_4$ and the solvent was removed *in vacuo*. Purification *via* column chromatography on silica gel (petroleum ether/CH_2Cl_2 6:1) afforded **7** (1.82 g, 77%) as a white solid.

^{1}H NMR (400 MHz, 298 K, $CDCl_3$): δ = 8.13 (s, 1H, N-H), 7.75 (d, J = 0.8 Hz, 2H, H_{Ar}), 7.42 (d, J = 1.4 Hz, 2H, H_{Ar}), 2.74 (t, J = 7.6 Hz, 4H, H_{C8}), 1.69 (p, J = 6.9 Hz, 4H, H_{C8}), 1.38–1.24 (m, 20H, H_{C8}), 0.88 (t, J = 6.7 Hz, 6H, H_{C8}) ppm.
^{13}C NMR (101 MHz, 298 K, $CDCl_3$): δ = 136.63, 136.34, 129.29, 125.00, 119.33, 104.11, 35.92, 32.26, 32.04, 29.64, 29.41, 22.83, 14.26 ppm (1 aliphatic C is not showing due to overlapping of signals).
HR-ESI-MS: m/z = 546.13818 $[M+H]^+$ ($C_{28}H_{40}NBr_2^+$ requires 546.13765).

3,6-dioctyl-1,8-bis(4,4,5,5-tetramethyl-1,3,2-dioxaborolan-2-yl)-9*H*-carbazole (**8**)

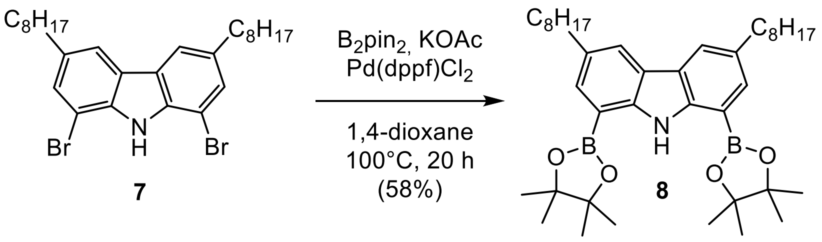

Compound **7** (1.21 g, 2.21 mmol, 1.0 eq.), B_2pin_2 (1.35 g, 5.30 mmol, 2.4 eq.) and KOAc (1.74 g, 17.7 mmol, 8.0 eq.) were dissolved in 1,4-dioxane (25 mL) and degassed in a heat-gun dried Schlenk flask by purging with N_2 for 15 minutes. $Pd(dppf)Cl_2$ (323 mg, 0.44 mmol, 20 mol%) was added and the mixture was stirred at 100 °C for 20 h. After cooling to room temperature, the mixture was passed through a celite plug and eluted with EtOAc. The solvent was removed *in vacuo* and the crude product was purified by column chromatography on silica (petroleum ether/CH_2Cl_2 2:1) to afford **8** (821 mg, 58%) as a white solid.

^{1}H NMR (400 MHz, 298 K, $CDCl_3$) δ = 10.02 (s, 1H, N-H) 7.99 (s, 2H, H_{Ar}), 7.68 (s, 2H, H_{Ar}), 2.79 (t, J = 7.8 Hz, 4H, H_{C8}), 1.73 (p, J = 7.3 Hz, 4H, H_{C8}), 1.47 (s, 24H, H_{Bpin}), 1.42–1.25 (m, 20H, H_{C8}), 0.90 (t, J = 6.6 Hz, 6H, H_{C8}) ppm.
^{13}C NMR (101 MHz, 298 K, $CDCl_3$) δ = 144.01, 133.13, 132.78, 123.29, 122.14, 83.76, 36.20, 32.67, 32.05, 29.71, 29.64, 29.46, 25.30, 22.82, 14.26 ppm (the aromatic C next to the B is not observed due to the relaxation time of B).
HR-ESI-MS: *m/z* = 644.50200 $[M+H]^+$ ($C_{40}H_{64}O_4NB_2^+$ requires 644.50160).

2H-Car-C8

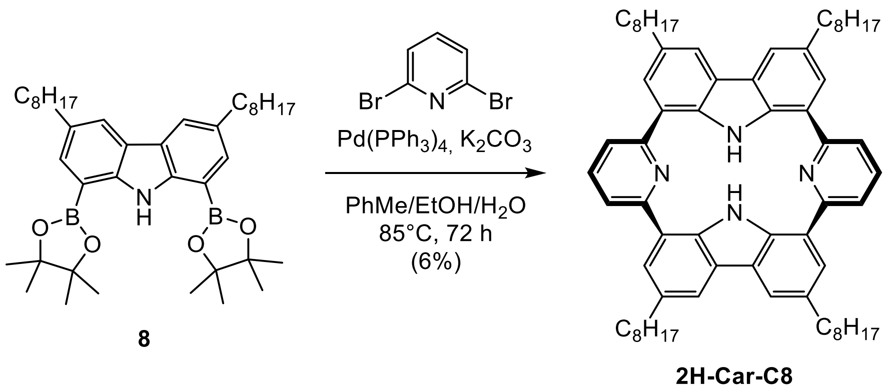

A heat gun-dried 1 L round bottom flask was charged with carbazole **8** (387 mg, 0.60 mmol, 1.0 eq.), 2,6-dibromopyridine (142 mg, 0.60 mmol, 1.0 eq.) and K_2CO_3 (665 mg, 4.81 mmol, 8.0 eq.) in toluene (500 mL), ethanol (200 mL) and water (75 mL). N_2 was bubbled through the biphasic mixture for 15 minutes and $Pd(PPh_3)_4$ (21 mg, 18 μmol, 3 mol%) was added. The flask was placed in a pre-heated oil bath and stirred at 85 °C for 72 h. The solvent was removed *in vacuo* and the crude product was dissolved in CH_2Cl_2 (100 mL) and washed with brine (3×50 mL). The organic layer was dried over Na_2SO_4 and the solvent was removed under pressure. The crude mixture was subjected to column chromatography on silica (petroleum ether/CH_2Cl_2 3:1) before performing

recycling gel permeation chromatography ($CHCl_3$). This yielded **2H-Car-C8** (31.4 mg, 6%) as a yellow solid.

^{1}H NMR (400 MHz, 298 K, $CDCl_3$) δ = 9.67 (s, 2H, N-H), 7.97 (s, 4H, H_{Ar}), 7.92 (t, J = 7.8 Hz, 2H, H_{Ar}), 7.60 (d, J = 7.8 Hz, 4H, H_{Ar}), 7.34 (s, 4H, H_{Ar}), 2.85 (t, J = 7.6 Hz, 8H, H_{C8}), 1.77 (p, J = 7.3 Hz, 8H, H_{C8}), 1.46–1.26 (m, 40H, H_{C8}), 0.90 (t, J = 6.5 Hz, 12H, H_{C8}) ppm.
^{13}C NMR (101 MHz, 298 K, $CDCl_3$) δ = 159.50, 138.27, 136.28, 134.47, 128.49, 124.81, 124.08, 122.25, 120.22, 36.16, 32.44, 32.08, 29.73, 29.50, 29.46, 22.83, 14.27 ppm.
HR-ESI-MS: m/z = 933.67593 $[M+H]^+$ ($C_{66}H_{85}N_4^+$ requires 933.67688).
$\lambda_{abs, max}$ (toluene) / nm (log ε): 314 (4.65), 376 (4.32).

1,8-dibromo-3,6-didodecyl-9*H*-carbazole (**11**)

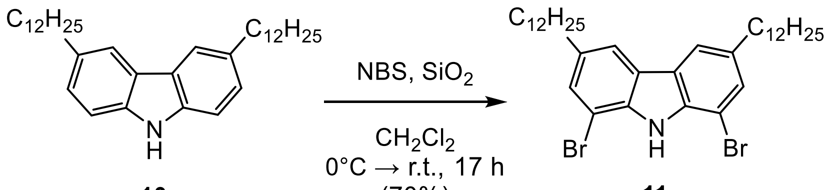

N-bromosuccinimide (2.22 g, 12.5 mmol, 2.2 eq.) was dissolved in CH_2Cl_2 (50 mL) and slowly added to a stirring solution of silica gel (11.40 g) and **10** (2.85 g, 5.66 mmol, 1.0 eq.) in CH_2Cl_2 (570 mL) at 0 °C in the dark. After addition, the reaction mixture was warmed to room temperature and stirred for 16 h. The mixture was then filtered and washed with aqueous NaOH (100 mL, 1 M) and with brine (100 mL). The organic layer was dried over Na_2SO_4 and the solvent was removed *in vacuo*. Purification *via* column chromatography on silica gel (petroleum ether/EtOAc 100:1) afforded **11** (2.61 g, 70%) as an off-white solid.

^{1}H NMR (400 MHz, 298 K, $CDCl_3$): δ = 8.13 (s, 1H, N-H), 7.74 (d, J = 1.4 Hz, 2H, H_{Ar}), 7.42 (d, J = 1.4 Hz, 2H, H_{Ar}), 2.74 (t, J = 7.9 Hz, 4H, H_{C12}), 1.72–1.65 (m, 4H, H_{C12}), 1.28–1.23 (m, 36H, H_{C12}), 0.88 (t, J = 7.0 Hz, 6H, H_{C12}) ppm.
^{13}C NMR (101 MHz, 298 K, $CDCl_3$): δ = 136.58, 136.27, 129.24, 124.96, 119.27, 104.09, 35.93, 32.25, 32.09, 29.84, 29.81, 29.76, 29.69, 29.52, 29.43, 22.86, 14.28 ppm (1 aliphatic C is not showing due to overlapping of signals).
HR-EI-MS: *m/z* = 659.26856 $M^{+\bullet}$ ($C_{36}H_{55}NBr_2^{+\bullet}$ requires 659.26958).

3,6-didodecyl-1,8-bis(4,4,5,5-tetramethyl-1,3,2-dioxaborolan-2-yl)-9*H*-carbazole (**12**)

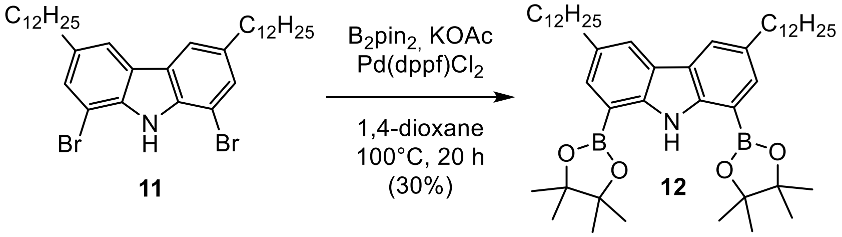

Compound **11** (2.61 g, 3.94 mmol, 1.0 eq.), B_2pin_2 (2.41 g, 9.48 mmol, 2.4 eq.) and KOAc (3.10 g, 31.6 mmol, 8.0 eq.) were dissolved in 1,4-dioxane (50 mL) and degassed in a heat-gun dried Schlenk flask by purging with N_2 for 15 minutes. $Pd(dppf)Cl_2$ (578 mg, 0.79 mmol, 20 mol%) was added and the mixture was stirred at 100 °C for 20 h. After cooling to room temperature, the mixture was passed through a celite plug and eluted with EtOAc. The solvent was removed *in vacuo* and the crude product was purified by column chromatography on silica (petroleum ether/CH_2Cl_2 6:1) to afford **12** (906 mg, 30%) as a yellow oil.

^{1}H NMR (400 MHz, 298 K, $CDCl_3$): δ = 10.04 (s, 1H, N-H), 8.00 (d, J = 1.8 Hz, 2H, H_{Ar}), 7.69 (d, J = 1.8 Hz, 2H, H_{Ar}), 2.80 (t, J = 7.8 Hz, 4H, H_{C12}), 1.78–1.70 (m, 4H, H_{C12}), 1.48 (s, 24H, H_{Bpin}), 1.33–1.26 (m, 36H, H_{C12}), 0.91 (t, J = 7.1 Hz, 6H, H_{C12}) ppm.
^{13}C NMR (101 MHz, 298 K, $CDCl_3$): δ = 144.03, 133.14, 132.79, 123.30, 122.17, 83.77, 36.24, 32.70, 32.08, 29.86, 29.81, 29.78, 29.67, 29.52, 25.31, 22.85, 14.28 ppm (the aromatic C next to the B is not observed due to the relaxation time of B and 1 aliphatic C is not showing due to overlapping of signals).
HR-ESI-MS: m/z = 756.62709 $[M+H]^+$ ($C_{48}H_{80}O_4NB_2^+$ requires 756.62680).

2H-Car-C12

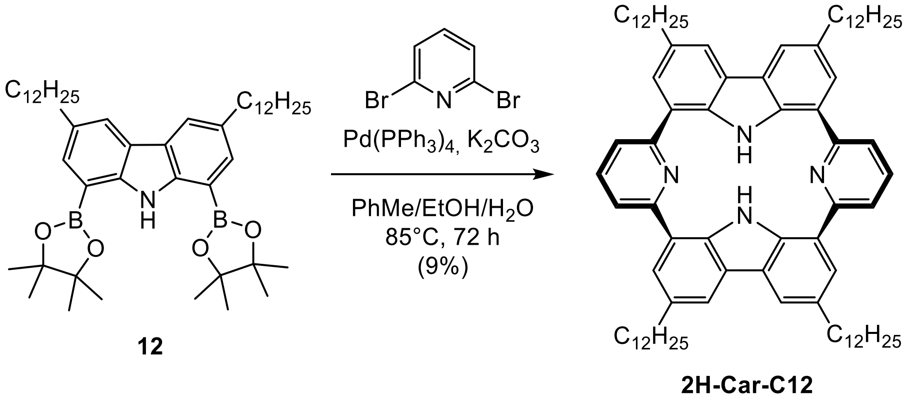

A heat gun-dried 250 mL round bottom flask was charged with carbazole **12** (55 mg, 73 µmol, 1.0 eq.), 2,6-dibromopyridine (17 mg, 73 µmol, 1.0 eq.) and K_2CO_3 (80.5 mg, 0.58 mmol, 8.0 eq.) in toluene (125 mL), ethanol (50 mL) and water (18 mL). N_2 was bubbled through the biphasic mixture for 15 minutes and $Pd(PPh_3)_4$ (1.7 mg, 1.5 µmol, 2 mol%) was added. The flask was placed in a pre-heated oil bath and stirred at 85 °C for 72 h. The solvent was removed *in vacuo* and the crude product was dissolved in CH_2Cl_2 (100 mL) and washed with brine (3×50 mL). The organic layer was dried over Na_2SO_4 and the solvent was removed under pressure. The crude mixture was subjected to column chromatography on silica twice (petroleum ether/CH_2Cl_2 3:1, then 5:2) before

performing recycling gel permeation chromatography ($CHCl_3$). This yielded **2H-Car-C12** (3.9 mg, 9%) as an orange solid.

^{1}H NMR (400 MHz, 298 K, $CDCl_3$): δ = 9.68 (s, 2H, N-H), 7.96 (d, J = 1.4 Hz, 4H, H_{Ar}), 7.96 (t, J = 7.8 Hz, 2H, H_{Ar}), 7.64 (d, J = 7.8 Hz, 4H, H_{Ar}), 7.34 (d, J = 1.4 Hz, 4H, H_{Ar}), 2.82 (t, J = 7.7 Hz, 8H, H_{C12}), 1.75–1.68 (m, 8H, H_{C12}), 1.29–1.20 (m, 72H, H_{C12}), 0.86 (t, J = 7.0 Hz, 12H, H_{C12}) ppm.
^{13}C NMR (101 MHz, 298 K, $CDCl_3$): δ = 159.51, 138.28, 136.33, 134.48, 128.45, 124.81, 124.14, 122.26, 120.23, 36.18, 32.47, 32.07, 29.86, 29.84, 29.80, 29.77, 29.51, 22.84, 14.27 ppm (2 aliphatic C are not showing due to overlapping of signals).
HR-ESI-MS: m/z = 1157.92915 $[M+H]^+$ ($C_{82}H_{117}N_4^+$ requires 1157.92728).
$\lambda_{abs,max}$ (toluene) / nm (log ε): 314 (4.92), 375 (4.55).

3,6-di-tert-butyl-1,8-bis(4,4,5,5-tetramethyl-1,3,2-dioxaborolan-2-yl)-9H-carbazole (**15**)

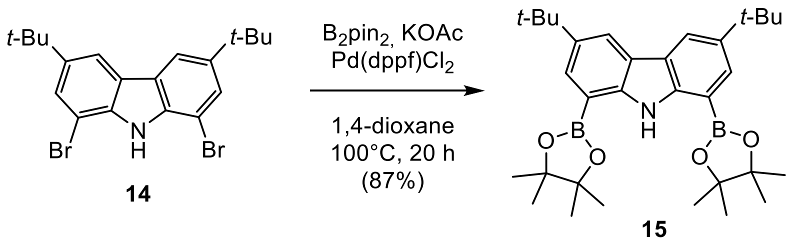

Compound **14** (4.00 g, 9.15 mmol, 1.0 eq.), B_2pin_2 (5.58 g, 22.0 mmol, 2.4 eq.) and KOAc (7.19 g, 73.2 mmol, 8.0 eq.) were dissolved in 1,4-dioxane (80 mL) and degassed in a heat-gun dried Schlenk flask by purging with N_2 for 15 minutes. $Pd(dppf)Cl_2$ (1.34 g, 1.83 mmol, 20 mol%) was added and the mixture was stirred at 100 °C for 20 h. After cooling to room temperature, the mixture was passed through a silica plug and eluted with EtOAc. The solvent was removed *in vacuo* and the crude product was subjected to column chromatography on silica (petroleum ether/CH_2Cl_2 6:1) to yield **15** (4.22 g, 87%) as a white solid.

Analytical data were in agreement with those reported in literature[S13].

2H-Car-*t*Bu

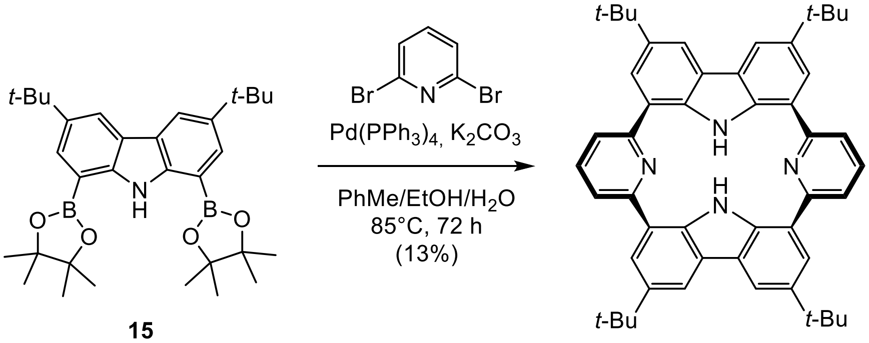

15

2H-Car-*t*Bu

Carbazole **15** (250 mg, 0.47 mmol, 1.0 eq.), 2,6- dibromopyridine (114 mg, 0.47 mmol, 1.0 eq.) and K_2CO_3 (520 mg, 3.76 mmol, 8.0 eq.) were dissolved in toluene (1 L), ethanol (400 mL) and water (150 mL) in a heat-gun dried 2 L round bottom flask. The mixture was degassed by purging with N_2 for 30 minutes and $Pd(PPh_3)_4$ (27.2 mg, 24 µmol, 5 mol%) was added. The reaction mixture was heated to 85 °C in a pre-heated oil bath for 72 h. After cooling to room temperature, the solvent was removed *in vacuo.* The crude was dissolved in CH_2Cl_2 (100 mL) and the organic phase was washed with brine (3×50 mL). The organic layer was dried over Na_2SO_4 and the solvent was removed under reduced pressure. The crude product was subjected to column chromatography on silica (petroleum ether/EtOAc 100:1) and then recrystallized from MeOH/CH_2Cl_2 1:1 to yield **2H-Car-*t*Bu** (22 mg, 13%) as a light brown solid.

Analytical data were in agreement with those reported in literature[S13].

1,8-bis(4,4,5,5-tetramethyl-1,3,2-dioxaborolan-2-yl)-9H-carbazole (**18**)

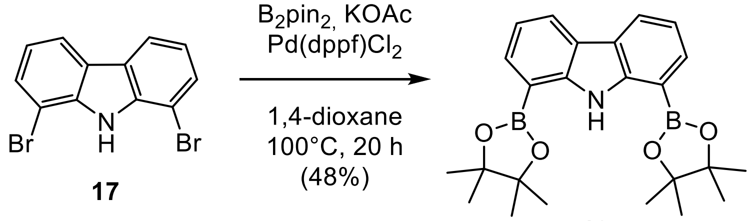

17

18

Carbazole **17** (379 mg, 1.17 mmol, 1.0 eq.), B_2pin_2 (713 mg, 2.81 mmol, 2.4 eq.) and KOAc (919 mg, 9.36 mmol, 8.0 eq.) were dissolved in 1,4-dioxane (15 mL) and degassed in a heat-gun dried Schlenk flask by purging with N_2 for 15 minutes. Pd(dppf)Cl_2 (171 mg, 0.23 mmol, 20 mol%) was added and the mixture was stirred at 100 °C for 20 h. After cooling to room temperature, the mixture was passed through a silica plug eluted with EtOAc and the solvent was removed *in vacuo*. Recrystallization from cyclohexane yielded **18** (48%) as a white solid.

Analytical data were in agreement with those reported in literature[S14].

2H-Car-H

Two different synthetic methods are herein reported for the synthesis of **2H-Car-H**, the first one being a macrocyclization from carbazole **18**, analogous to the rest of derivatives, and the second one based on a reverse Friedel-Crafts alkylation reaction on **2H-Car-*t*Bu**.

Procedure 1

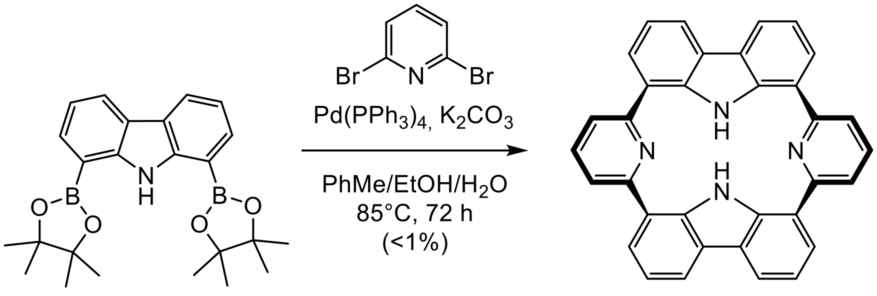

18 **2H-Car-H**

A heat gun-dried 500 mL round bottom flask was charged with carbazole **18** (118 mg, 0.28 mmol, 1.0 eq.), 2,6-dibromopyridine (67 mg, 0.28 mmol, 1.0 eq.) and K_2CO_3 (312 mg, 2.26 mmol, 8.0 eq.) in toluene (250 mL), ethanol (100 mL) and water (37.5 mL). N_2 was bubbled through the biphasic mixture for 15 minutes and $Pd(PPh_3)_4$ (6.5 mg, 6 μmol, 2 mol%) was added. The flask was placed in a pre-heated oil bath and stirred at 85 °C for 72 h before the solvent was removed *in vacuo*. Twenty batches of this scale were combined, dissolved in CH_2Cl_2 (100 mL) and washed with brine (3×50 mL). The organic layer was dried over Na_2SO_4 and the solvent was removed under reduced pressure. The mixture was purified by column chromatography on silica (CH_2Cl_2/EtOAc 3:1) and size exclusion chromatography ($CHCl_3$). Precipitation from a CH_2Cl_2/MeOH 1:1 mixture yielded **2H-Car-H** as a white solid (1.6 mg, 0.1%).

^{1}H NMR (600 MHz, 298 K, DMSO-d$_6$): δ = 11.53 (s, 2H, N-H), 8.29 (d, J = 7.7 Hz, 4H, H_{Ar}), 8.13 (t, J = 7.8 Hz, 2H, H_{Ar}), 7.71 (d, J = 7.8 Hz, 4H, H_{Ar}), 7.51 (d, J = 7.4 Hz, 4H, H_{Ar}), 7.33 (t, J = 7.6 Hz, 4H, H_{Ar}) ppm.
^{13}C NMR (151 MHz, 298 K, DMSO-d$_6$): δ = 158.17, 139.05, 137.15, 127.30, 124.82, 123.29, 122.19, 120.60, 119.20 ppm.
HR-ESI-MS: *m/z* = 485.17607 [M+H]$^+$ ($C_{34}H_{21}N_4^+$ requires 485.17607).
$\lambda_{abs,max}$ (toluene) / nm (log ε): 304 (4.21), 363 (3.84).

Procedure 2

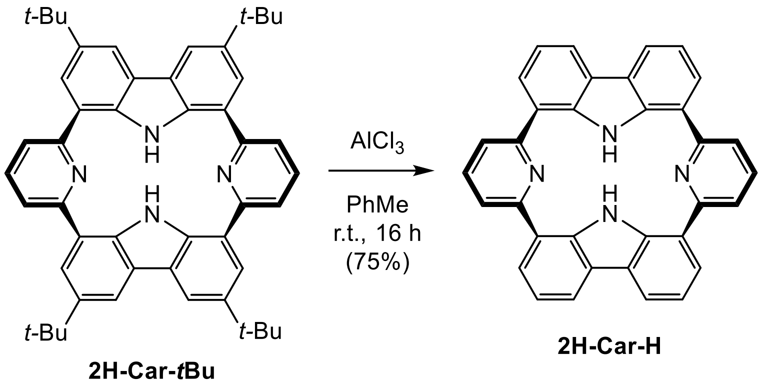

A 50 mL round bottomed flask was charged with **2H-Car-*t*Bu** (42 mg, 0.06 mmol, 1.0 e.q.) in anhydrous toluene (10 mL) and $AlCl_3$ (47 mg, 0.36 mmol, 6.0 eq.) was added. The yellow reaction mixture was stirred overnight until a brown precipitate was formed. The brown solid was filtered off from the reaction mixture and collected. The filtrate was concentrated, and the same reaction conditions were applied during a reaction time of 30 minutes. A brown precipitate was filtered off again and the filtrate was subjected to identical conditions one further time. This yielded **2H-Car-H** (21 mg, 75%) when all brown precipitates were combined.

Analytical data match that of the previous macrocyclic synthesis.

NMR Characterization

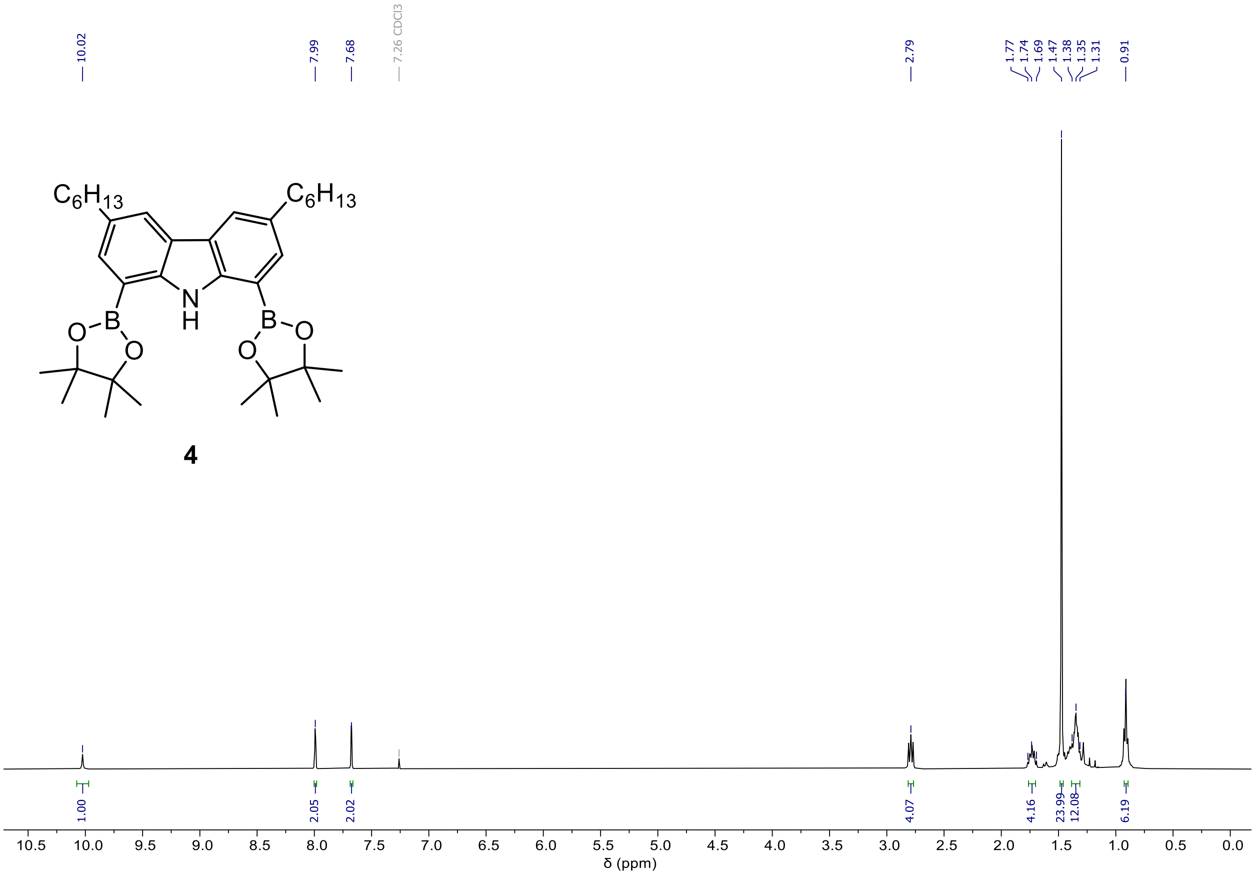

Supplementary Figure 2. ^{1}H NMR of **4** (400 MHz, 298 K, $CDCl_3$).

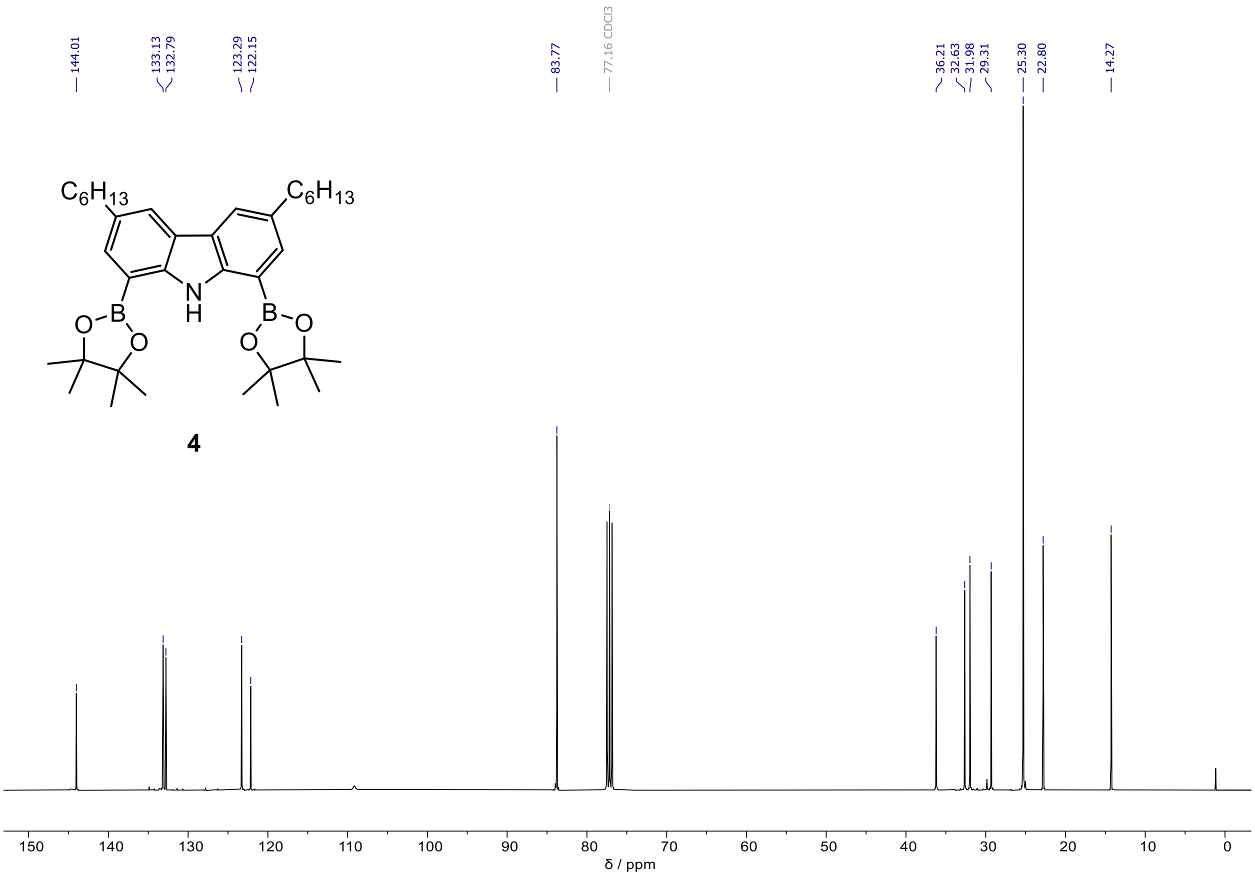

Supplementary Figure 3. ^{13}C NMR of **4** (101 MHz, 298 K, $CDCl_3$).

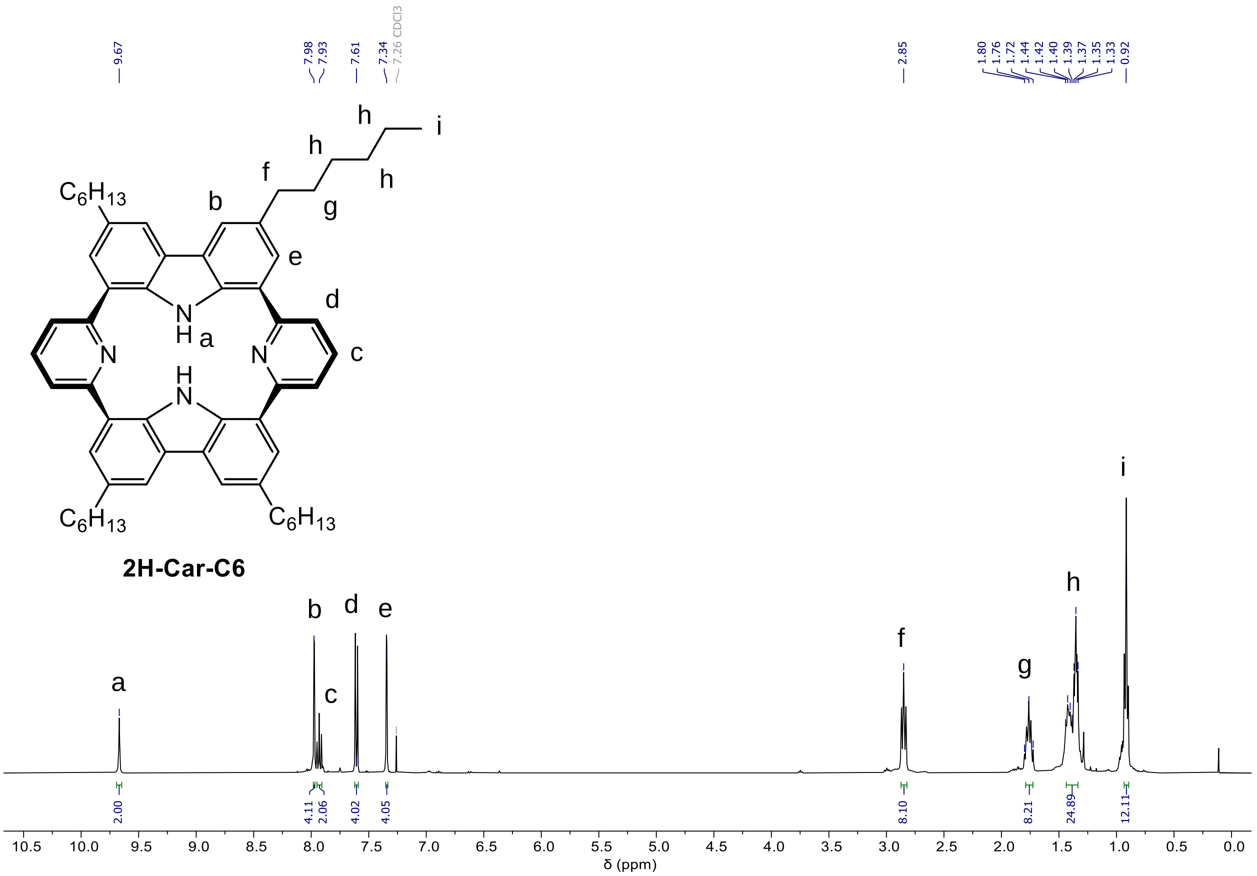

Supplementary Figure 4. ^{1}H NMR of **2H-Car-C6** (400 MHz, 298 K, $CDCl_3$).

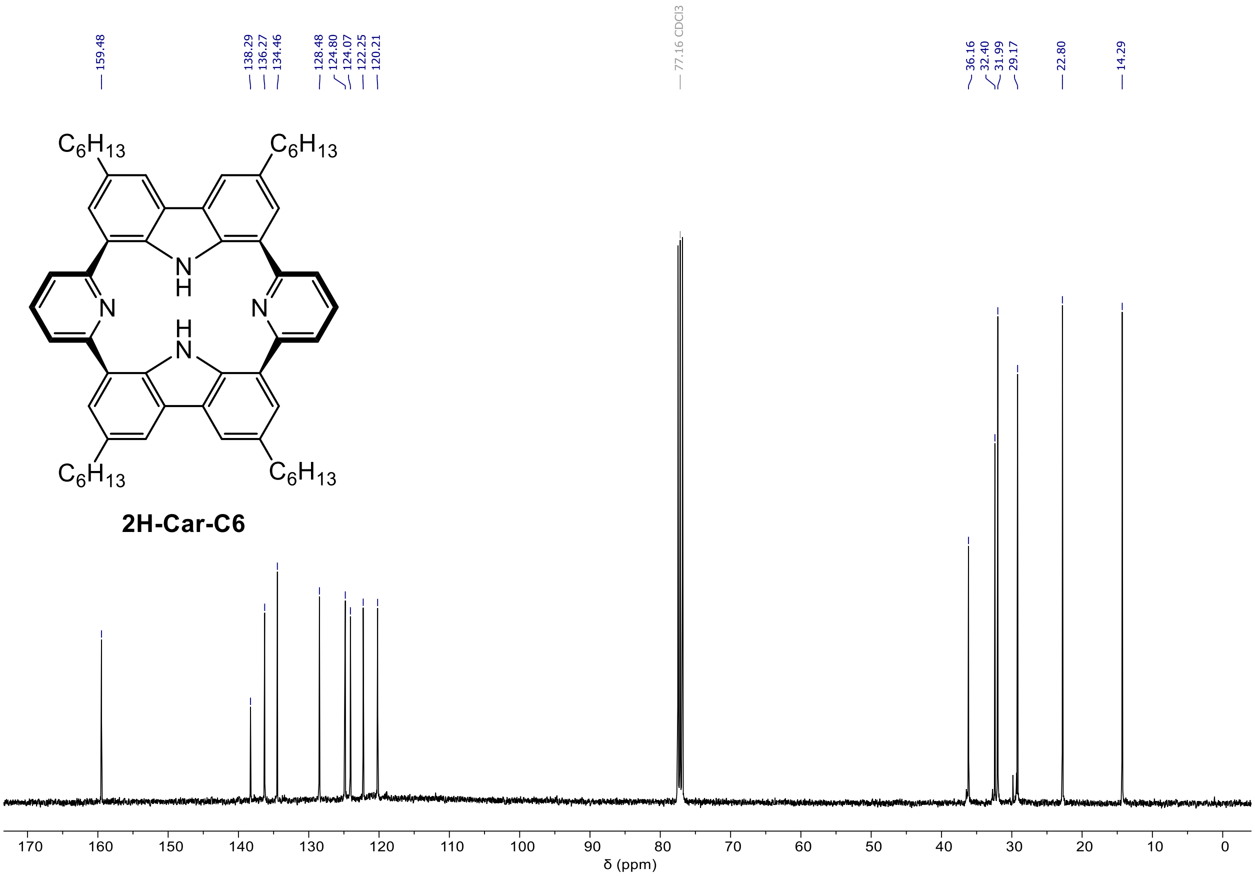

Supplementary Figure 5. ^{13}C NMR of **2H-Car-C6** (101 MHz, 298 K, $CDCl_3$).

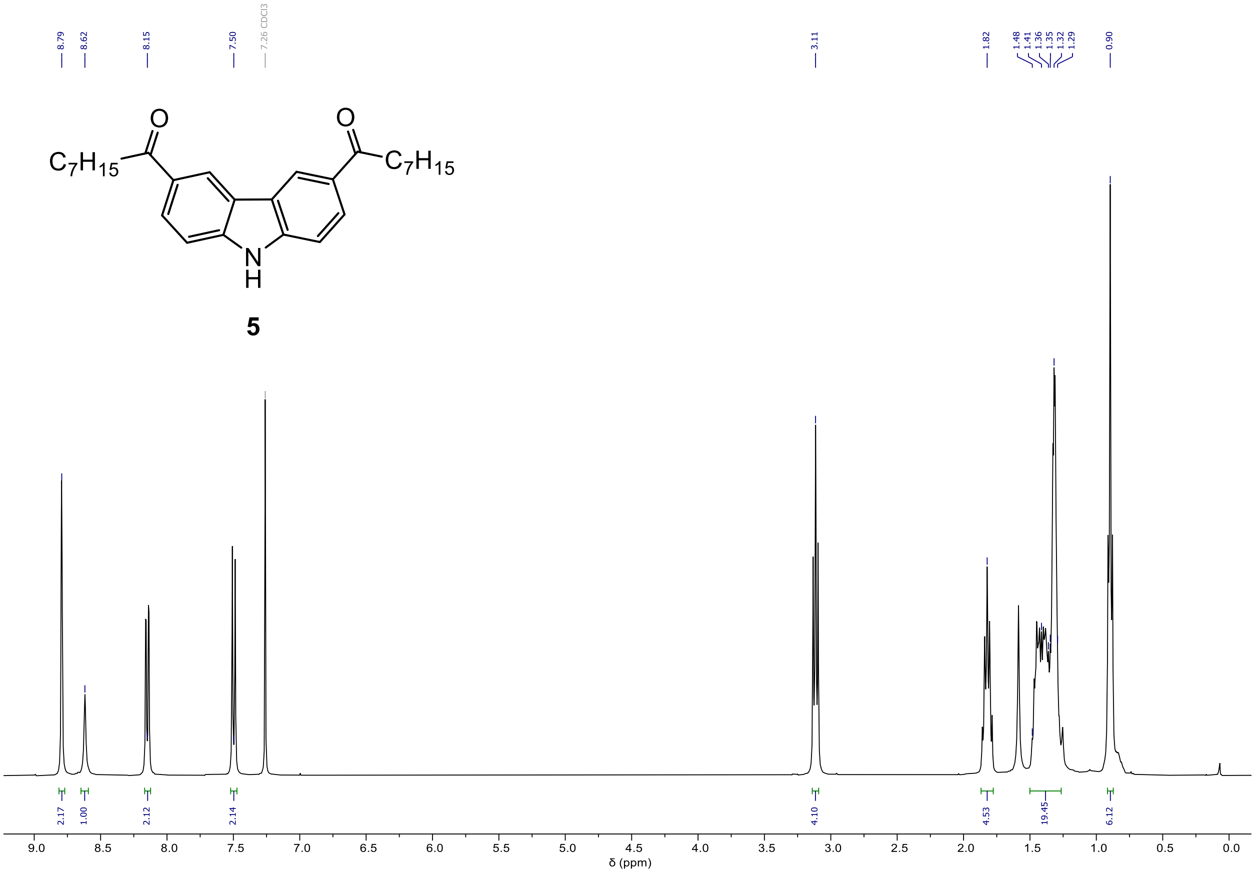

Supplementary Figure 6. ^{1}H NMR of **5** (400 MHz, 298 K, $CDCl_3$).

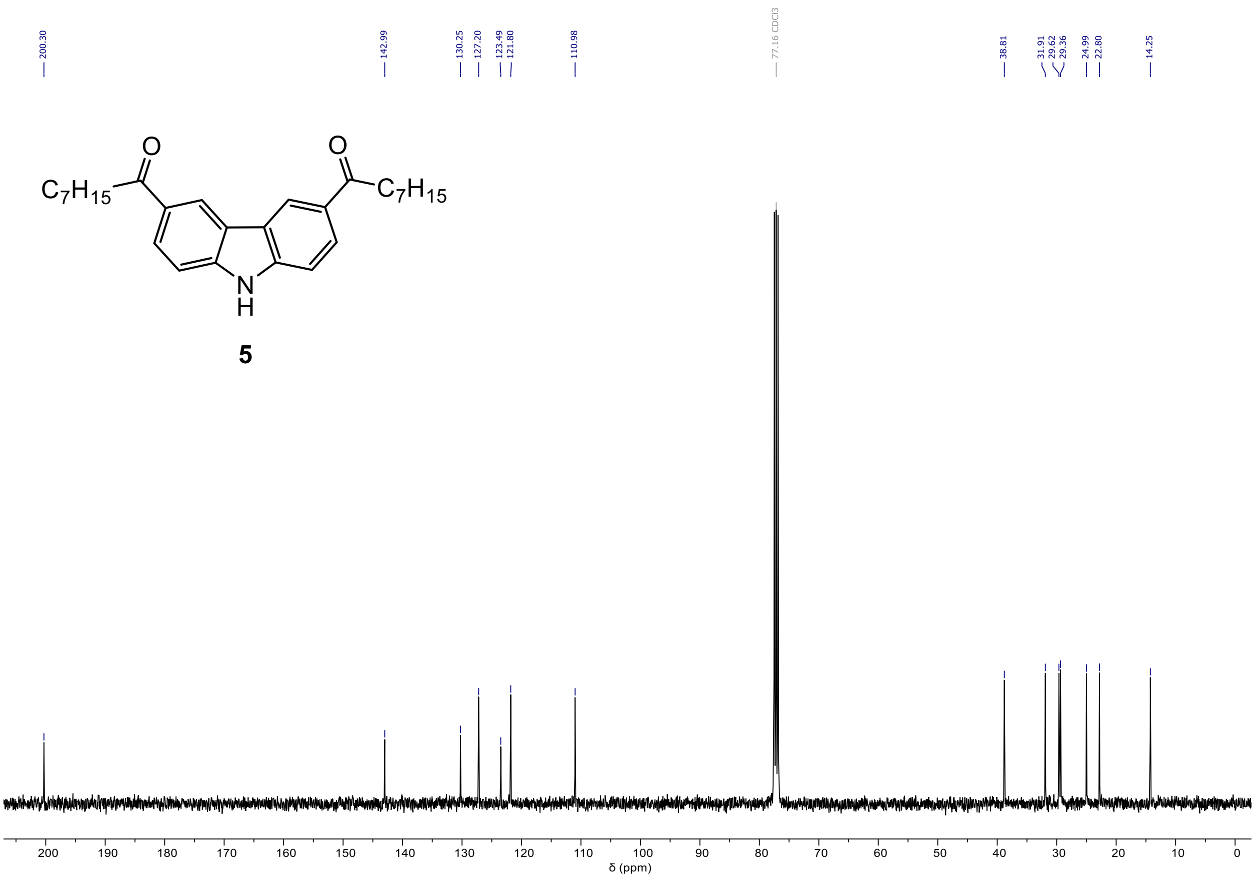

Supplementary Figure 7. ^{13}C NMR of **5** (400 MHz, 298 K, $CDCl_3$).

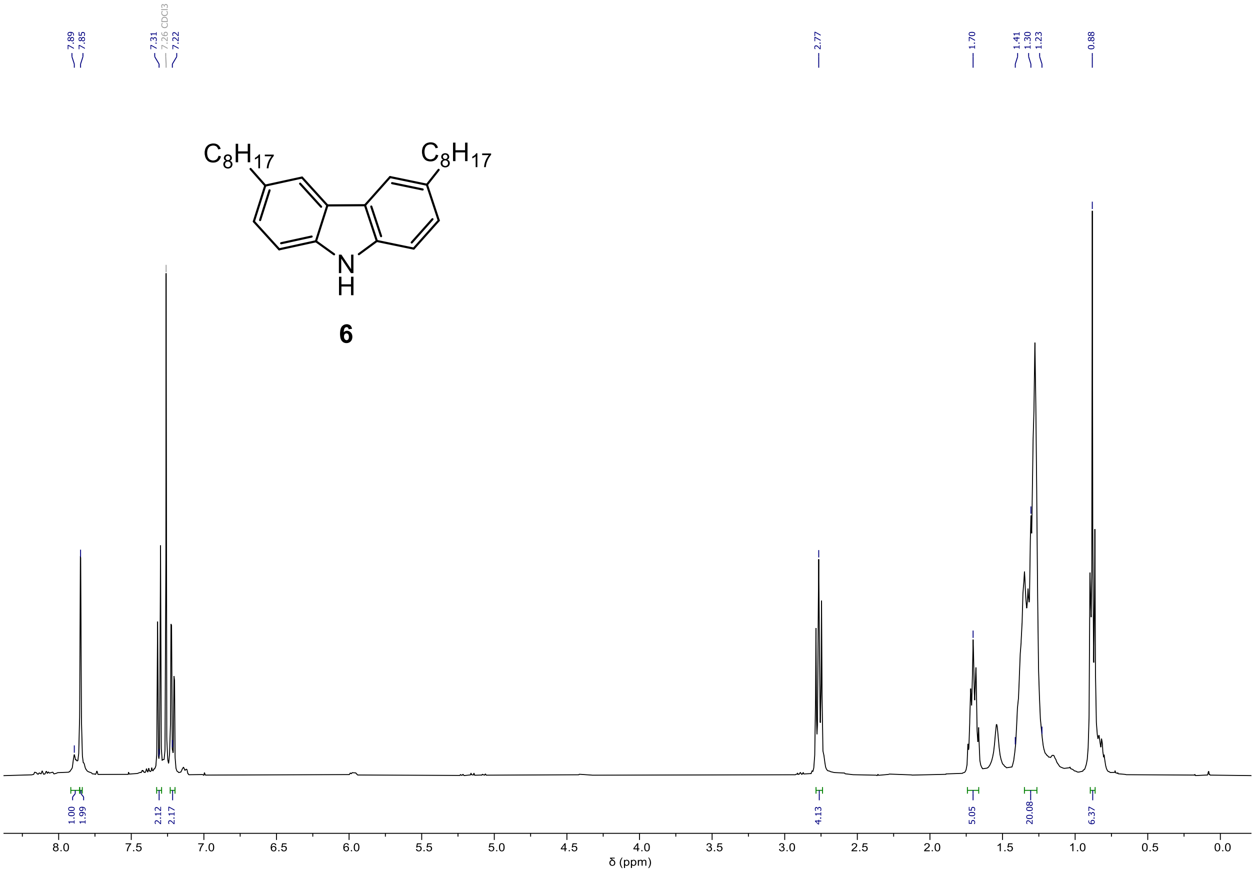

Supplementary Figure 8. ^{1}H NMR of **6** (400 MHz, 298 K, $CDCl_3$).

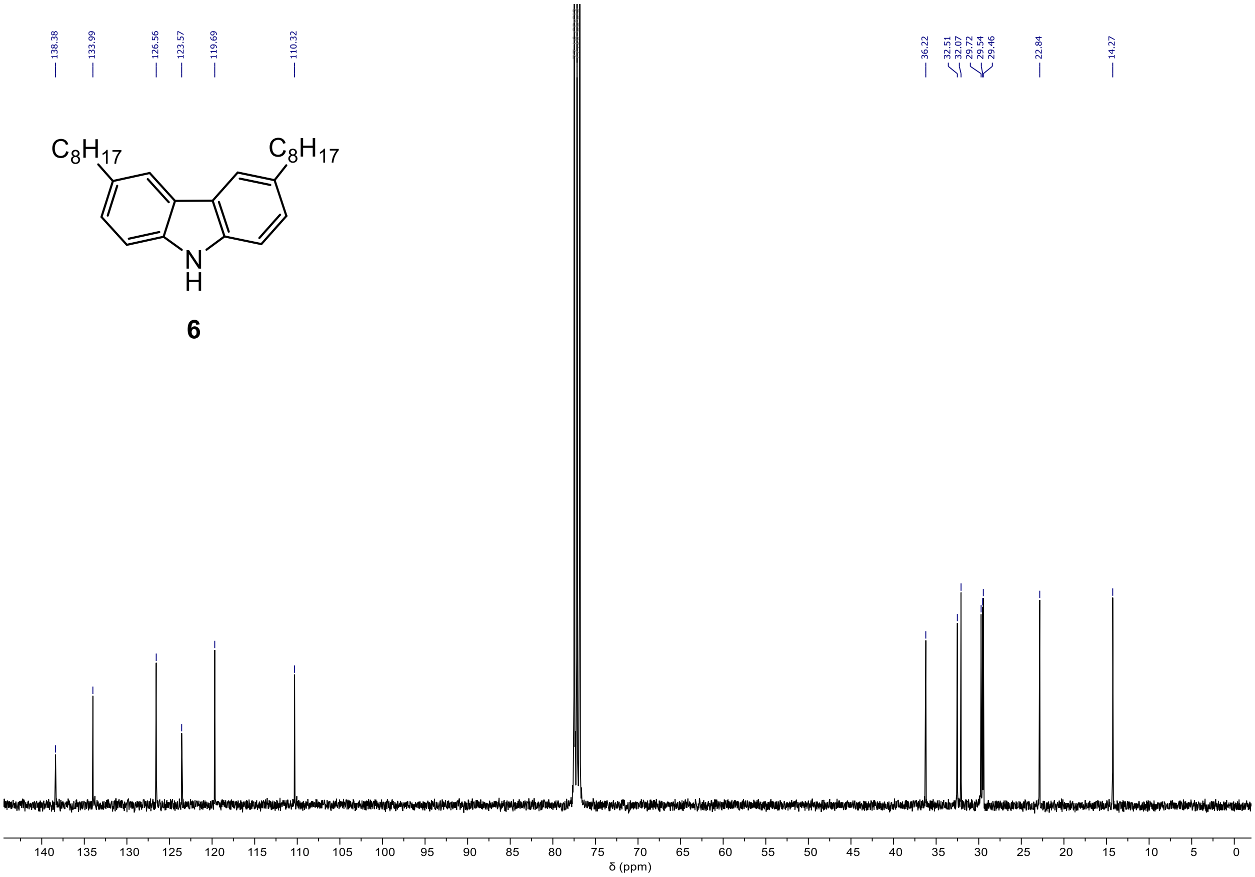

Supplementary Figure 9. ^{13}C NMR of **6** (400 MHz, 298 K, $CDCl_3$).

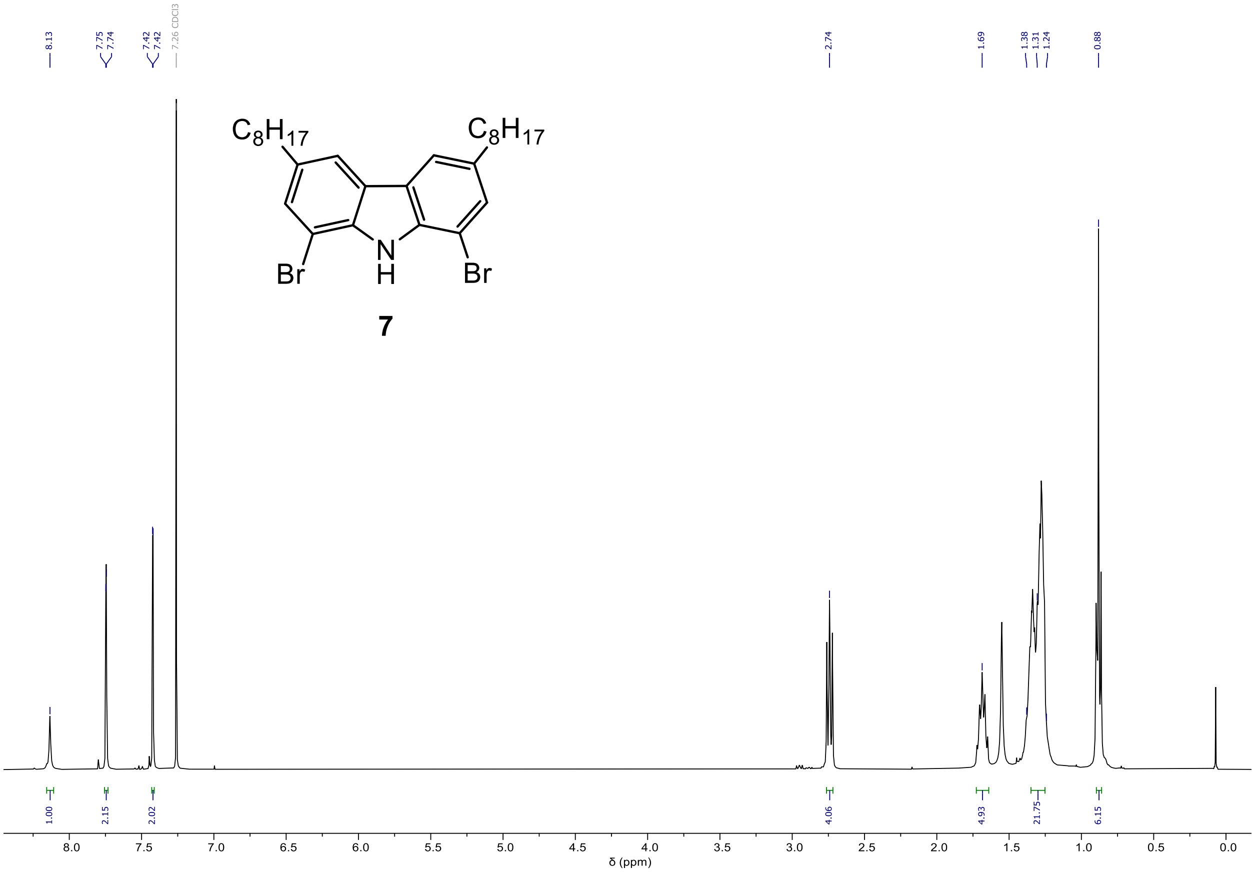

Supplementary Figure 10. ^{1}H NMR of **7** (400 MHz, 298 K, $CDCl_3$).

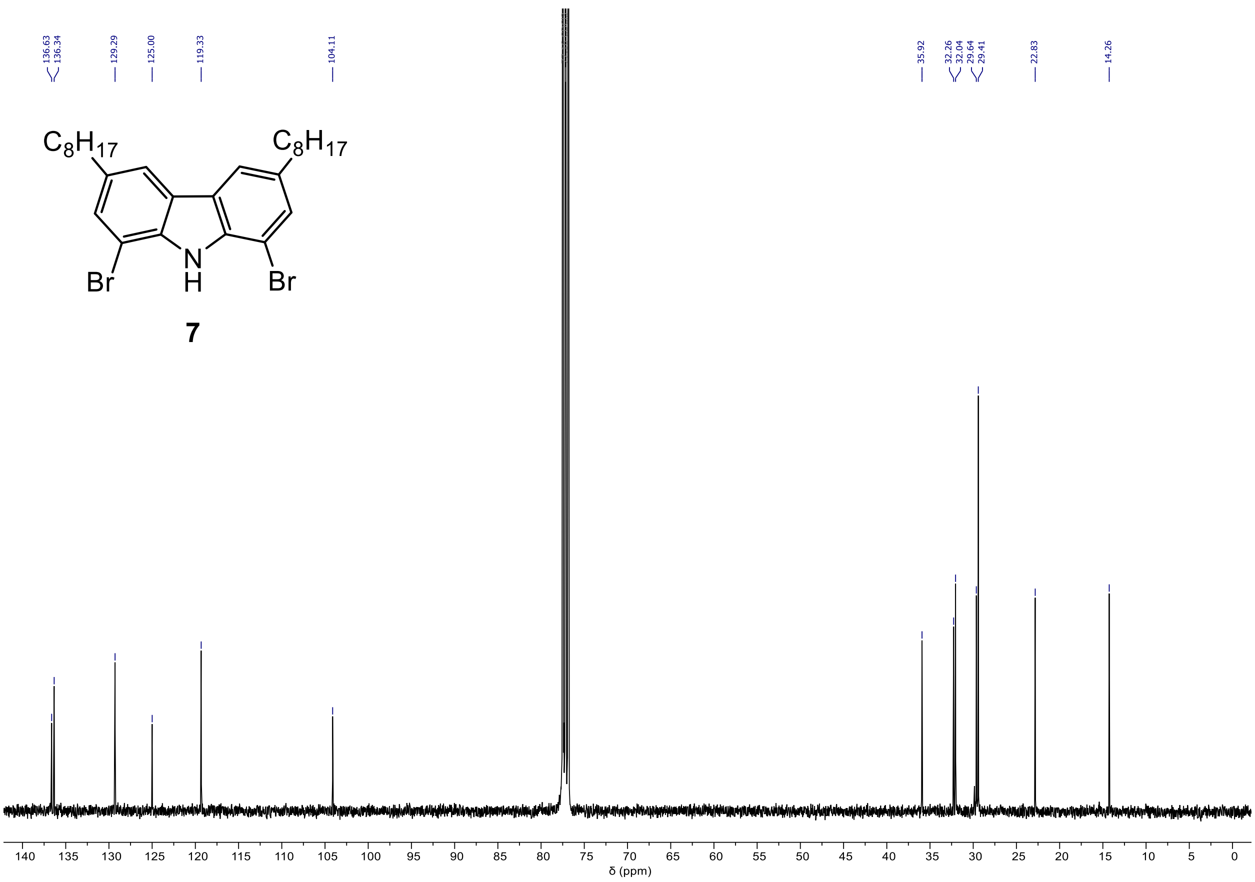

Supplementary Figure 11. ^{13}C NMR of **7** (400 MHz, 298 K, $CDCl_3$).

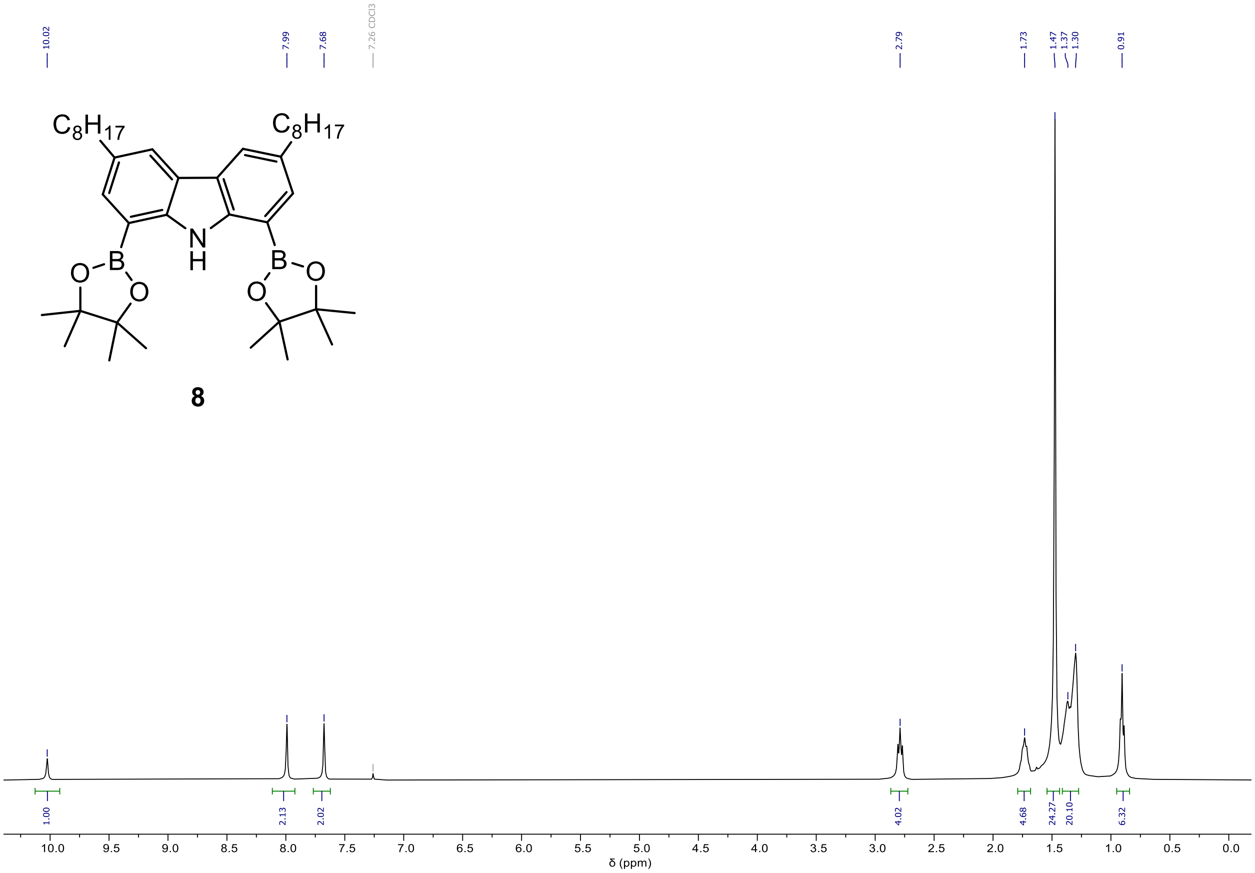

Supplementary Figure 12. ^{1}H NMR of **8** (400 MHz, 298 K, $CDCl_3$).

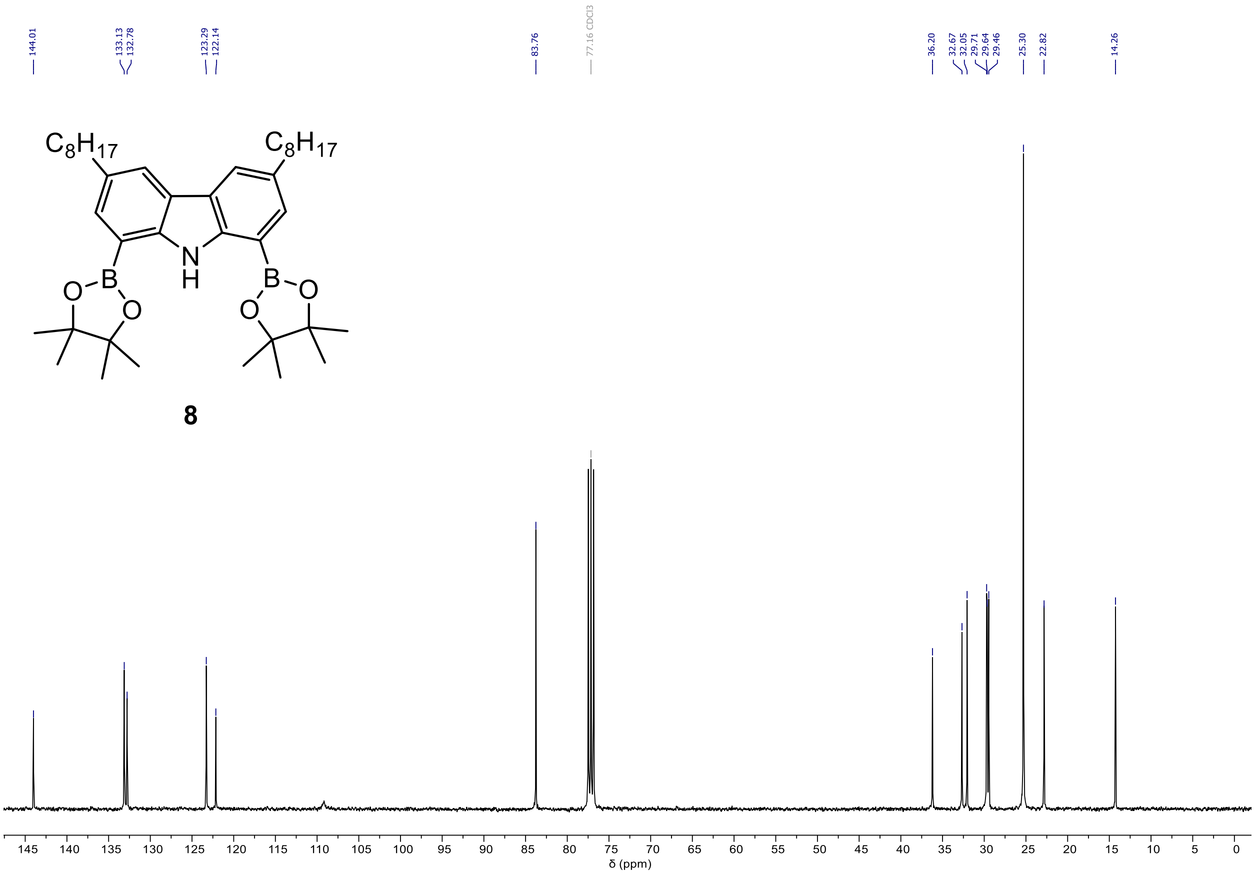

Supplementary Figure 13. ^{13}C NMR of **8** (400 MHz, 298 K, $CDCl_3$).

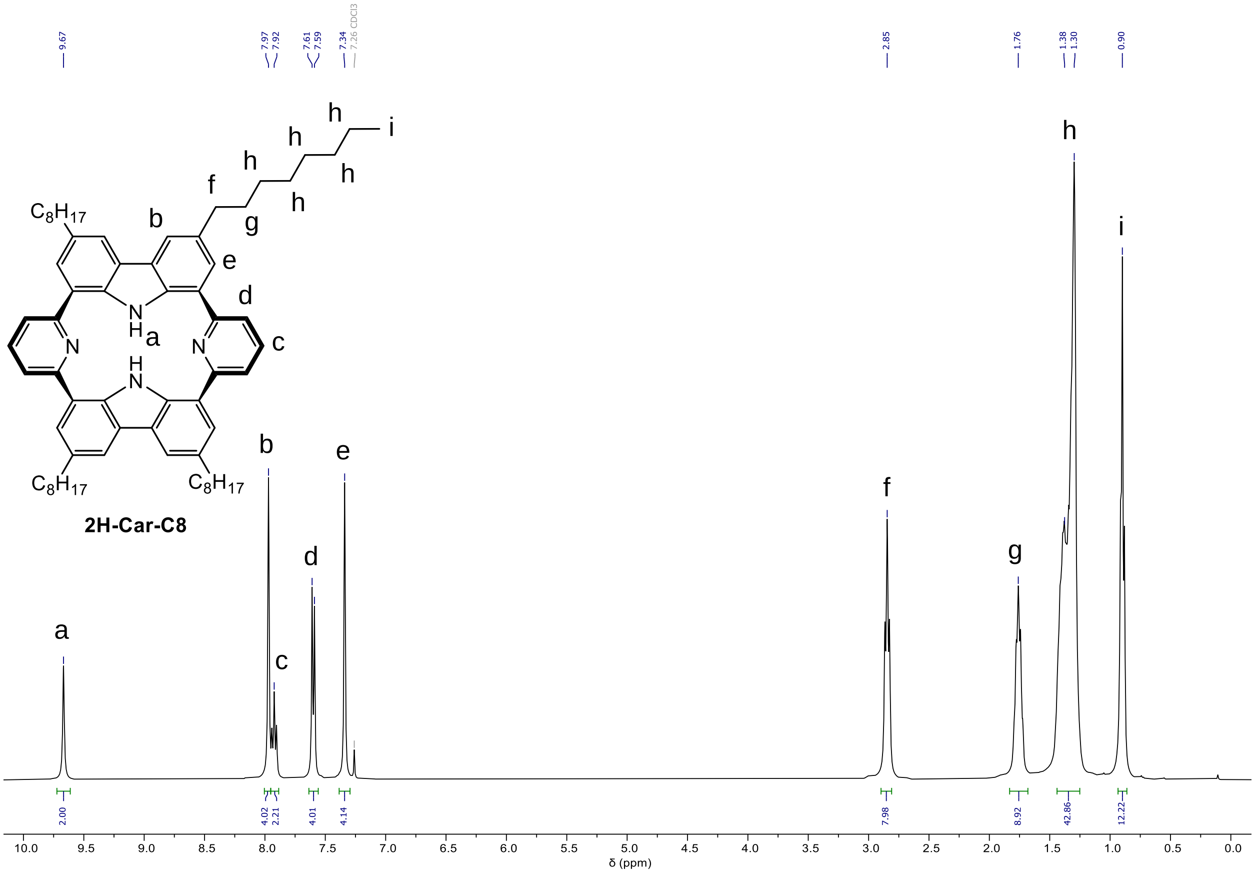

Supplementary Figure 14. ^{1}H NMR of **2H-Car-C8** (400 MHz, 298 K, $CDCl_3$).

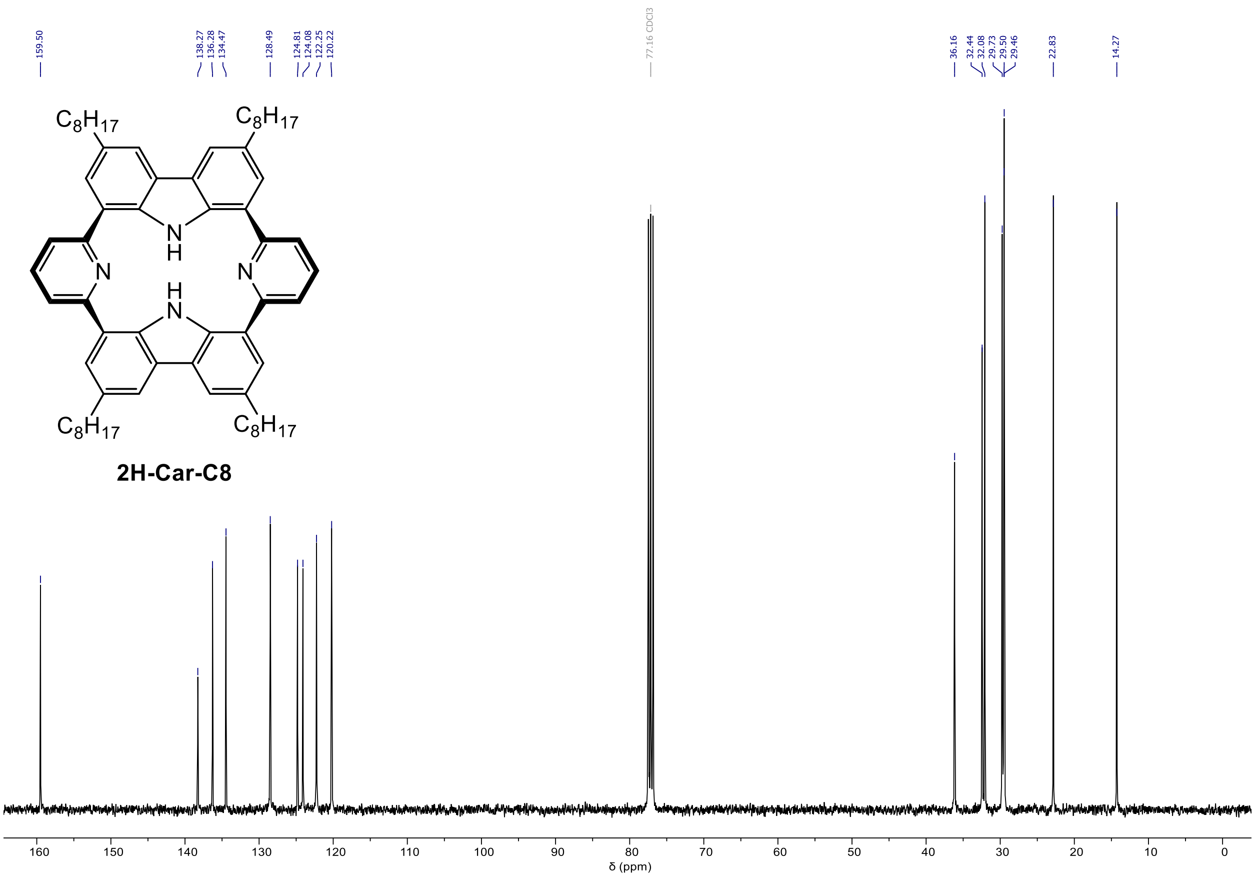

Supplementary Figure 15. ^{13}C NMR of **2H-Car-C8** (400 MHz, 298 K, $CDCl_3$).

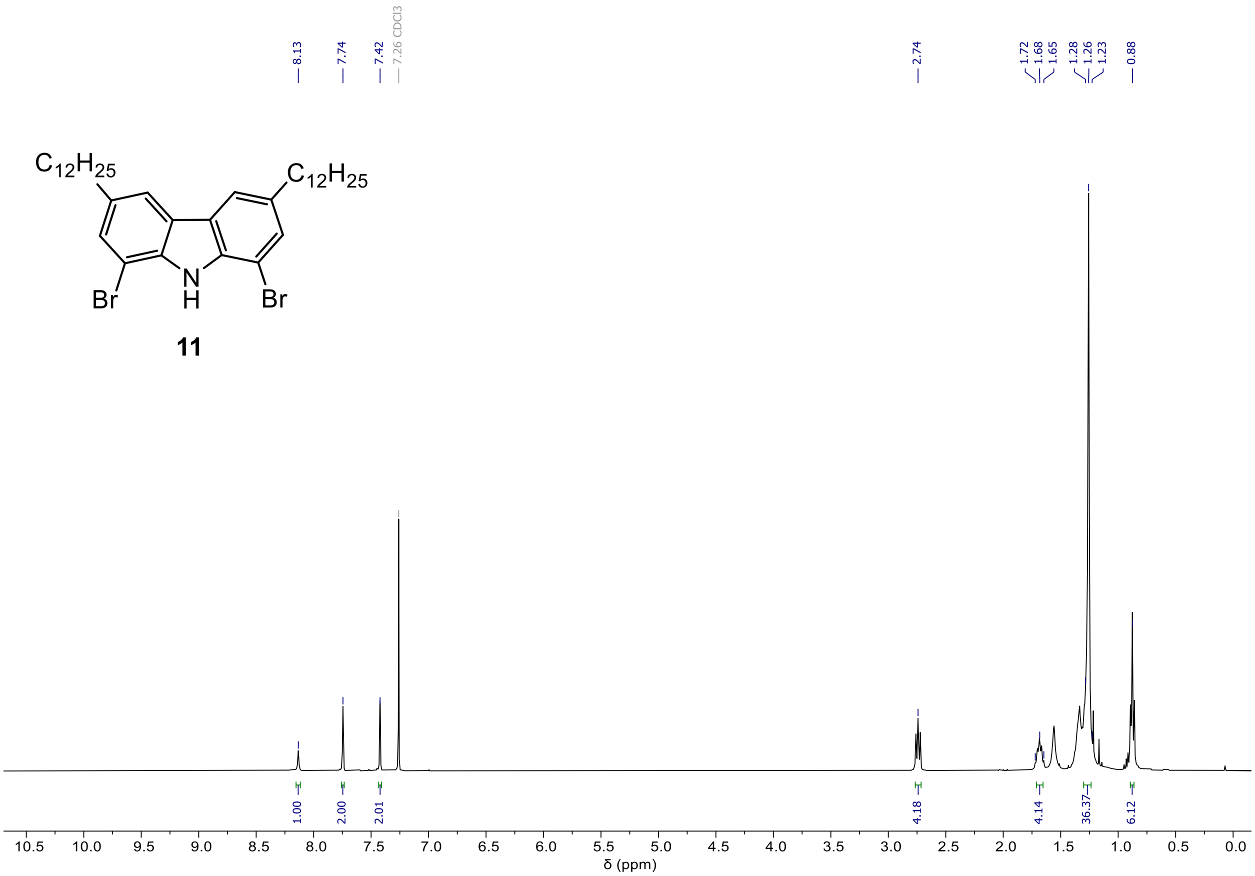

Supplementary Figure 16. ^{1}H NMR of **11** (400 MHz, 298 K, $CDCl_3$).

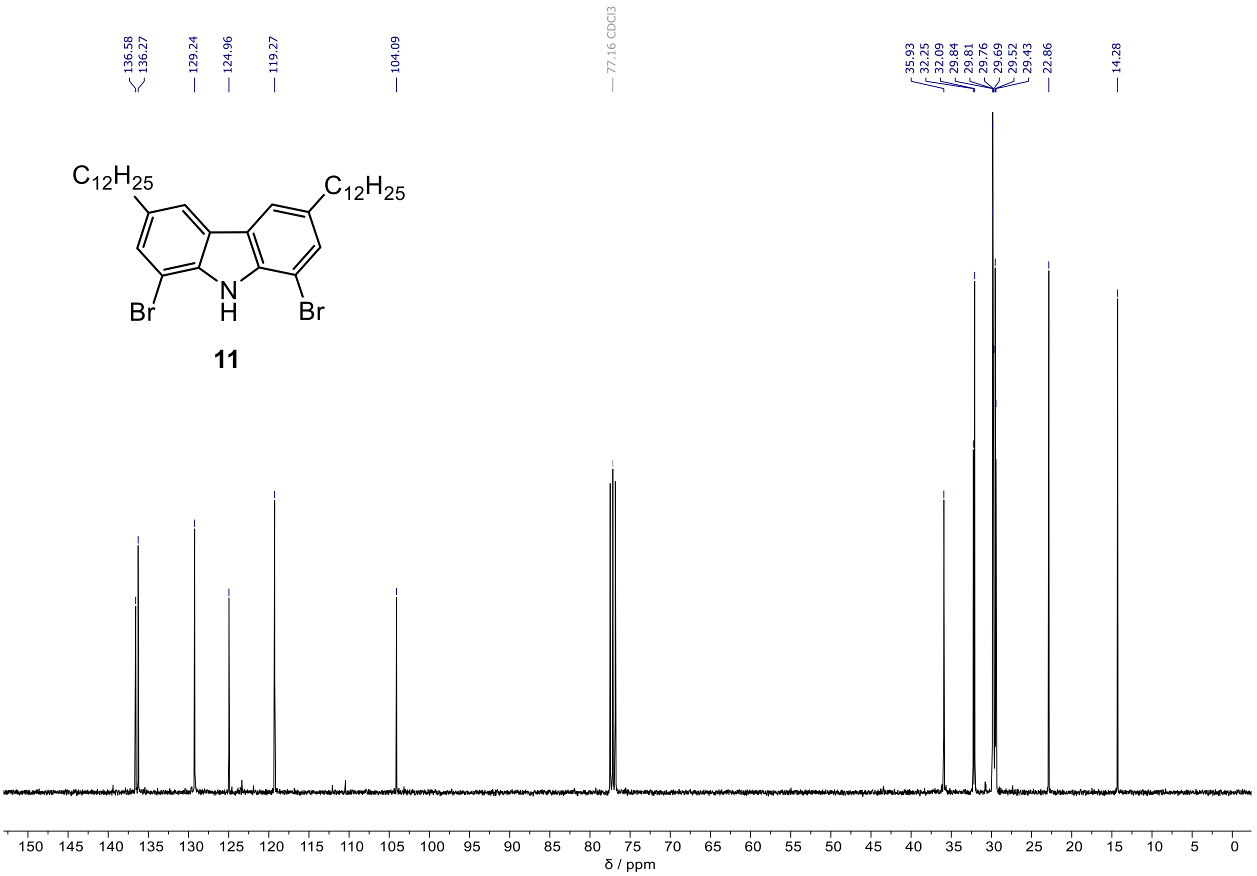

Supplementary Figure 17. ^{13}C NMR of **11** (101 MHz, 298 K, $CDCl_3$).

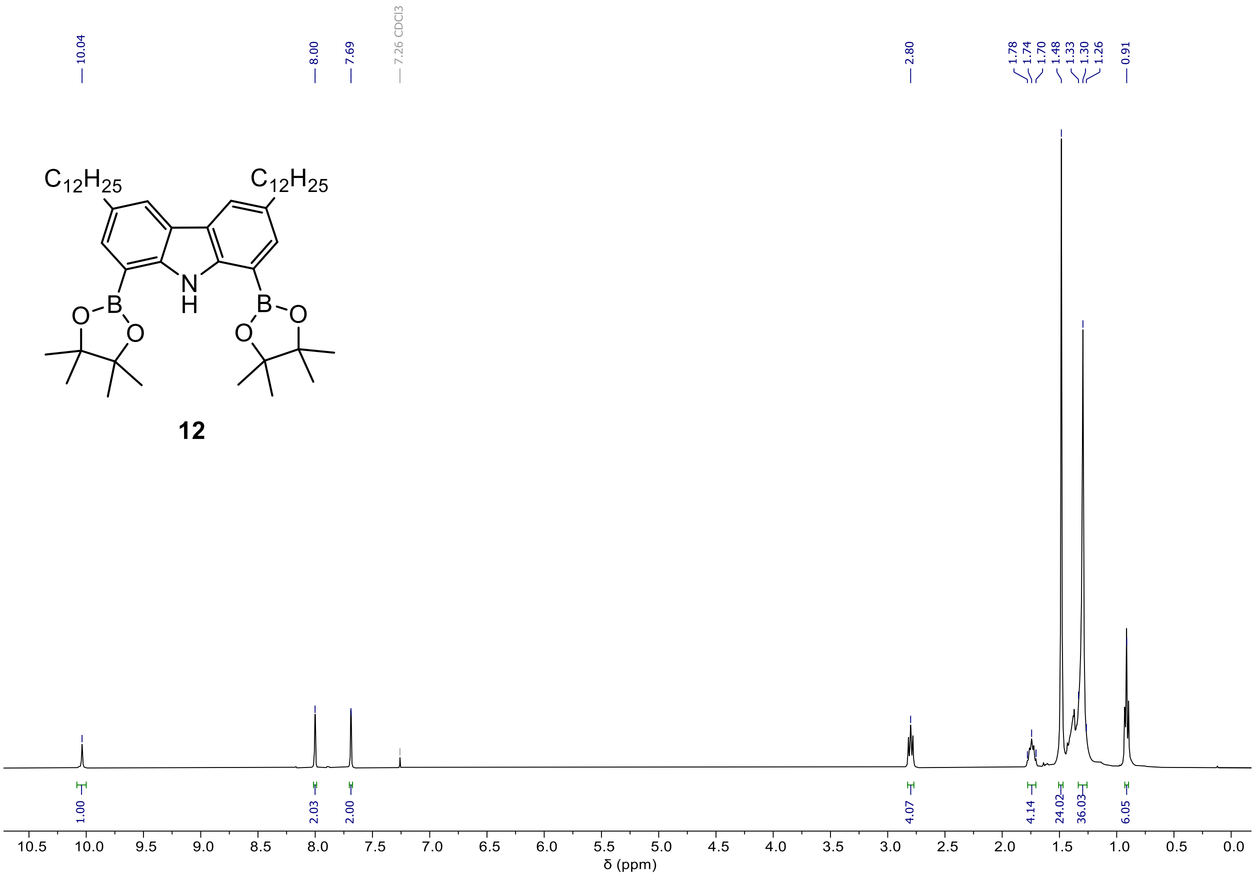

Supplementary Figure 18. ^{1}H NMR of **12** (400 MHz, 298 K, $CDCl_3$).

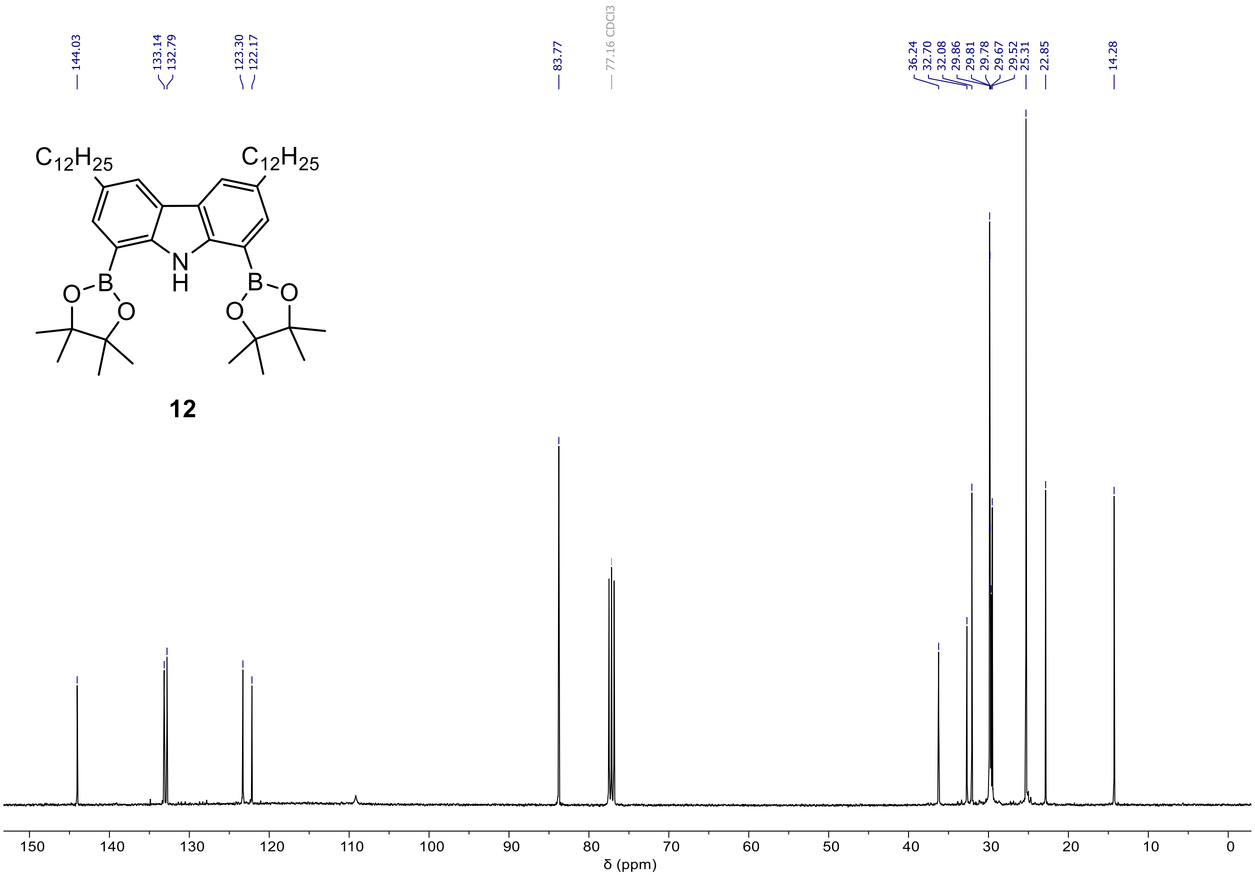

Supplementary Figure 19. ^{13}C NMR of **12** (101 MHz, 298 K, $CDCl_3$).

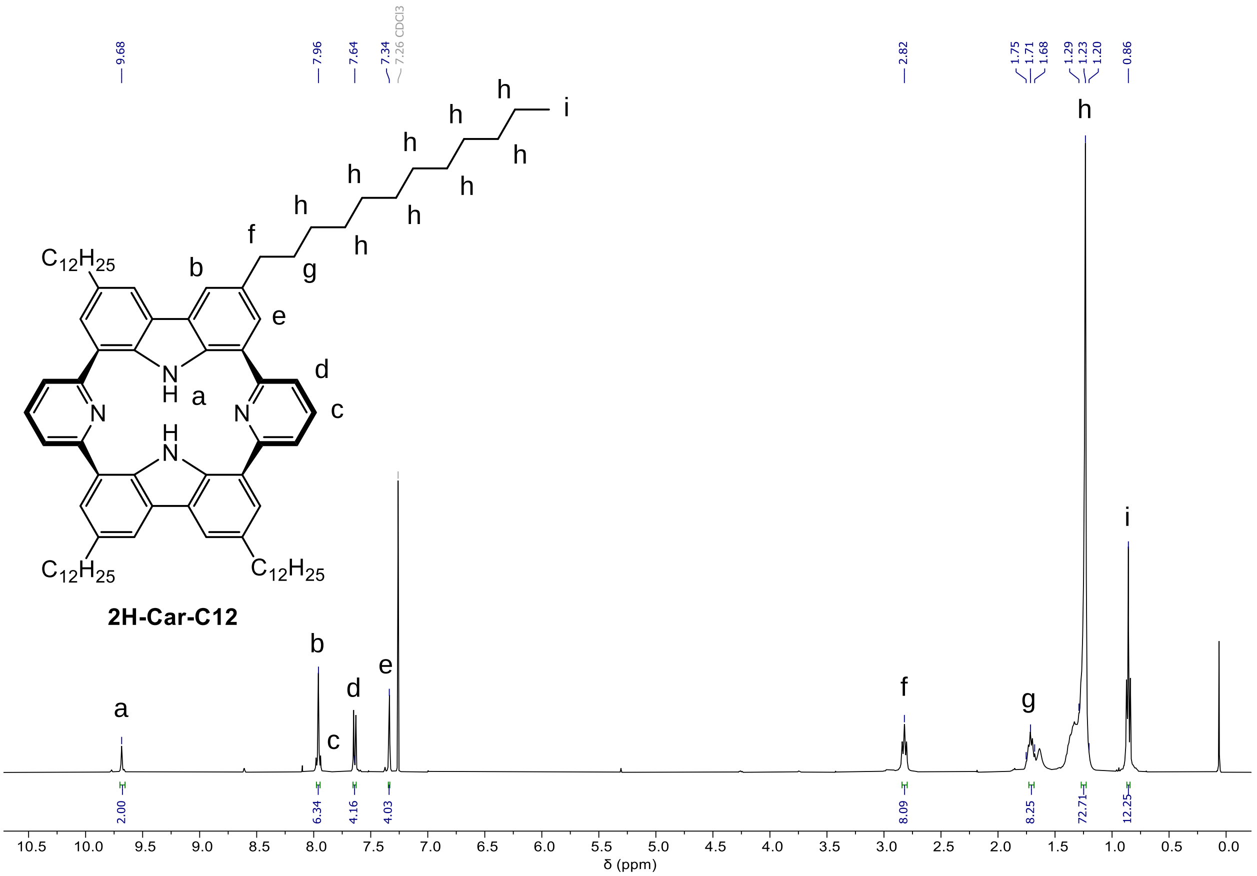

Supplementary Figure 20. ^{1}H NMR of **2H-Car-C12** (400 MHz, 298 K, $CDCl_3$).

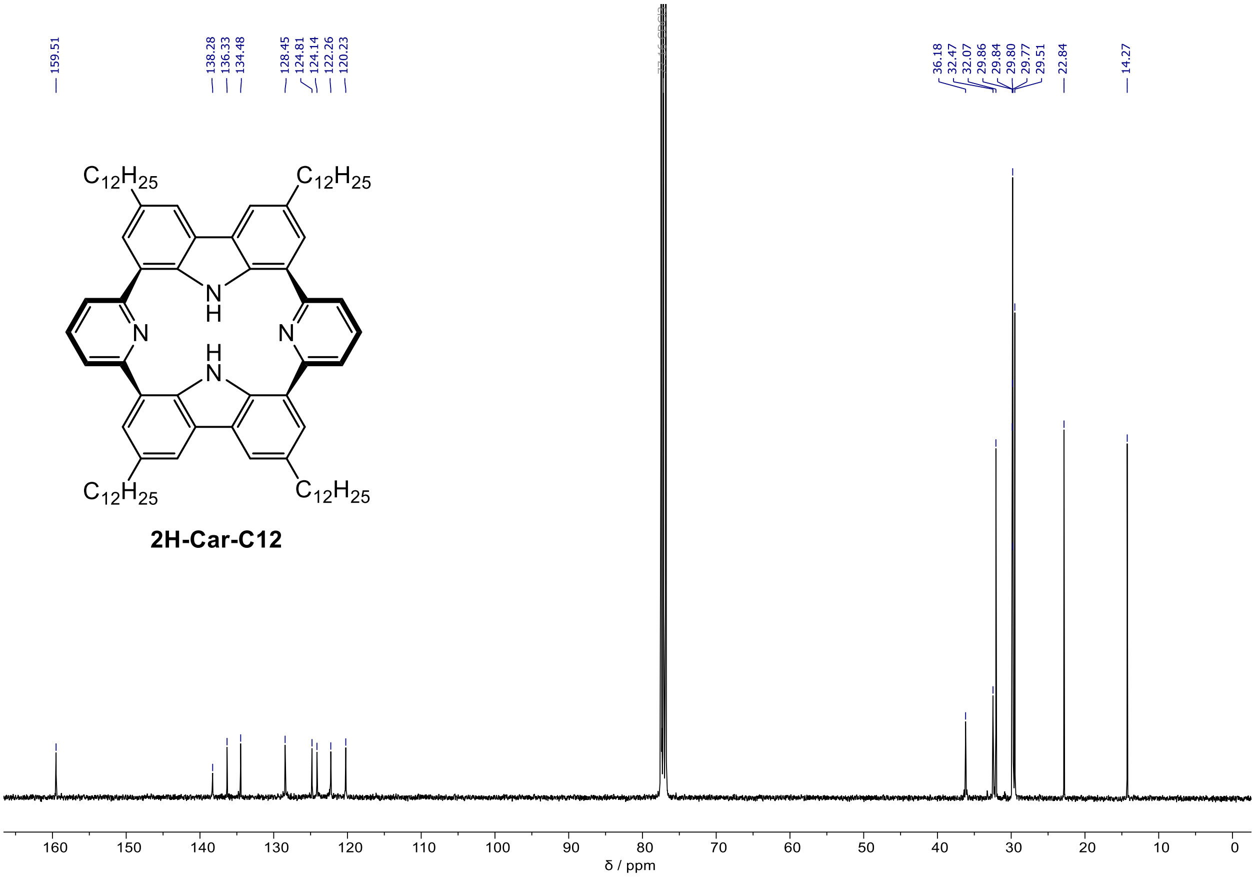

Supplementary Figure 21. ^{13}C NMR of **2H-Car-C12** (101 MHz, 298 K, $CDCl_3$).

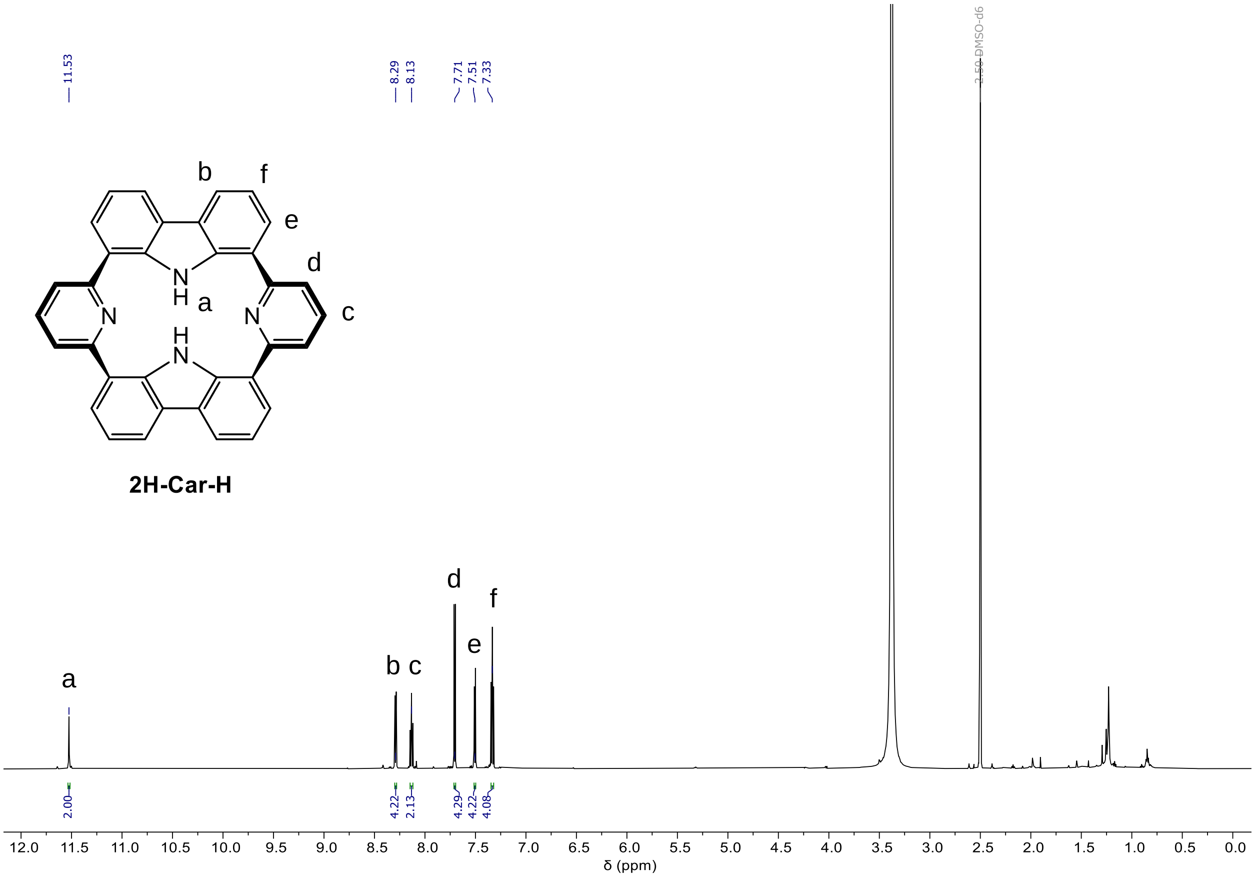

Supplementary Figure 22. ^{1}H NMR of **2H-Car-H** (600 MHz, 298 K, DMSO-d_6).

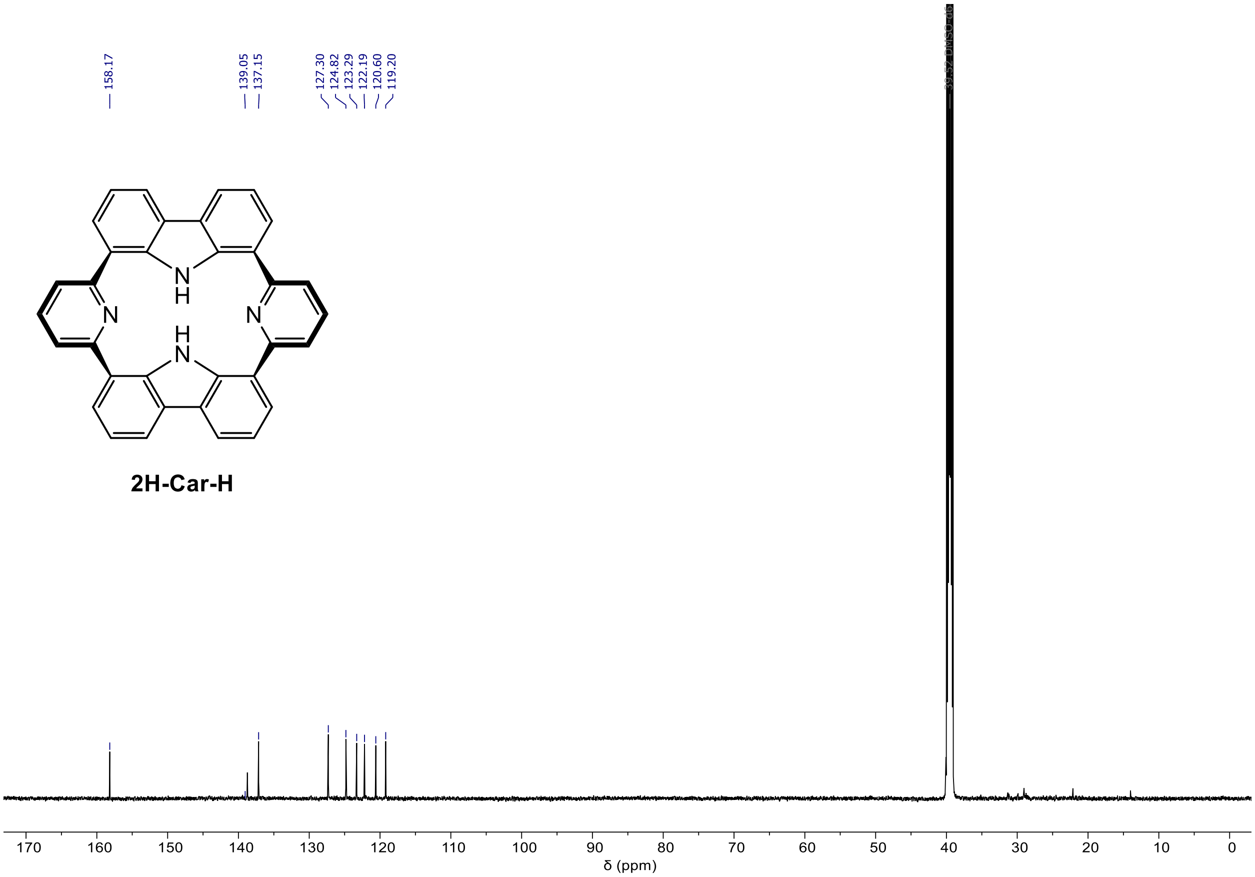

Supplementary Figure 23. ^{13}C NMR of **2H-Car-H** (151 MHz, 298 K, DMSO-d_6).

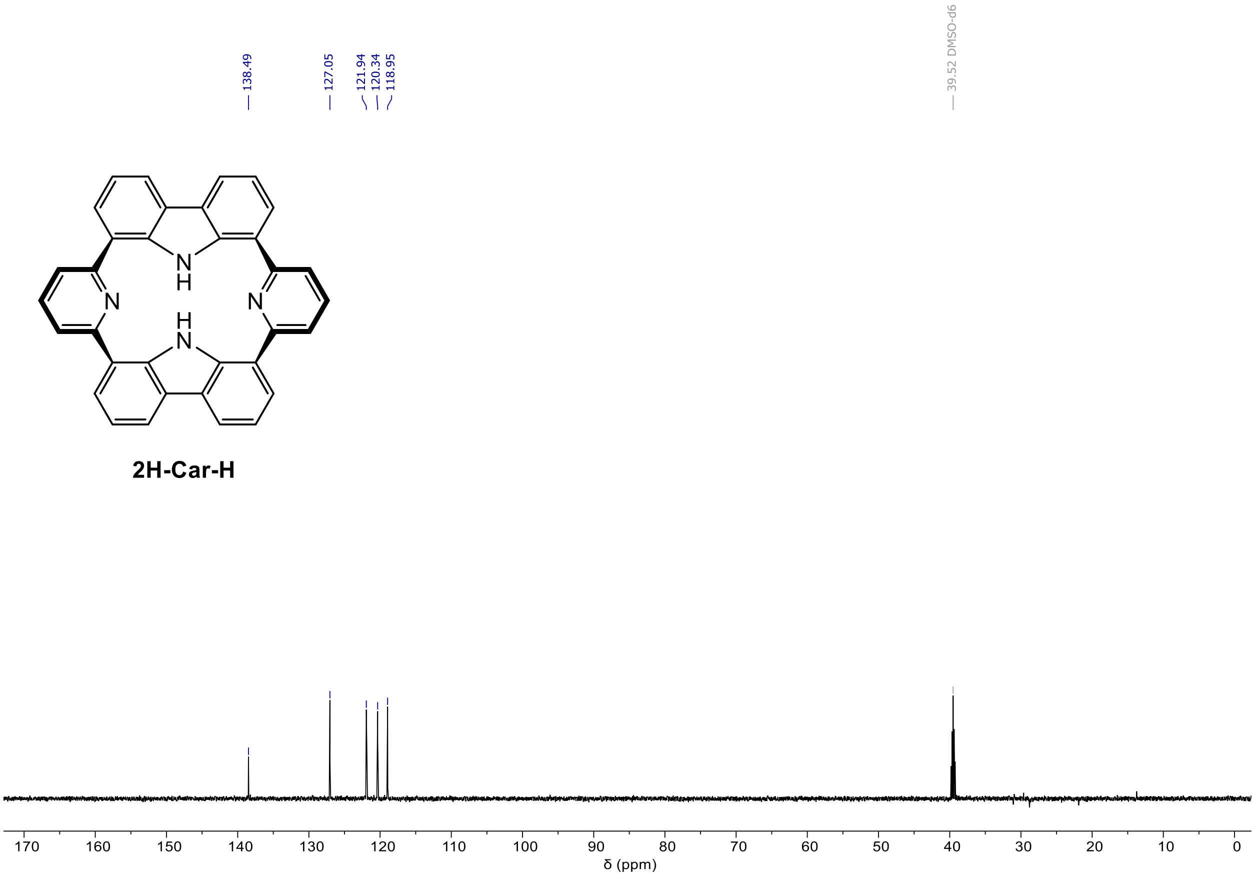

Supplementary Figure 24. DEPT-135 of **2H-Car-H** (151 MHz, 298 K, DMSO-d_6).

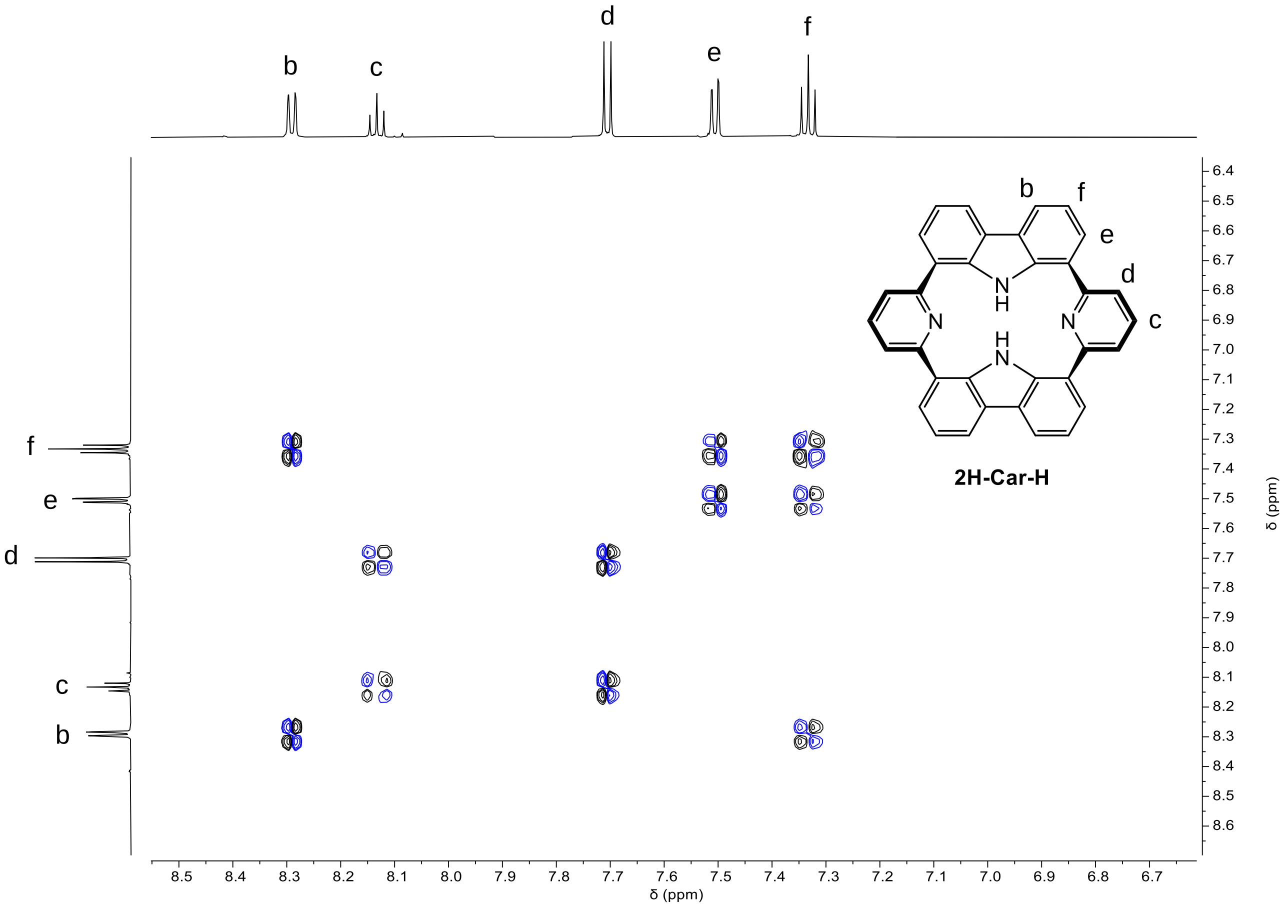

Supplementary Figure 25. Selected part of the ^{1}H-^{1}H COSY of **2H-Car-H** (600 MHz, 298 K, DMSO-d_6).

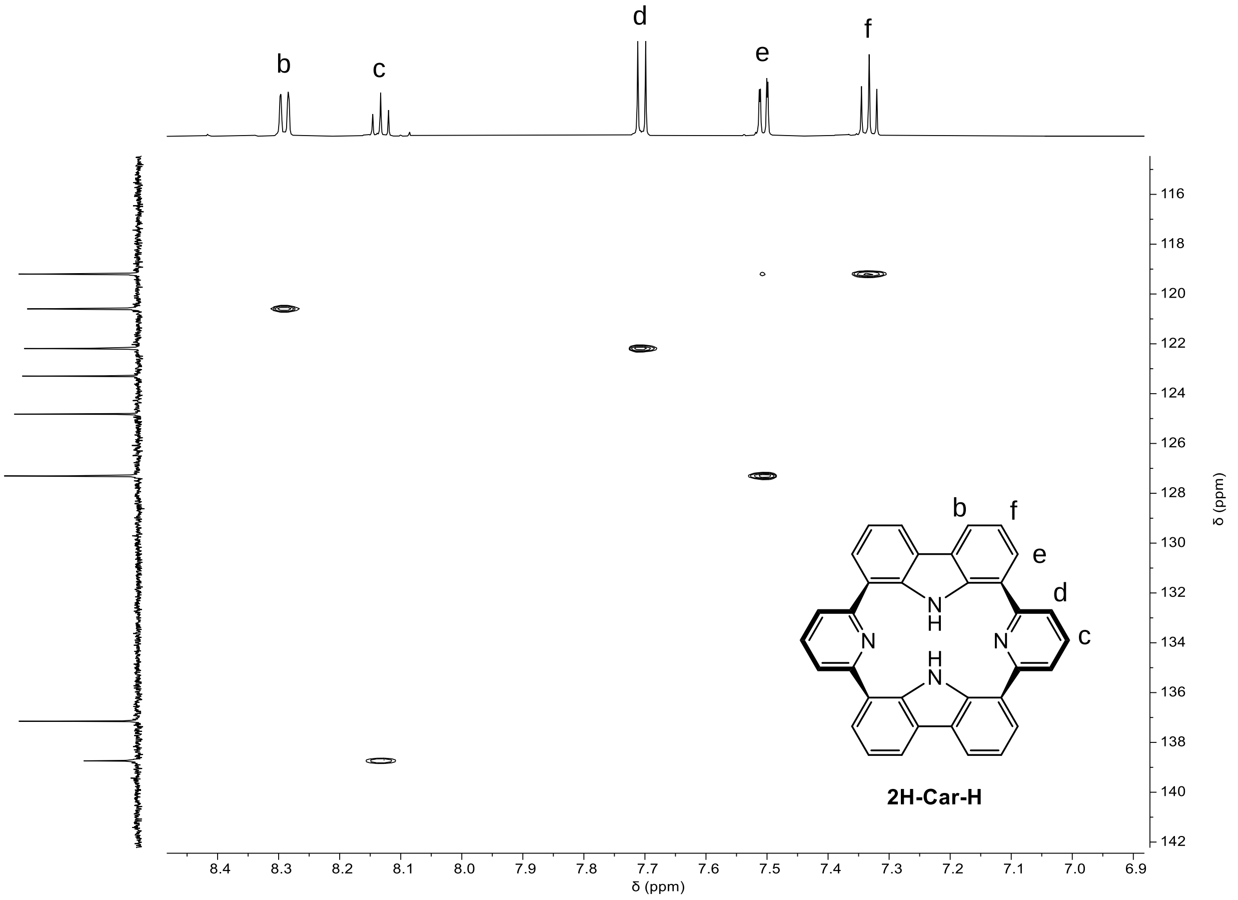

Supplementary Figure 26. Selected part of the HSQC of **2H-Car-H** (600 MHz, 298 K, DMSO-*d*$_6$).

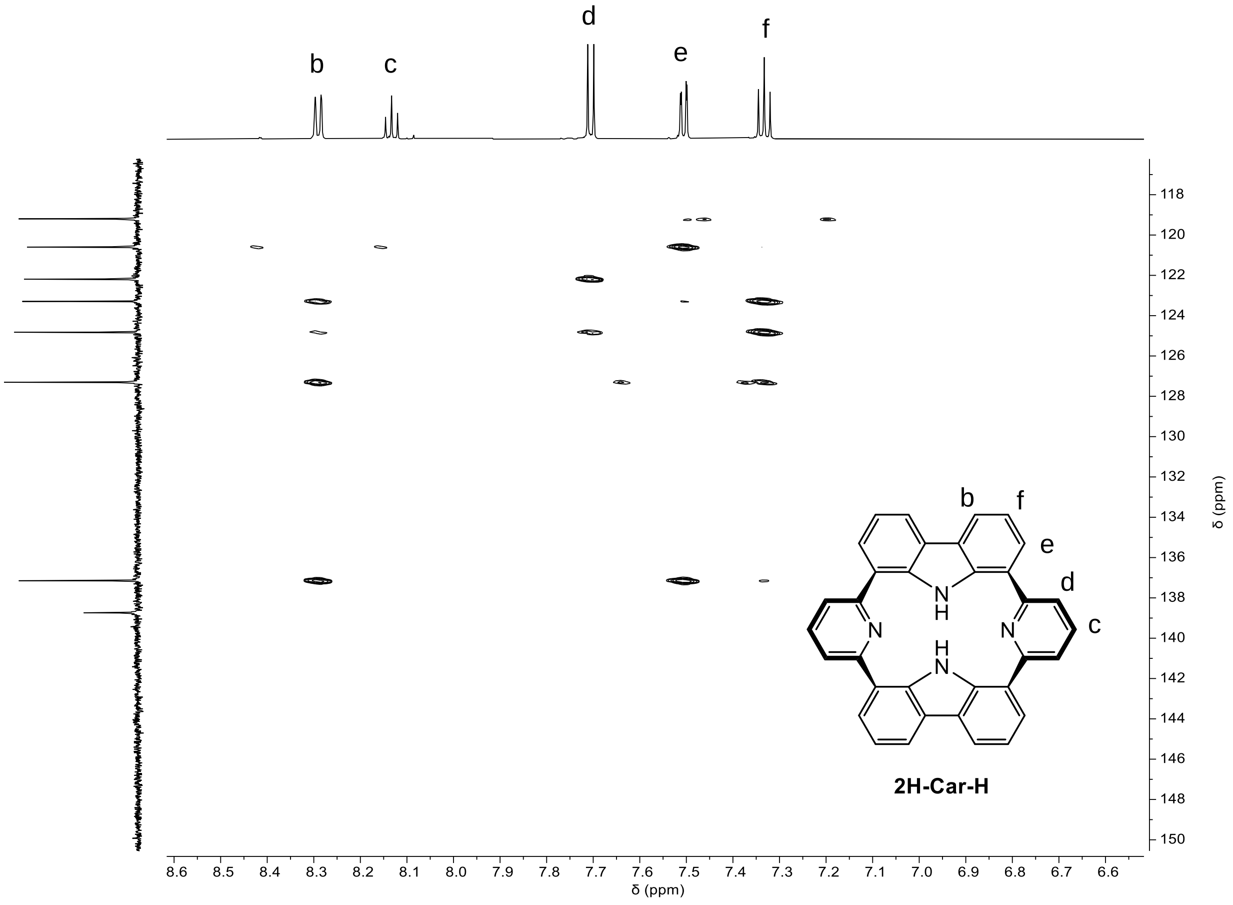

Supplementary Figure 27. Selected part of the HMBC of **2H-Car-H** (600 MHz, 298 K, DMSO-d_6).

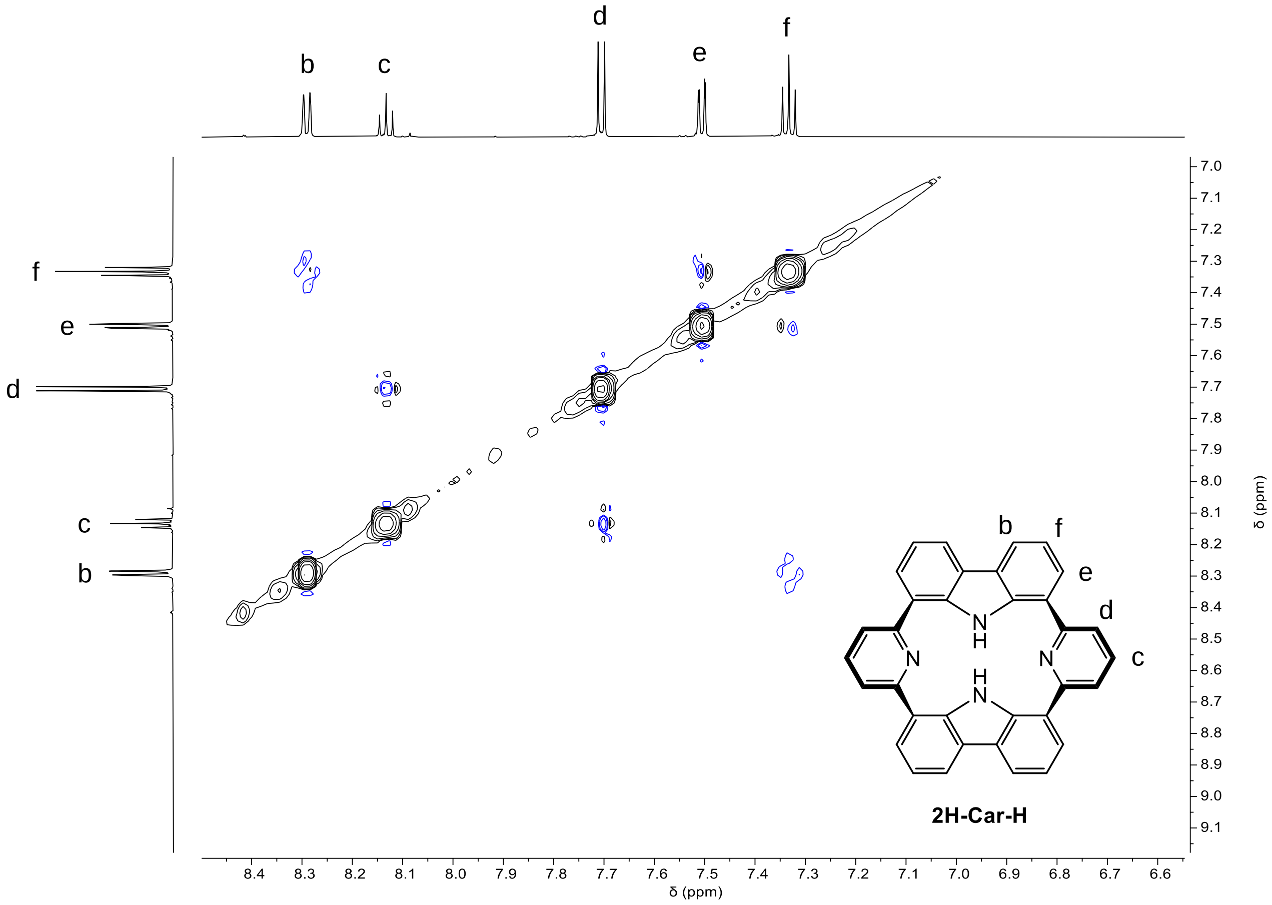

Supplementary Figure 28. Selected part of the NOESY of **2H-Car-H** (600 MHz, 298 K, DMSO-d_6).

Variable Temperature ^{1}H NMR Spectra

Supplementary Table 1a. Summary of ^{1}H NMR (500 MHz, toluene-d_8, 9.25 mM) chemical shift variation of **2H-Car-C6** aromatic region with respect to temperature. NH (a) and 4-pyridyl (c) resonances experience the largest changes in chemical shift.

2H-Car-C6

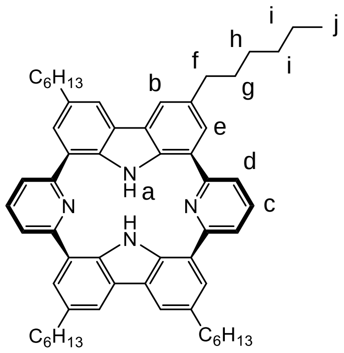

NMR shifts (center of signal) of **2H-Car-C6** aromatic region in toluene-d_8 (δ; Δδ[a]) in ppm

T / K	a	b	c	d	e
**343**	9.79; +0.01	8.00[b]; −0.02	7.47; +0.05	7.42; +0.01	7.37[b]; −0.01
**333**	9.79	8.00[b]	7.46; +0.04	7.42	7.37
**323**	9.79	8.00[b]	7.46	7.42	7.37
**313**	9.79	8.01[b]; −0.01	7.45; +0.03	7.41; 0.00	7.37
**303**	9.78; 0.00	8.01[b]	7.44; +0.02	7.41	7.38; 0.00
**293**	9.78	8.02[b]; 0.00	7.42; 0.00	7.41	7.38
**283**	9.77[b]; −0.01	8.02[b]	7.41[c]; −0.01	7.41[c]	7.38
**273**	9.77[b]	8.02[b]	7.40[b,c]; −0.02	7.40[b,c]; −0.01	7.39; +0.01
**263**	9.76[b]; −0.02	8.03[b]; +0.01	7.40[b,c]	7.40[b,c]	7.39[b]
**253**	9.74[b]; −0.04	8.03[b]	7.39[b,c]; −0.03	7.39[b,c]; −0.02	7.39[b,c]
**243**	9.72[b]; **−0.06**	8.04[b]; +0.02	7.39[b,c]	7.39[b,c]	7.39[b,c]
**233**	9.70[b]; **−0.08**	8.04[b]	7.35[b,c]; −0.05	7.39[b,c]	7.40[b,c]; +0.02
**223**	9.69[b]; **−0.09**	8.04[b]	7.33[b,c]; **−0.09**	7.39[b,c]	7.40[b,c]
**213**	9.67[b]; **−0.11**	8.04[b]	7.31[b,c]; **−0.11**	7.38[b,c]; −0.03	7.40[b,c]
**203**	9.65[b]; **−0.13**	8.04[b]	7.29[b,c]; **−0.13**	7.38[b,c]	7.40[b,c]
**196**	9.64[b]; **−0.14**	8.04[b]	7.27[b,c]; **−0.15**	7.36[b,c]; −0.05	7.40[b,c]

[a] Δδ referenced to 293 K, [b] broad signal, [c] assignment unclear.

Supplementary Table 1b. Summary of ^{1}H NMR (500 MHz, toluene-d_8, 9.25 mM) chemical shift variation of **2H-Car-C6** aliphatic region with respect to temperature.

2H-Car-C6

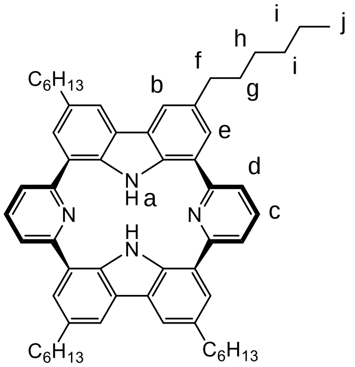

NMR shifts (center of signal) of **2H-Car-C6** aliphatic region in toluene-d_8 (δ; Δδ[a]) in ppm

T / K	f	g	h	i	j
**343**	2.86; −0.01	1.80; +0.01	1.46; +0.01	1.34; 0.00	0.90; −0.03
**333**	2.86	1.80	1.46	1.34	0.91; −0.02
**323**	2.86	1.80	1.46	1.34	0.91
**313**	2.87; 0.00	1.79; 0.00	1.46	1.34	0.92; −0.01
**303**	2.87	1.79	1.46	1.34	0.92
**293**	2.87	1.79	1.45; 0.00	1.34	0.93; 0.00
**283**	2.87	1.79	1.45	1.34[b]	0.93
**273**	2.87	1.79	1.45[b]	1.34[b]	0.94; +0.01
**263**	2.87	1.78; −0.01	1.45[b]	1.34[b]	0.95; +0.02
**253**	2.87	1.78	1.45[b]	1.33[b]; −0.01	0.96; +0.03
**243**	2.88; +0.01	1.78	1.44[b]; −0.01	1.33[b]	0.96[b]
**233**	2.88[b]	1.77[b]; −0.02	1.44[b]	1.33[b]	0.97[b]; +0.04
**223**	2.88[b]	1.77[b]	1.43[b]; −0.02	1.32[b]; −0.02	0.98[b]; +0.05
**213**	2.88[b]	1.76[b]; −0.03	1.43[b]	1.32[b]	0.99[b]; **+0.06**
**203**	2.88[b]	1.75[b]; −0.04	1.42[b]; −0.03	1.31[b]; −0.03	0.99[b]
**196**	2.88[b]	1.75[b]	1.41[b]; −0.04	1.30[b]; −0.04	1.00[b]; **+0.07**

[a] Δδ referenced to 293 K, [b] broad signal, [c] assignment unclear.

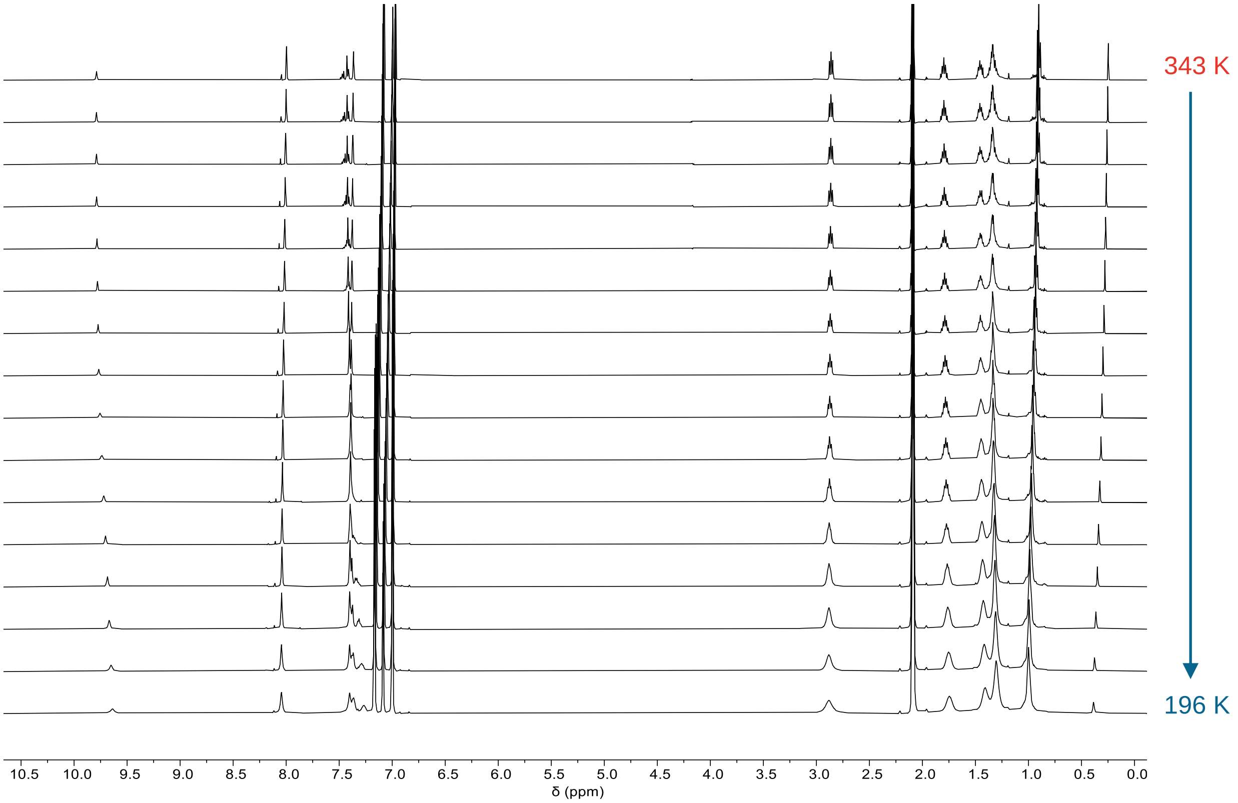

Supplementary Figure 29. Variable temperature ^{1}H NMR spectra of **2H-Car-C6** (500 MHz, 343 → 196 K each 10 K, toluene-d_8, 9.25 mM). Peak broadening is observed as the temperature is decreased.

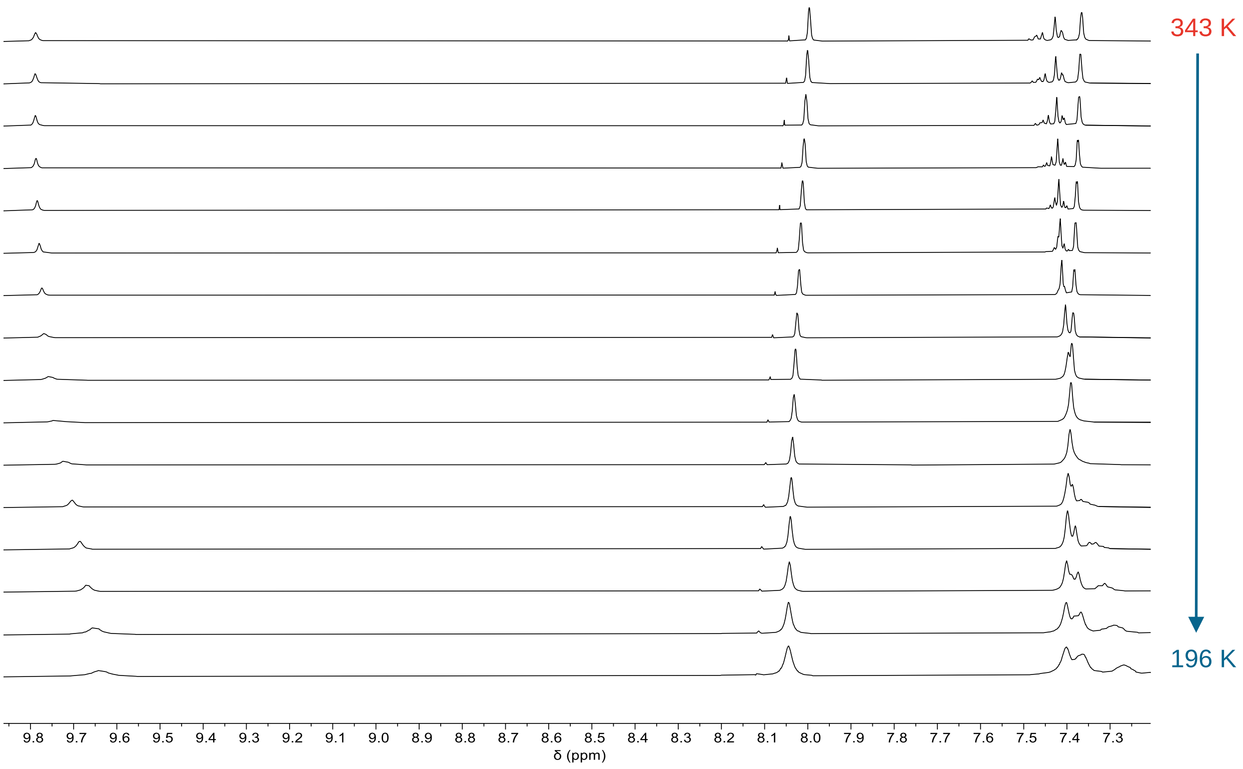

Supplementary Figure 30. Variable temperature ^{1}H NMR spectra of **2H-Car-C6** aromatic region (500 MHz, 343 → 196 K each 10 K, toluene-d_8, 9.25 mM). Peak broadening is observed as the temperature is decreased.

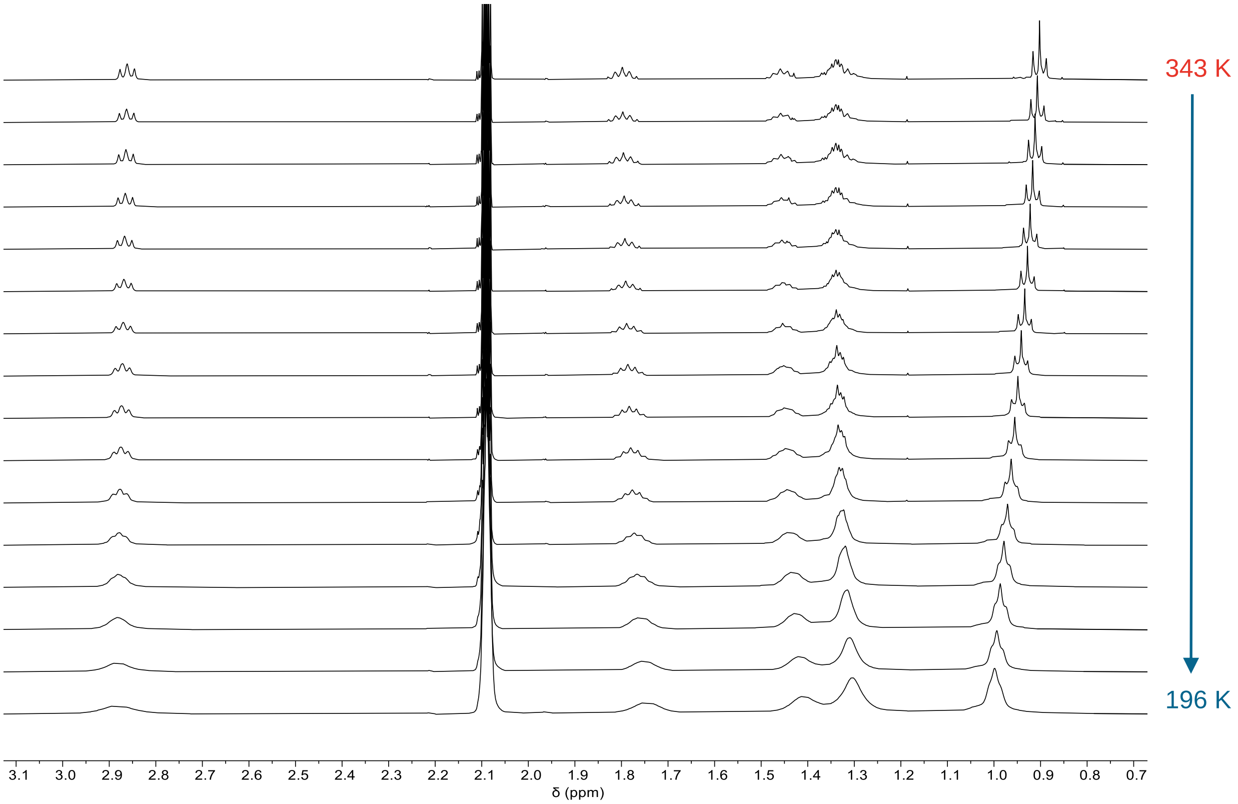

Supplementary Figure 31. Variable temperature ^1H NMR spectra of **2H-Car-C6** aliphatic region (500 MHz, 343 → 196 K each 10 K, toluene-d_8, 9.25 mM). Peak broadening is observed as the temperature is decreased.

Supplementary Table 2a. Summary of ^{1}H NMR (500 MHz, 1,1,2,2-tetrachloroethane-d_2, 7.55 mM) chemical shift variation of **2H-Car-C6** aromatic region with respect to temperature.

2H-Car-C6

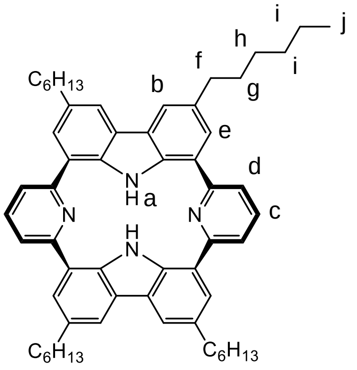

NMR shifts (center of signal) of **2H-Car-C6** aromatic region in 1,1,2,2-tetrachloroethane-d_2 (δ; Δδ[a]) in ppm

T / K	a	b	c	d	e
**343**	9.68; +0.04	8.02[b]; +0.03	8.00[c]; 0.00	7.69; +0.02	7.40[b]; +0.02
**333**	9.68	8.01[b]; +0.02	8.00[c]	7.68; +0.01	7.40[b]
**323**	9.67; +0.03	8.01[b]	8.00[c]	7.68	7.39[b]; +0.01
**313**	9.66; +0.02	8.00[b]; +0.01	8.00[c]	7.68	7.39[b]
**303**	9.65; +0.01	8.00[b]	8.00[c]	7.68	7.38[b]; 0.00
**293**	9.64; 0.00	7.99[b]; 0.00	8.00[c]	7.67; 0.00	7.38[b]
**283**	9.63; −0.01	7.99[b]	8.00[c]	7.67	7.37[b]; −0.01
**273**	9.63	7.99[b]	7.99[b c]; −0.01	7.67	7.37[b]
**263**	9.62; −0.02	7.98[b]; −0.01	7.99[b,c]	7.66[b]; −0.01	7.36[b]; −0.02
**253**	9.60; −0.04	7.98[b]	7.99[b,c]	7.66[b]	7.35[b]; −0.03
**243**	9.59; −0.05	7.97[b]; −0.02	7.99[b,c]	7.66[b]	7.35[b]
**233**	9.58[b]; **−0.06**	7.97[b]	7.99[b,c]	7.66[b]	7.34[b]; −0.04

[a] Δδ referenced to 293 K, [b] broad signal, [c] assignment unclear.

Supplementary Table 2b. Summary of ^{1}H NMR (500 MHz, 1,1,2,2-tetrachloroethane-d_2, 7.55 mM) chemical shift variation of **2H-Car-C6** aliphatic region with respect to temperature.

2H-Car-C6

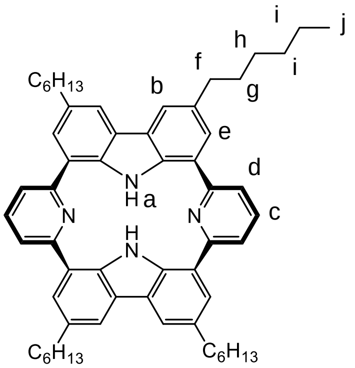

NMR shifts (center of signal) of **2H-Car-C6** aliphatic region in 1,1,2,2-tetrachloroethane-d_2 (δ; Δδ[a]) in ppm

T / K	f	g	h	i	j
**343**	2.90; +0.04	1.83; +0.07	1.50; **+0.06**	1.41; **+0.06**	0.96; +0.05
**333**	2.90	1.81; +0.05	1.49; +0.05	1.40; +0.05	0.95; +0.04
**323**	2.89; +0.03	1.80; +0.04	1.47; +0.03	1.39; +0.04	0.94; +0.03
**313**	2.88; +0.02	1.79; +0.03	1.46; +0.02	1.38; +0.03	0.93; +0.02
**303**	2.87; +0.02	1.78; +0.02	1.45; +0.01	1.37; +0.02	0.92; +0.01
**293**	2.86[b]; 0.00	1.76[b]; 0.00	1.44[b]; 0.00	1.35[b]; 0.00	0.91[b]; 0.00
**283**	2.85[b]; −0.01	1.75[b]; −0.01	1.42[b]; −0.02	1.34[b]; −0.01	0.90[b]; −0.01
**273**	2.83[b]; −0.03	1.73[b]; −0.03	1.41[b]; −0.03	1.33[b]; −0.02	0.89[b]; −0.02
**263**	2.82[b]; −0.04	1.72[b]; −0.04	1.40[b]; −0.04	1.31[b]; −0.04	0.88[b]; −0.03
**253**	2.81[b]; −0.05	1.70[b]; **−0.06**	1.38[b]; **−0.06**	1.30[b]; −0.05	0.87[b]; −0.04
**243**	2.80[b]; **−0.06**	1.68[b]; **−0.08**	1.36[b]; **−0.08**	1.28[b]; **−0.07**	0.85[b]; **−0.06**
**233**	2.79[b]; **−0.07**	1.66[b]; **−0.010**	1.35[b]; **−0.09**	1.26[b]; **−0.09**	0.84[b]; **−0.07**

[a] Δδ referenced to 293 K, [b] broad signal, [c] assignment unclear.

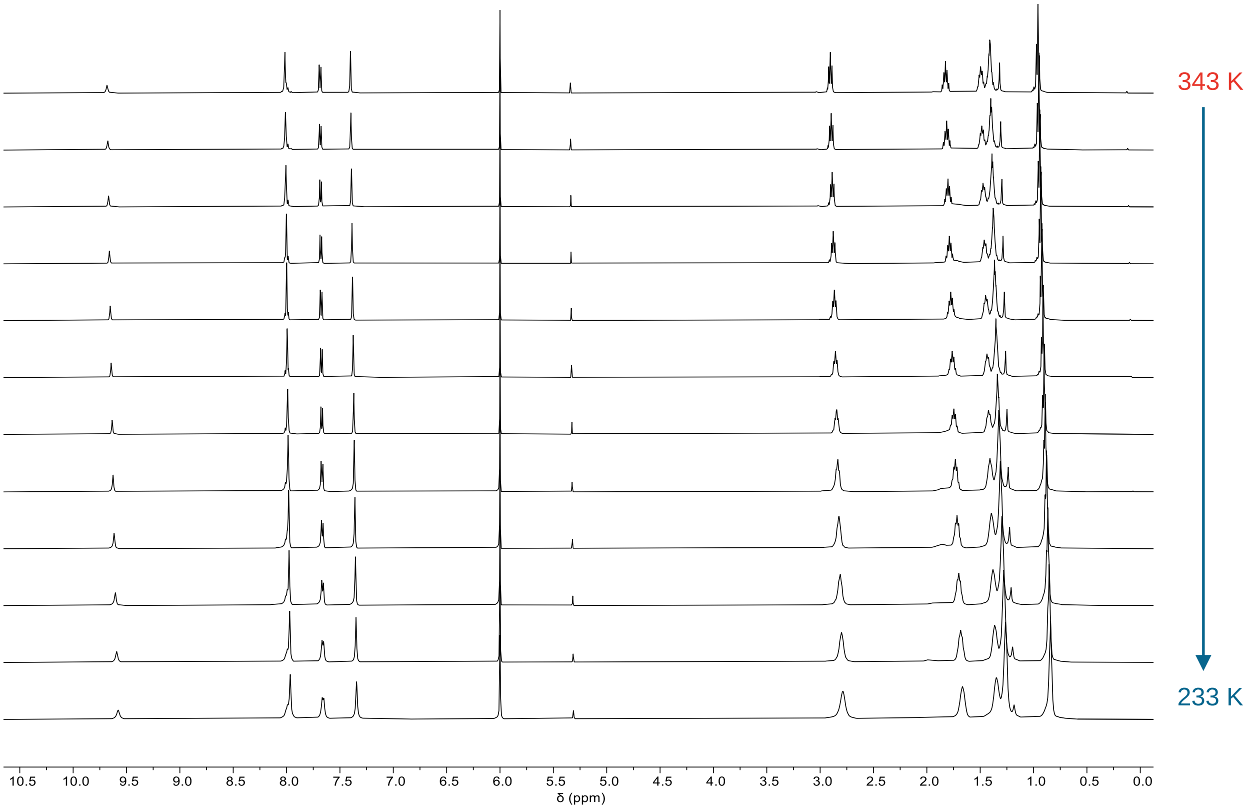

Supplementary Figure 32. Variable temperature ^{1}H NMR spectra of **2H-Car-C6** (500 MHz, 343 → 233 K each 10 K, 1,1,2,2-tetrachloroethane-d_2, 7.55 mM). Peak broadening is observed as the temperature is decreased.

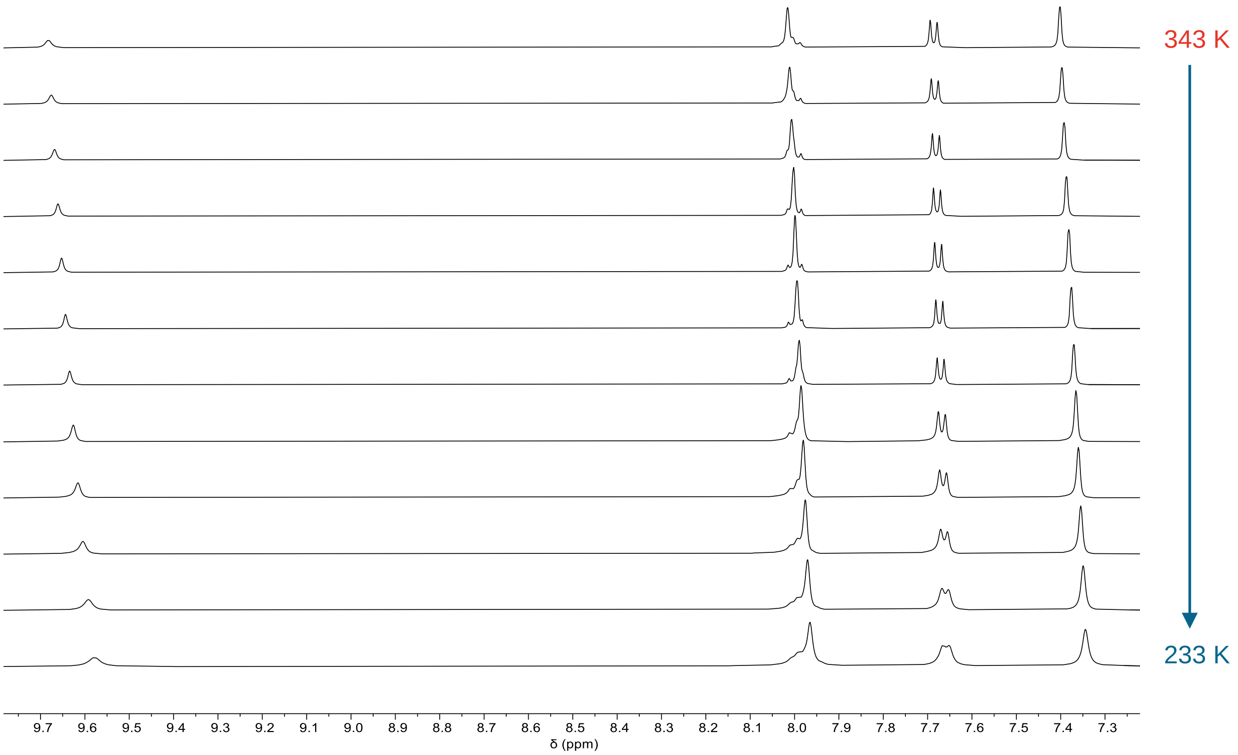

Supplementary Figure 33. Variable temperature ^{1}H NMR spectra of **2H-Car-C6** aromatic region (500 MHz, 343 → 233 K each 10 K, 1,1,2,2-tetrachloroethane-d_2, 7.55 mM). Peak broadening is observed as the temperature is decreased.

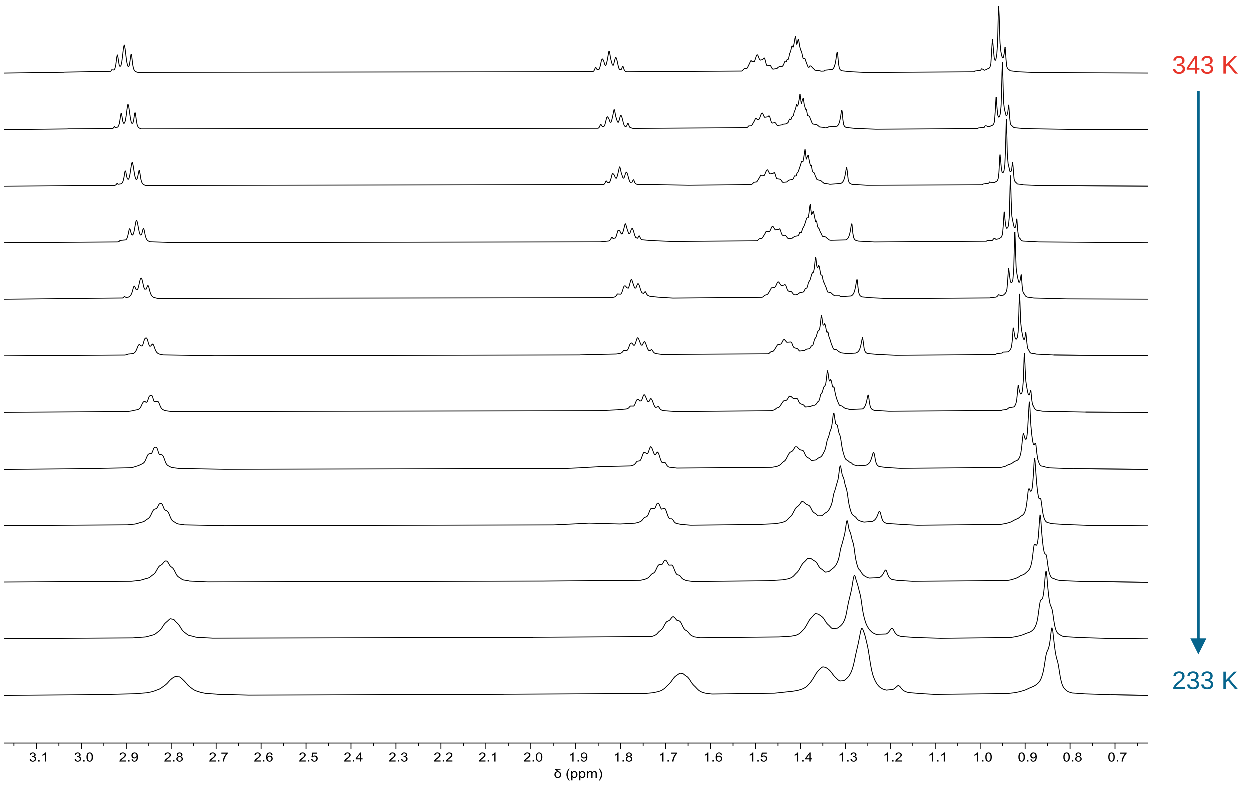

Supplementary Figure 34. Variable temperature ^{1}H NMR spectra of **2H-Car-C6** aliphatic region (500 MHz, 343 → 233 K each 10 K, 1,1,2,2-tetrachloroethane-d_2, 7.55 mM). Peak broadening is observed as the temperature is decreased.

Variable Concentration ^{1}H NMR Spectra

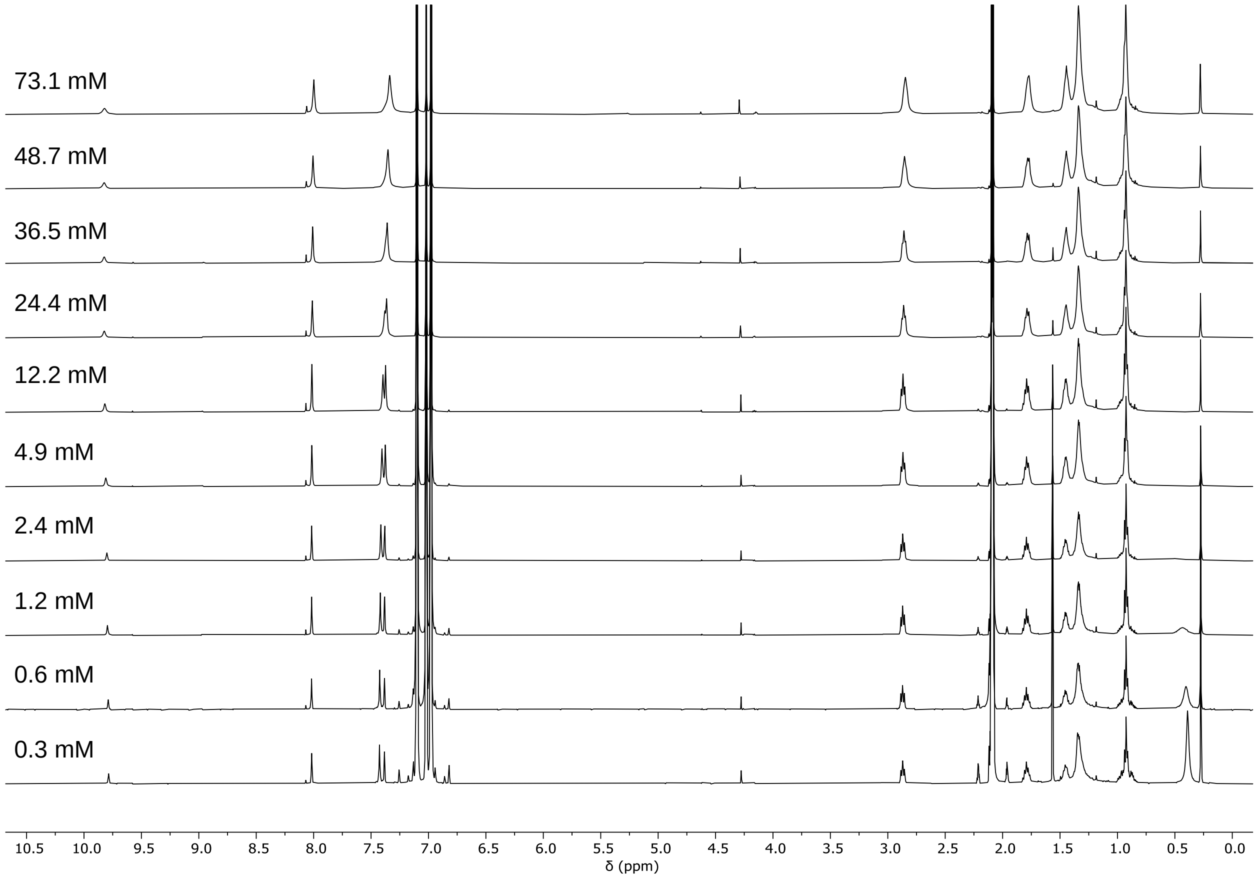

Supplementary Figure 35. Variable concentration ^{1}H NMR spectra of **2H-Car-C6** (500 MHz, 73.1 → 0.3 mM, toluene-d_8, 298 K). Peak broadening is observed as the concentration is increased.

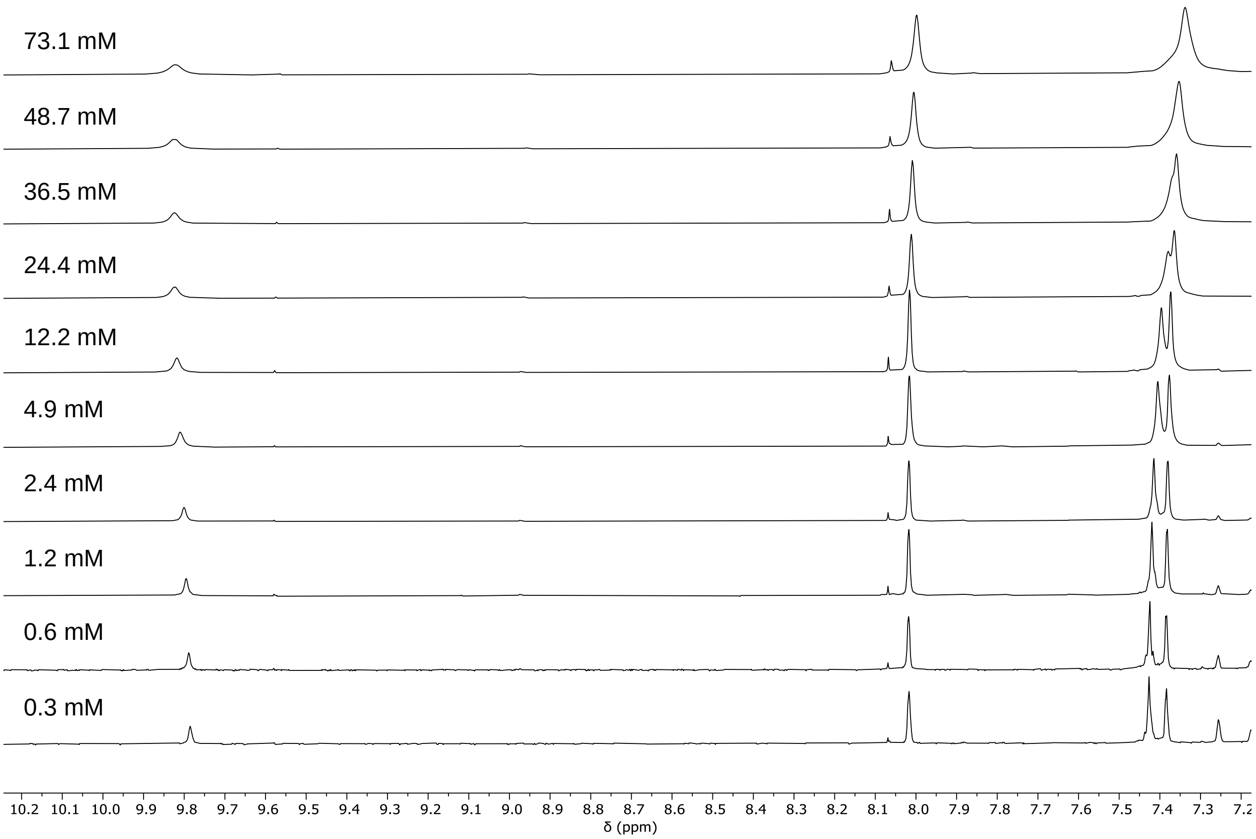

Supplementary Figure 36. Variable concentration ^{1}H NMR spectra of **2H-Car-C6** aromatic region (500 MHz, 73.1 → 0.3 mM, toluene-d_8, 298 K). Peak broadening is observed as the concentration is increased and pyridine signals only resolve at low concentrations.

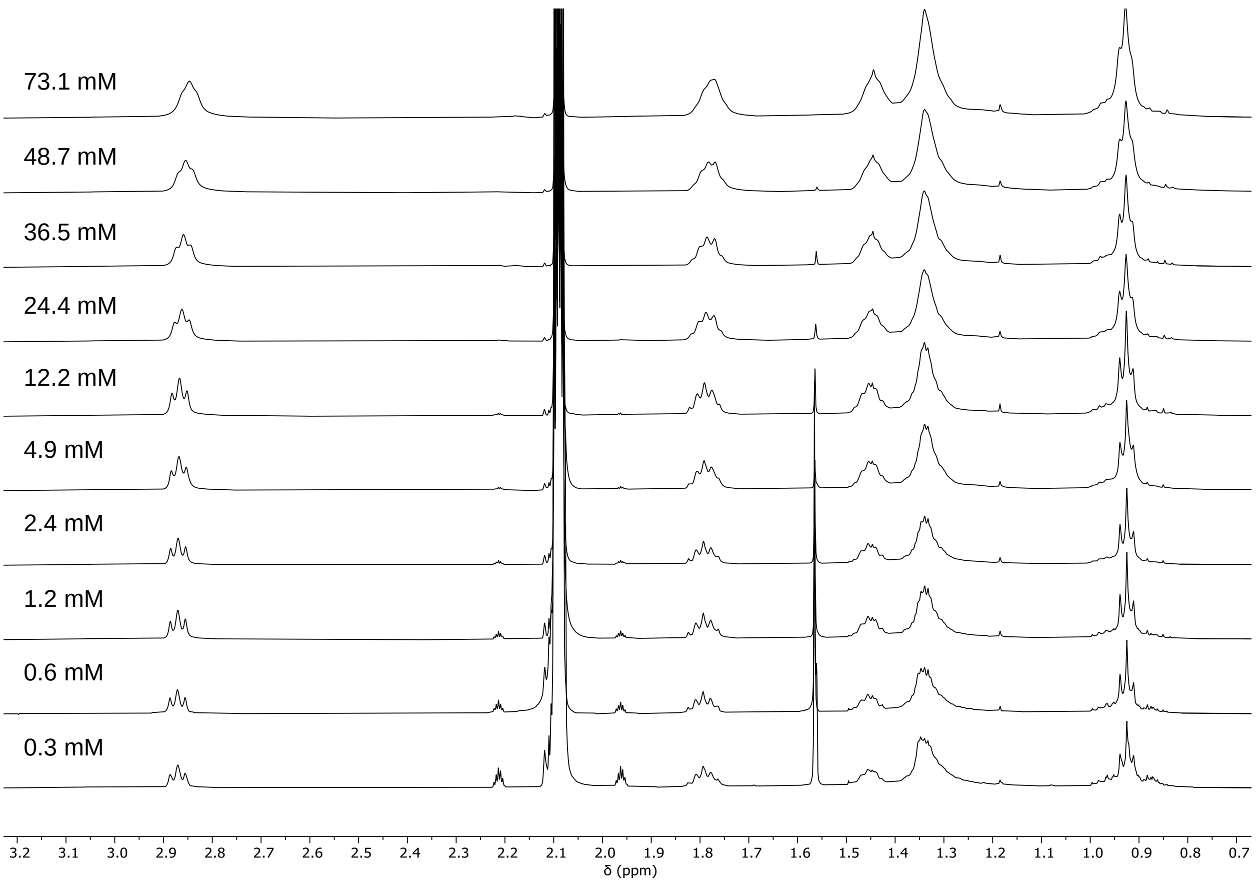

Supplementary Figure 37. Variable concentration ^{1}H NMR spectra of **2H-Car-C6** aliphatic region (500 MHz, 73.1 → 0.3 mM, toluene-d_8, 298 K). Peak broadening is observed as the concentration is increased.

^{1}H DOSY Spectra

Supplementary Table 3. Summary of DOSY (500 MHz, toluene-d_8, 4.88 mM) diffusion coefficient variation and apparent MWs of **2H-Car-C6** with respect to temperature.

DOSY diffusion coefficient and apparent MW variation of **2H-Car-C6** in toluene-d_8 with temperature

T / K	D / m^2 s^{-1}	Apparent MW[a] / g mol^{-1}	Ratios[b]
**343**	1.06×10^{-9}	1274.8	1.6 / 1.0
**298**	5.48×10^{-10}	1464.4	1.8 / 1.1
**253**	2.49×10^{-10}	1380.5	1.7 / 1.1
**233**	1.46×10^{-10}	1559.5	1.9 / 1.2
**196**	3.16×10^{-11}	4293.6	5.2 / 3.4

[a] Evans R. et al. Quantitative interpretation of diffusion-ordered NMR spectra: can we rationalize small molecule diffusion coefficients? *Angew. Chem. Int. Ed*. **52**, 3199–3202 (2013).
[b] Ratio to $MW_{2H\text{-}Car\text{-}C6}$ = 821.2 g mol^{-1} / Ratio to apparent MW_{min} in toluene-d_8.

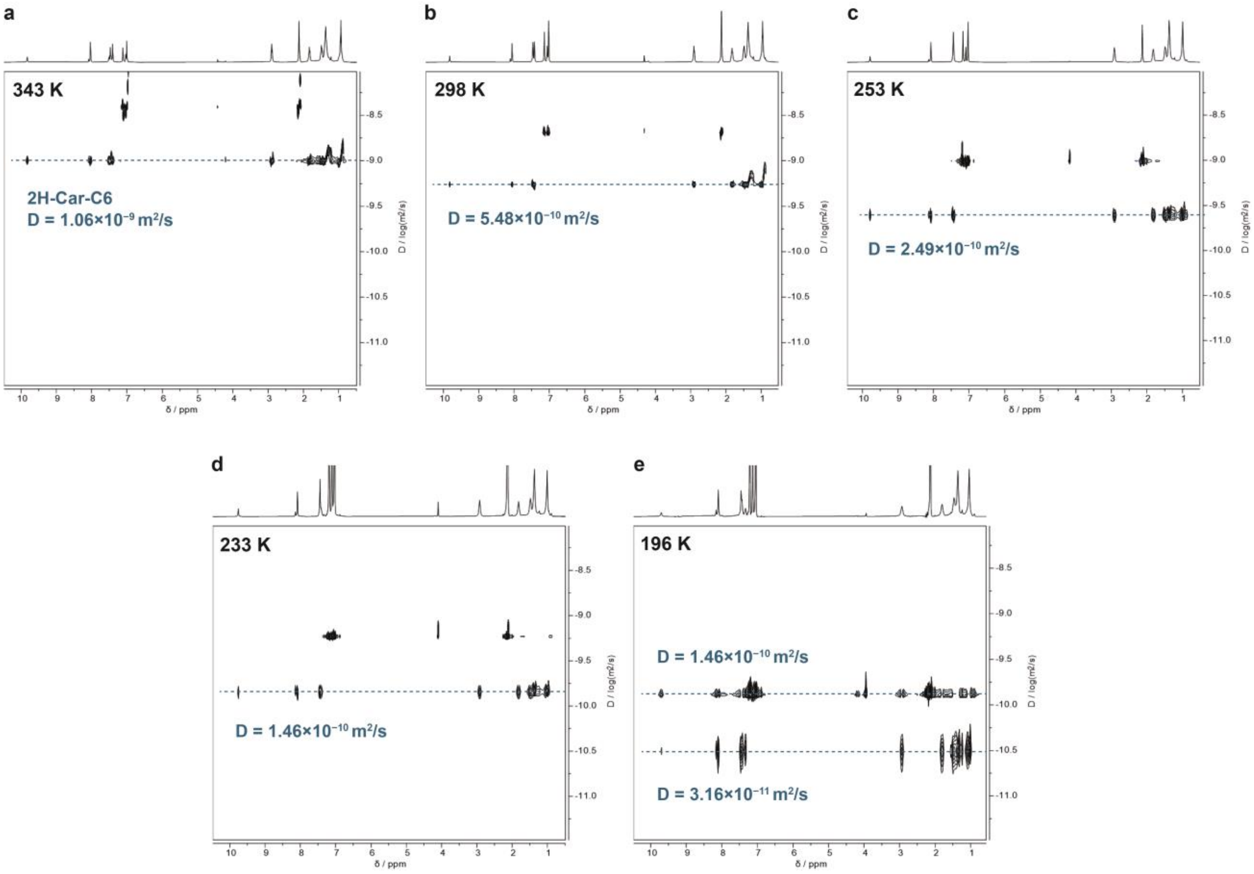

Supplementary Figure 38. Variable temperature DOSY spectra of **2H-Car-C6** (500 MHz, toluene-d_8, 4.88 mM) at **a**, 343 K; **b**, 298 K; **c**, 253 K; **d**, 233 K and **e**, 196 K.

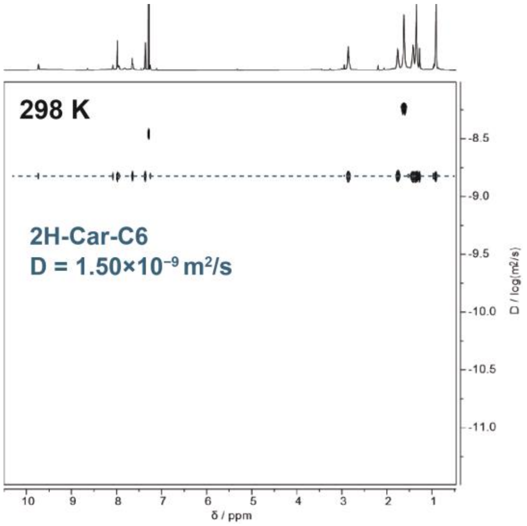

DOSY diffusion coefficient and apparent MW of **2H-Car-C6** at **298 K**

Solvent	D / m^2 s^{-1}	Apparent MW[a] / g mol^{-1}
**$CDCl_3$**	1.50×10^{-9}	184.7
**toluene-d_8**	5.48×10^{-10}	1464.4

[a] Evans R. et al. Quantitative interpretation of diffusion-ordered NMR spectra: can we rationalize small molecule diffusion coefficients? *Angew. Chem. Int. Ed*. **52**, 3199–3202 (2013).

Supplementary Figure 39. Left, DOSY spectrum of **2H-Car-C6** (600 MHz, 298 K, $CDCl_3$, 1.95 mM). **Right**, summary of obtained DOSY diffusion coefficient and apparent MW of **2H-Car-C6**, comparing with **2H-Car-C6** in toluene-d_8 (500 MHz, 298 K, 4.88 mM).

Spectroscopic Data

Supplementary Table 4. Summary of UV-vis absorption maxima with corresponding logarithmic extinction coefficients, excitation wavelengths, fluorescence emission maxima and associated quantum yields. All measurements were made in toluene at 298 K.

	$\lambda_{abs,max}$ / nm	log ε^a	λ_{ex} / nm	$\lambda_{em,max}$ / nm	Φ_f^b / %
**2H-Car-C6**	314, 376	4.46, 4.17	314	390	40.5
**2H-Car-C8**	314, 376	4.65, 4.32	314	390	47.3
**2H-Car-C12**	314, 375	4.92, 4.55	314	390	36.3
**2H-Car-*t*Bu**	313, 372	4.59, 4.29	313	387	26.3
**2H-Car-H**	304, 363	4.21, 3.84	304	377	34.7

[a] ε given in $M^{-1}cm^{-1}$, [b] integrating sphere.

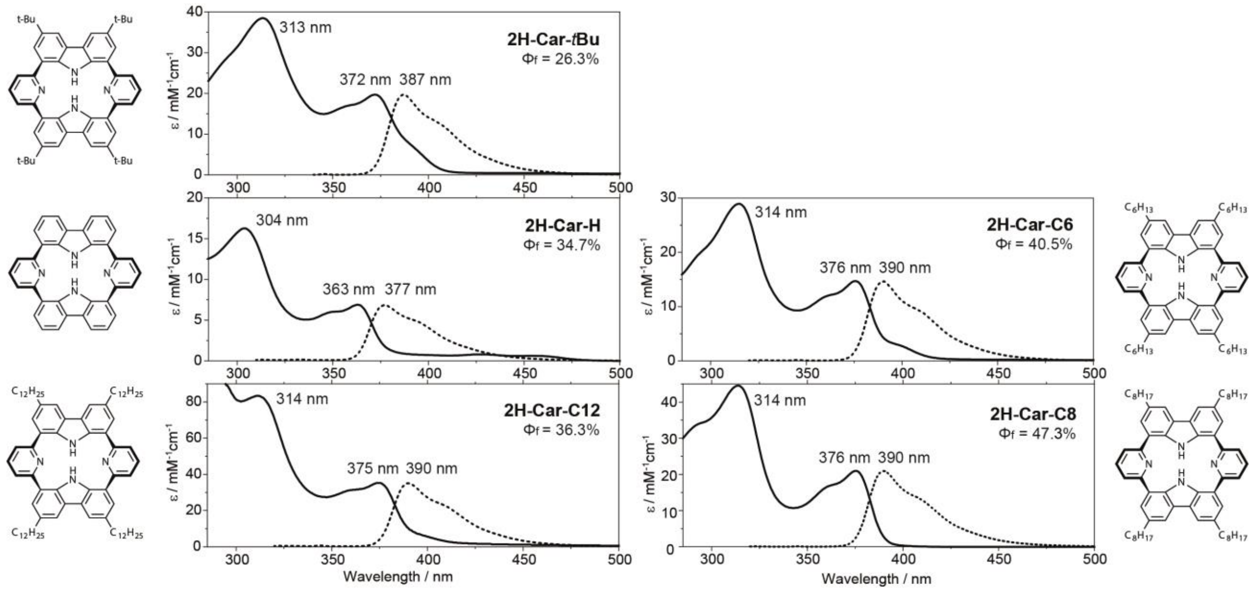

Supplementary Figure 40. Steady-state UV-vis absorption (continuous lines) and fluorescence emission (dashed lines) spectra of indicated carpyridines. All measurements were made in toluene at 298 K.

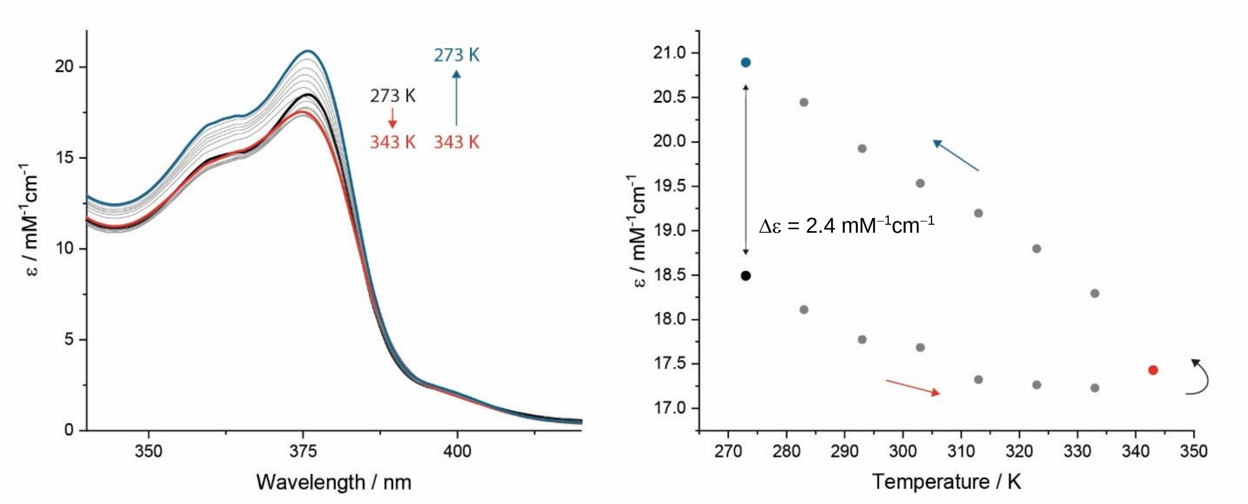

Supplementary Figure 41. VT UV-vis spectra of a 1 mM solution of **2H-Car-C6** in toluene, showing how the extinction coefficient goes down when increasing the temperature and how it goes back up, above the starting point (+ 2.4 $mM^{-1}cm^{-1}$), when lowering the temperature.

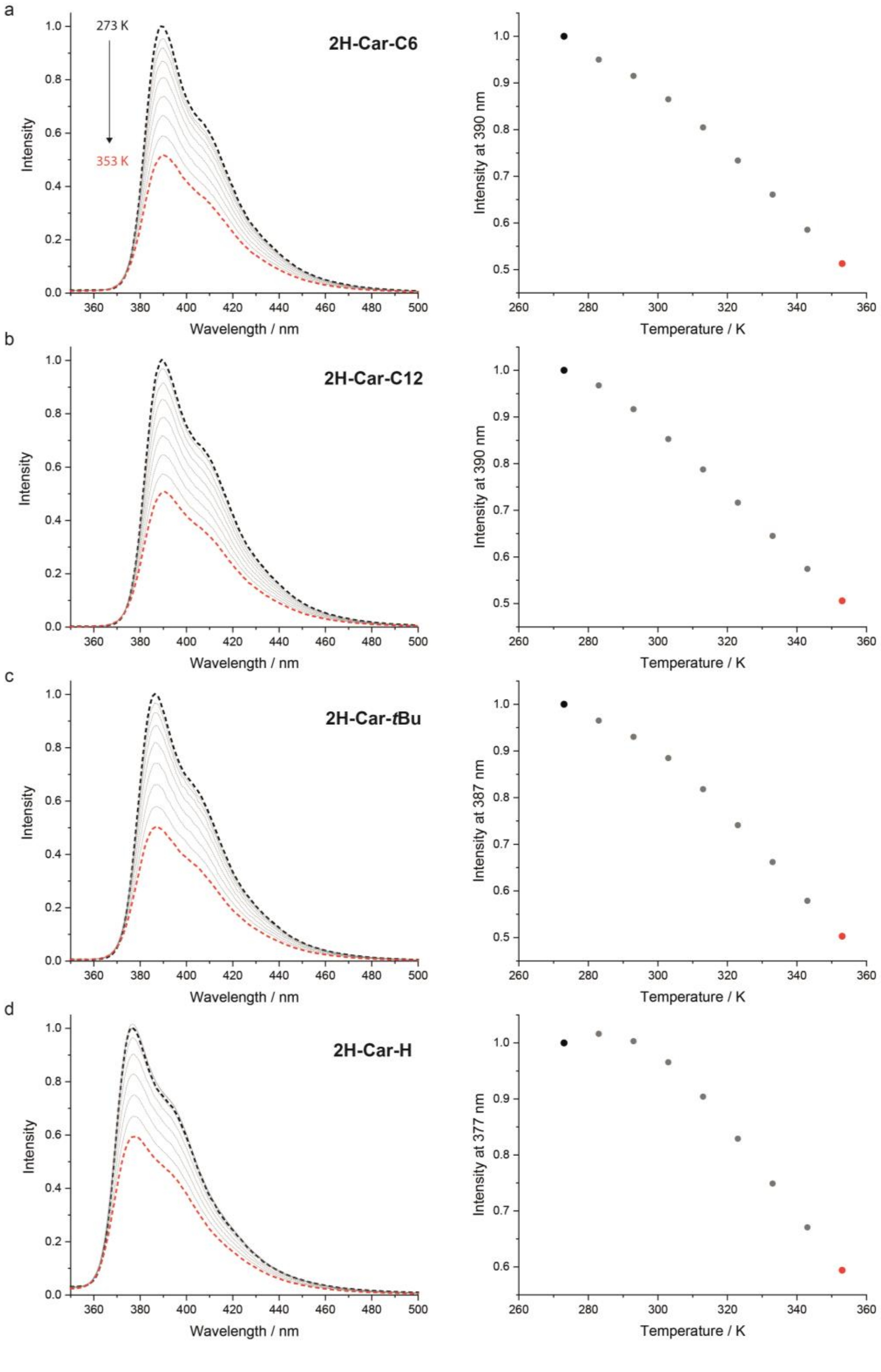

Supplementary Figure 42. VT Fluorescence spectra of **a**, **2H-Car-C6**; **b**, **2H-Car-C12**; **c**, **2H-Car-*t*Bu** and **d**, **2H-Car-H** in toluene, showing how the emission intensity at the maximum goes down when increasing the temperature. No significant changes are observed for any of the systems.

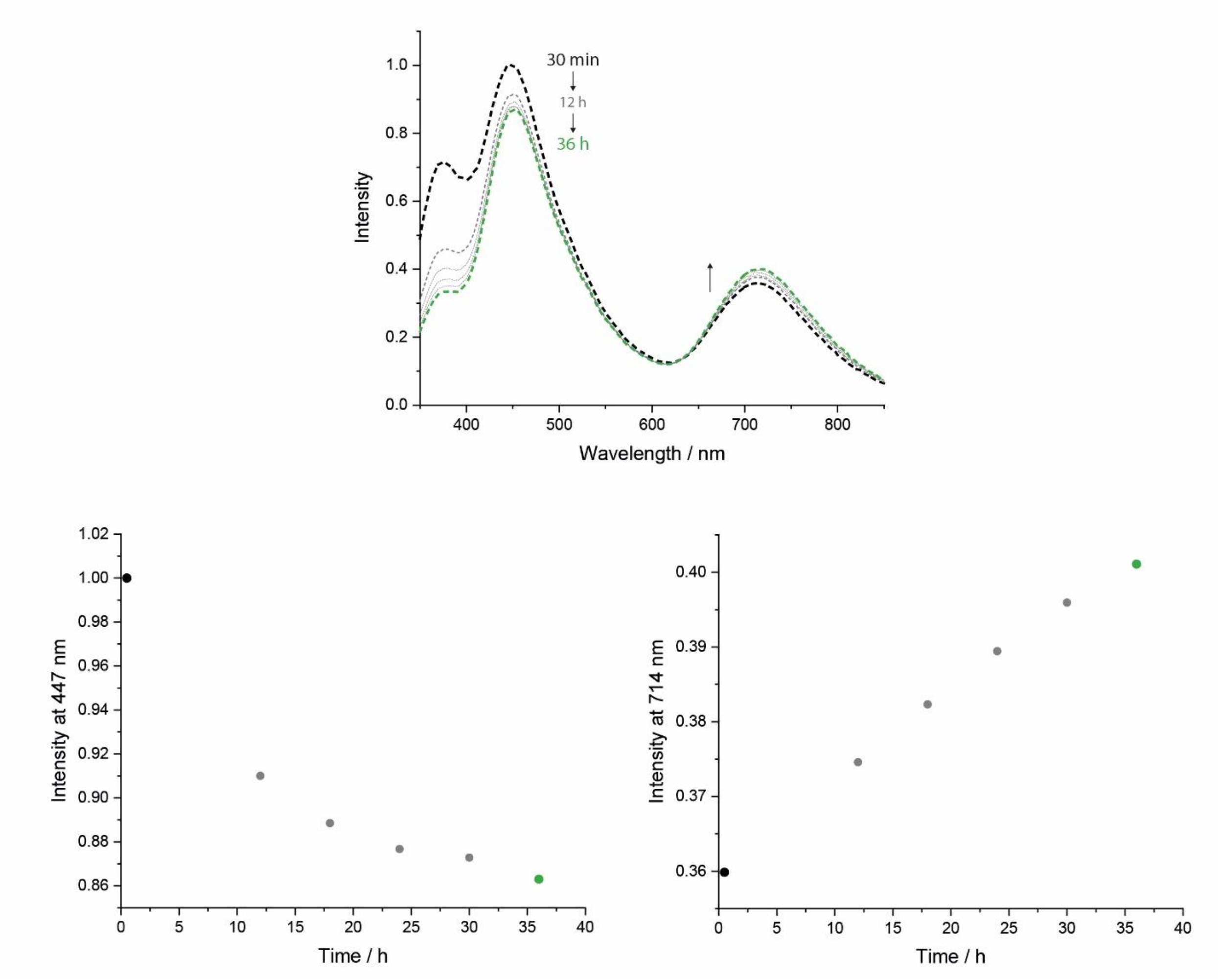

Supplementary Figure 43. Solid-state fluorescence spectra of **2H-Car-C6** over time. Sample was prepared from a 1 mM solution in toluene and evaporated. The peak at 447 nm shows an exponential decrease in intensity while the maxima at 714 nm increases linearly in intensity.

Microscopy

Additional TEM, SEM and STEM images

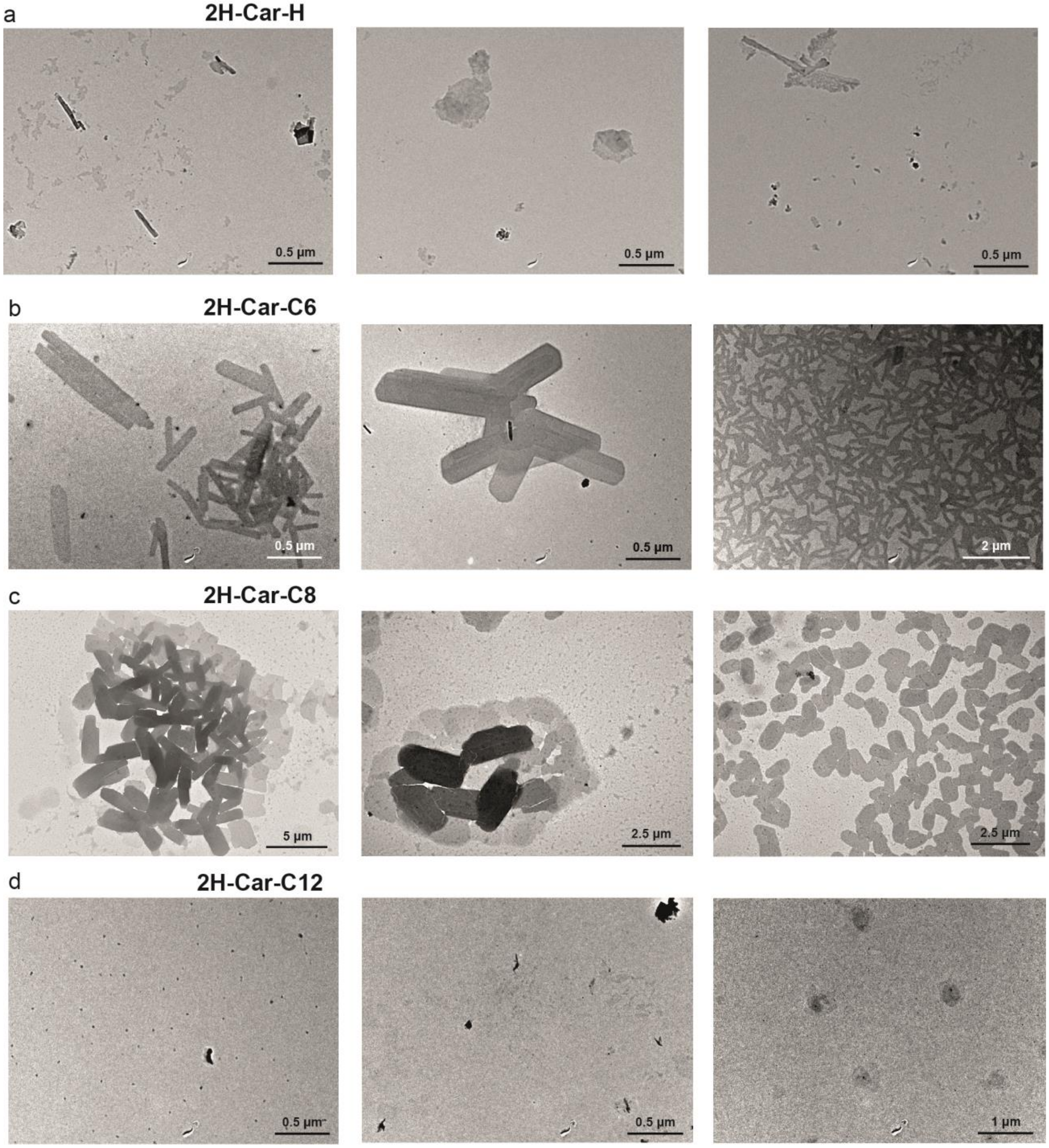

Supplementary Figure 44. TEM images of **a**, **2H-Car-H**; **b**, **2H-Car-C6**; **c**, **2H-Car-C8** and **d**, **2H-Car-C12** from a 1 mM solution in toluene. Micrometer length sheets are observed when **2H-Car-C6** and **2H-Car-C8** are evaporated from toluene but **2H-Car-C12** and **2H-Car-H** do not form ordered assemblies at all or consistently.

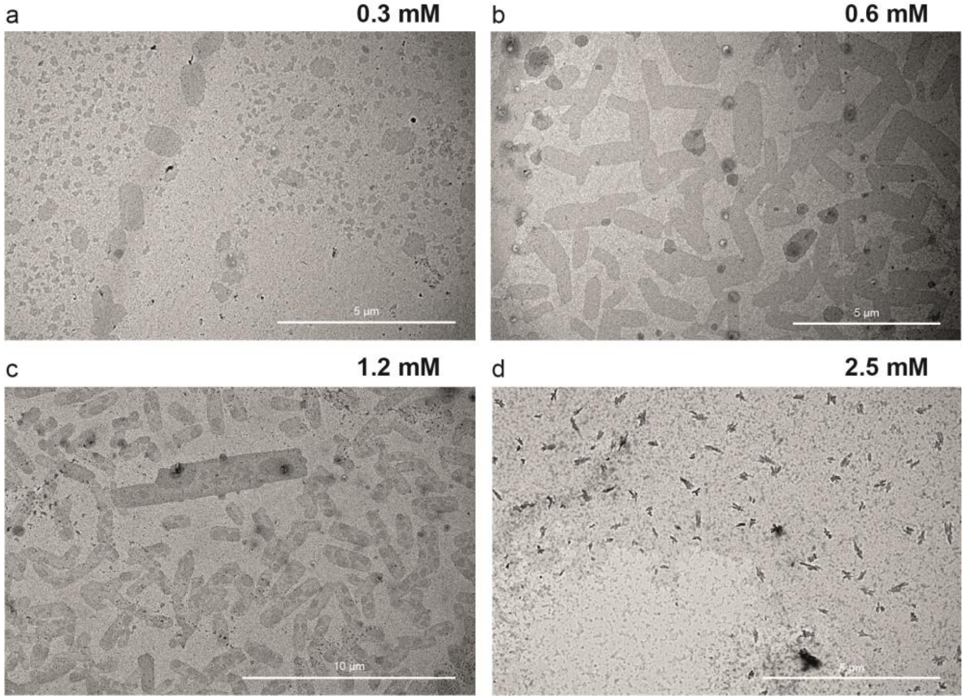

Supplementary Figure 45. Variable concentration TEM images of **2H-Car-C6** from toluene solutions with concentrations of **a**, 0.3 mM; **b**, 0.6 mM, **c**, 1.2 mM and **d**, 2.5 mM. Defined and consistent sheet formation is observed when working in a range of concentration of 0.6–1.2 mM.

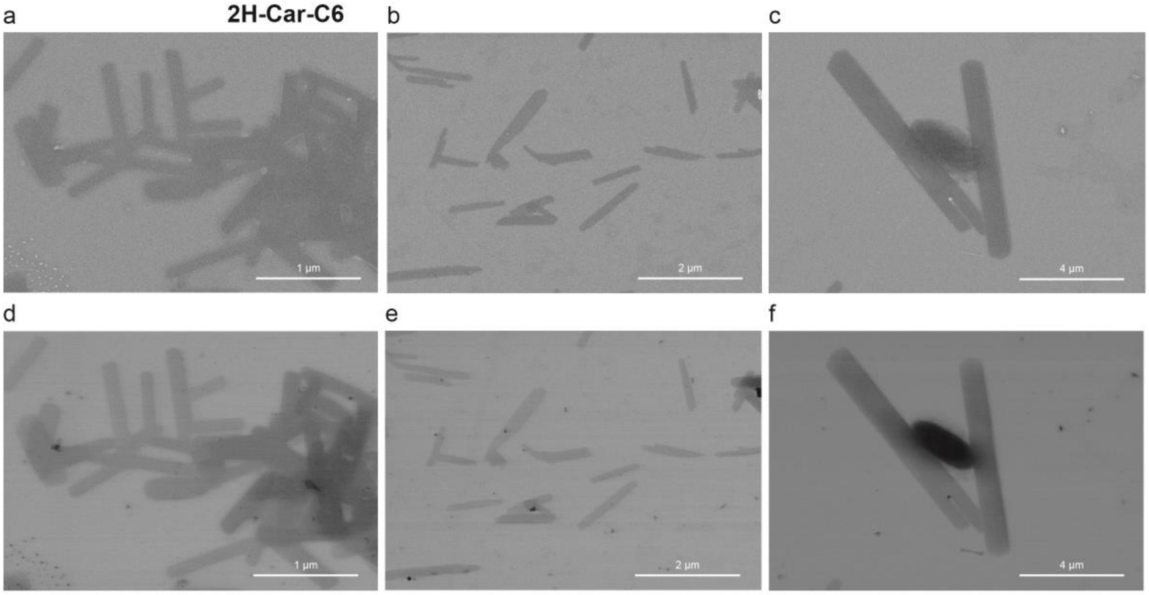

Supplementary Figure 46. a–c show SEM images and **d–f** show STEM images of **2H-Car-C6** from a 1 mM solution in toluene.

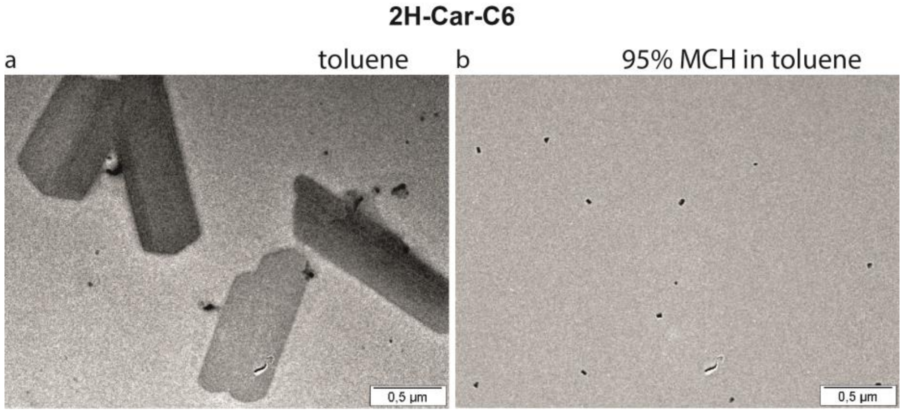

Supplementary Figure 47. TEM images of **2H-Car-C6** from a solution in **a**, toluene and **b**, 95% methylcyclohexane in toluene. 2D sheets are only observed in the case of toluene.

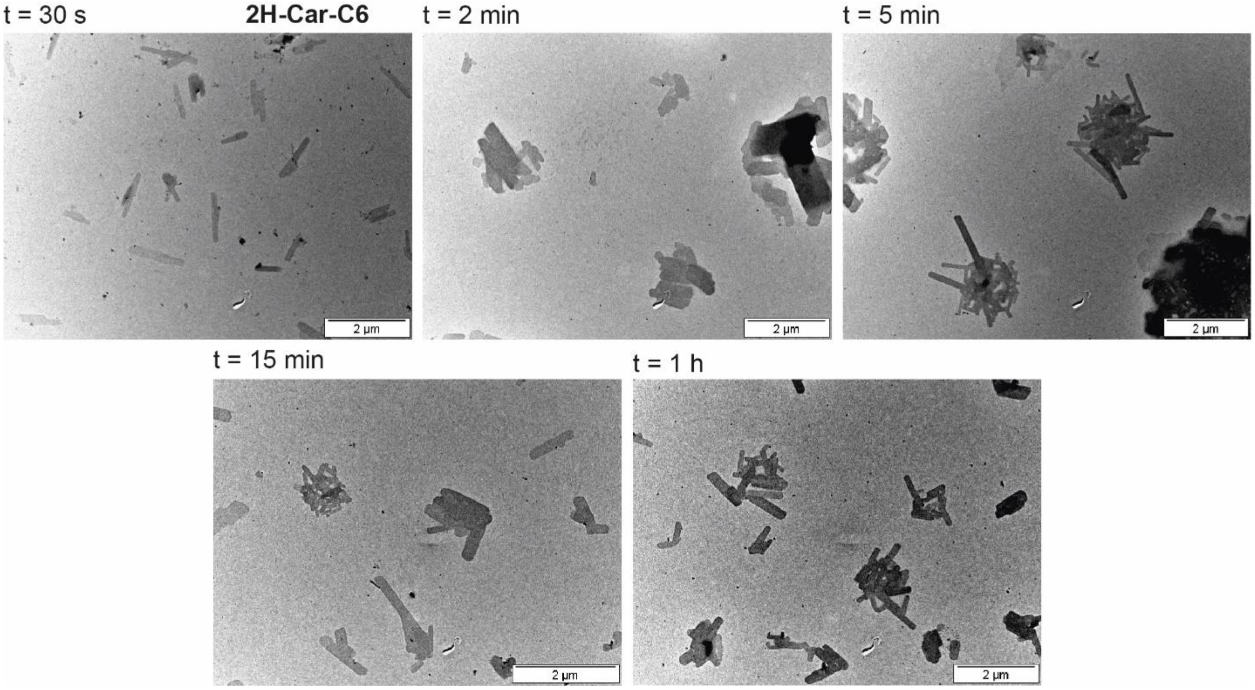

Supplementary Figure 48. Time dependent TEM images of **2H-Car-C6** from a 1 mM toluene solution. Formation of 2D sheets occurs after 30 seconds and there is little variation of sheet dimensions over time.

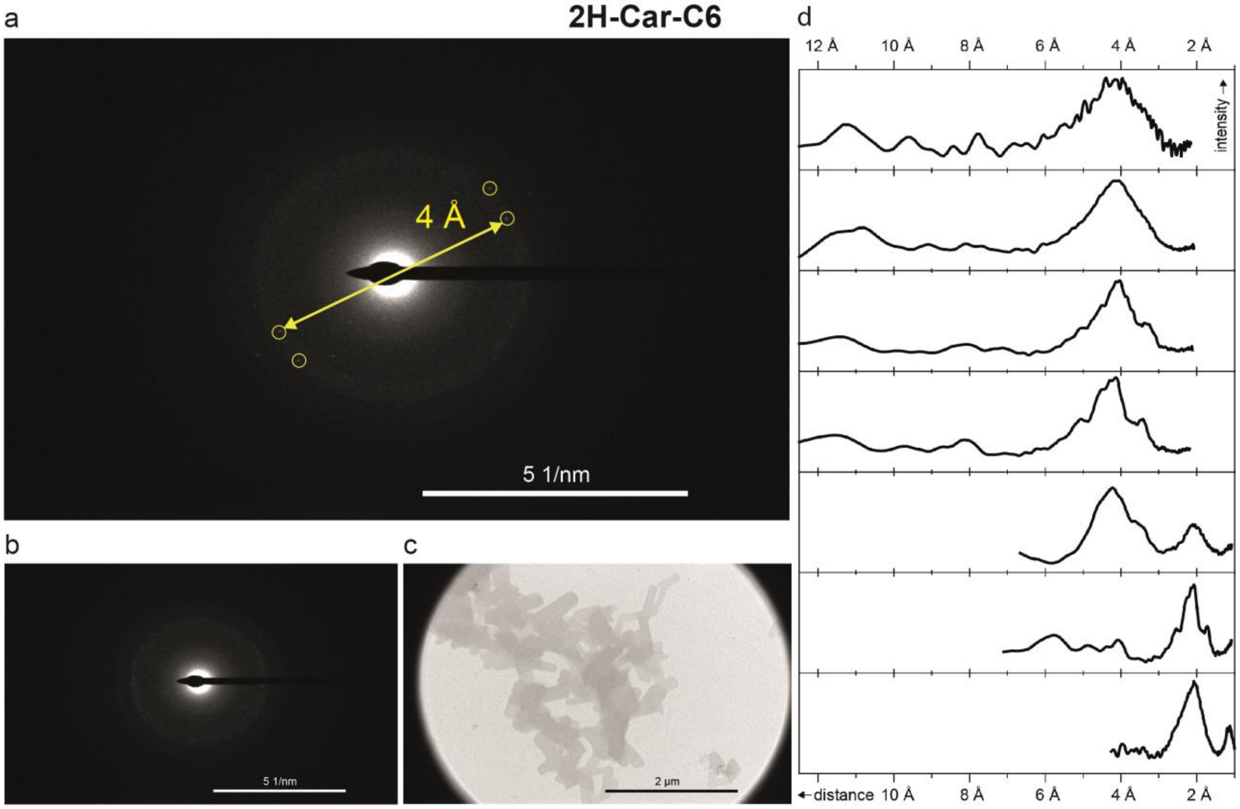

Supplementary Figure 49. Selected area electron diffraction (SAED) of **2H-Car-C6** from a 1 mM toluene solution. **a**, Original electron diffraction pattern of an ensemble of 2D-sheets which is presented in the manuscript as negative image. We consistently observe a diffracting halo as well as individual diffraction spots corresponding to individual sheets. **b**, Selected area electron diffraction of the region shown in **c**. **d**, Radial intensity analysis (sum) of all measured SAED patterns. As for **a**, the main feature is the halo of diffraction, as well as several clearly visible diffraction spots accounting for the high long-range order, while minor variations in characteristic distances are a strong indication of the soft-matter nature of these materials. Longer irradiation times (above 10 s) result in the weakening/disappearance of the diffraction patterns as expected for organic materials.

Additional AFM images

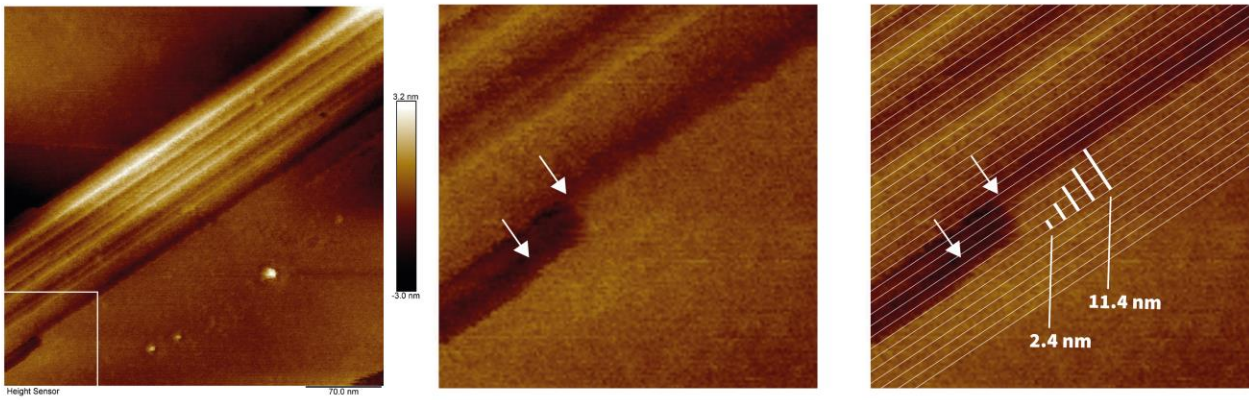

Supplementary Figure 50. Additional AFM images in which individual columnar assemblies and their propagation direction can be distinguished in regions with defects, and by the step thickness of the edges.

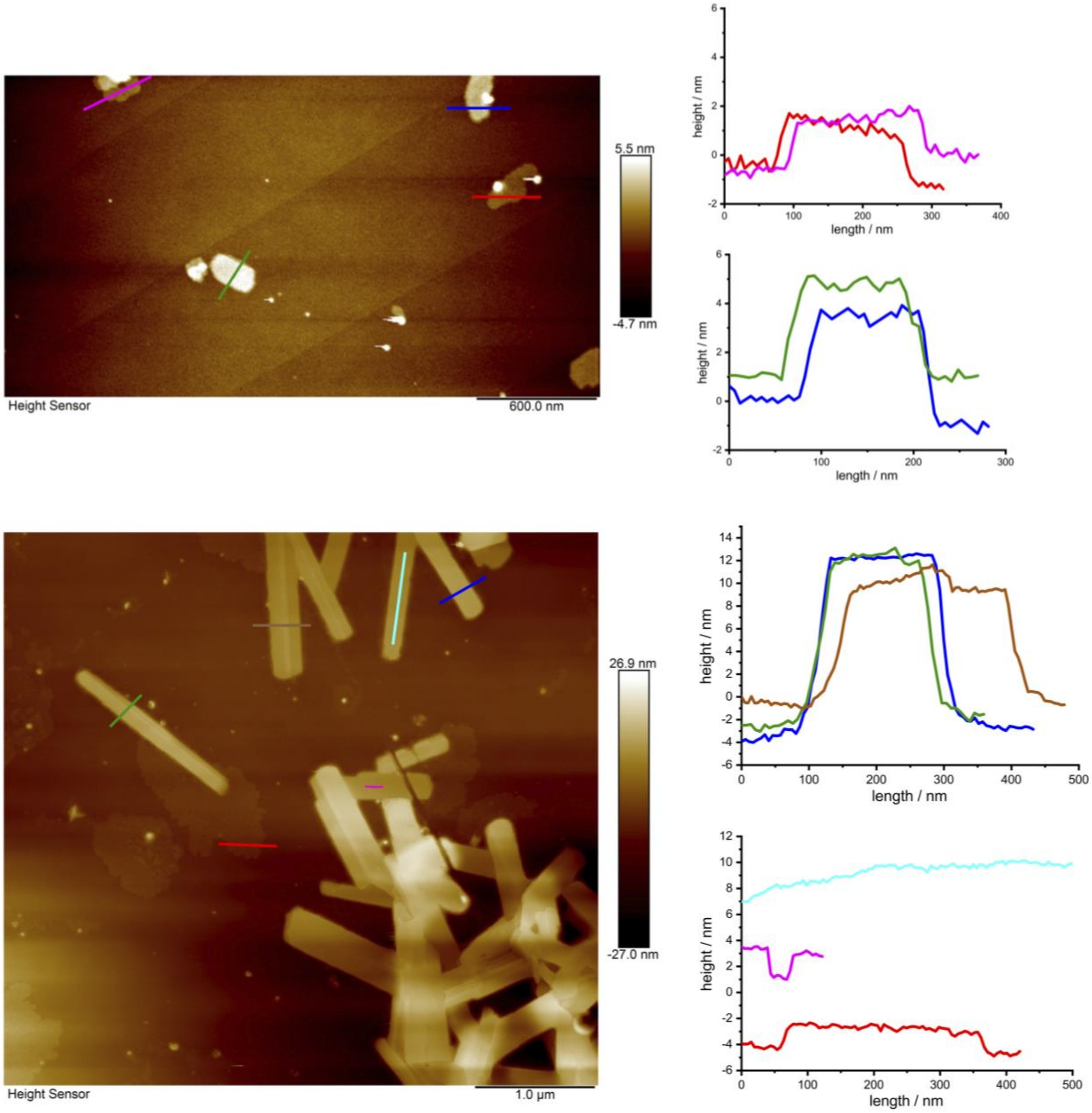

Supplementary Figure 51. Additional AFM measurements of **2H-Car-C6** from a 1 mM toluene solution. **Top**, Single and double layer 2D sheets. **Bottom**, Multi-layer 2D sheets.

Dynamic Light Scattering

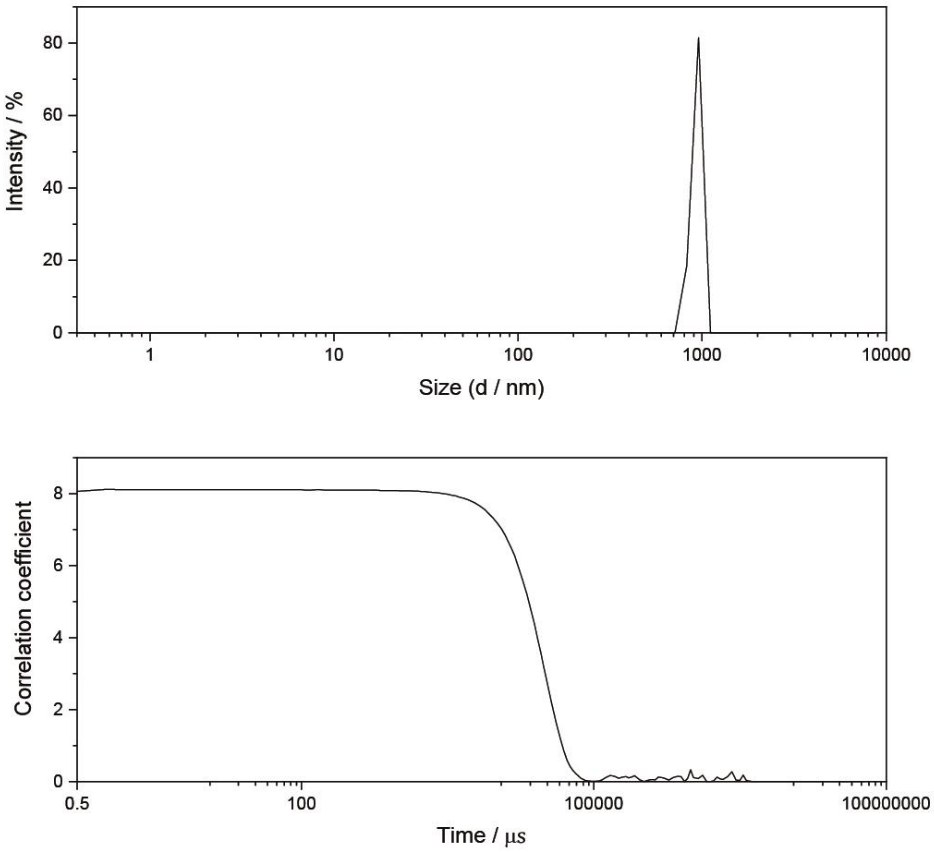

Supplementary Figure 52. DLS size distribution of a 10^{-3} M solution of **2H-Car-C6** in toluene at 30 °C showing the presence of large aggregates (1 μm), and its correlation coefficient plot.

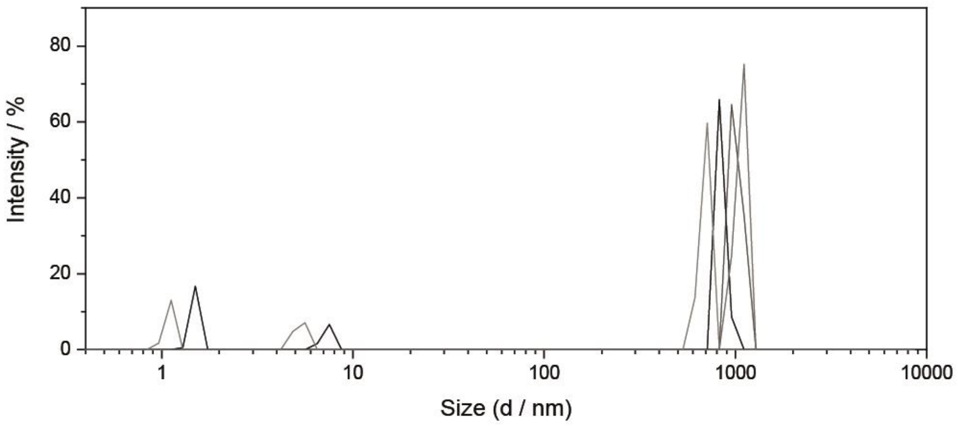

Supplementary Figure 53. DLS size distribution of a 10^{-3} M solution of **2H-Car-C6** in toluene at 10 °C (several runs) showing the presence of large aggregates (up to 1 μm) as well as smaller size particles corresponding to small clusters (1–10 nm) also observed by electron microscopy.

Differential Scanning Calorimetry

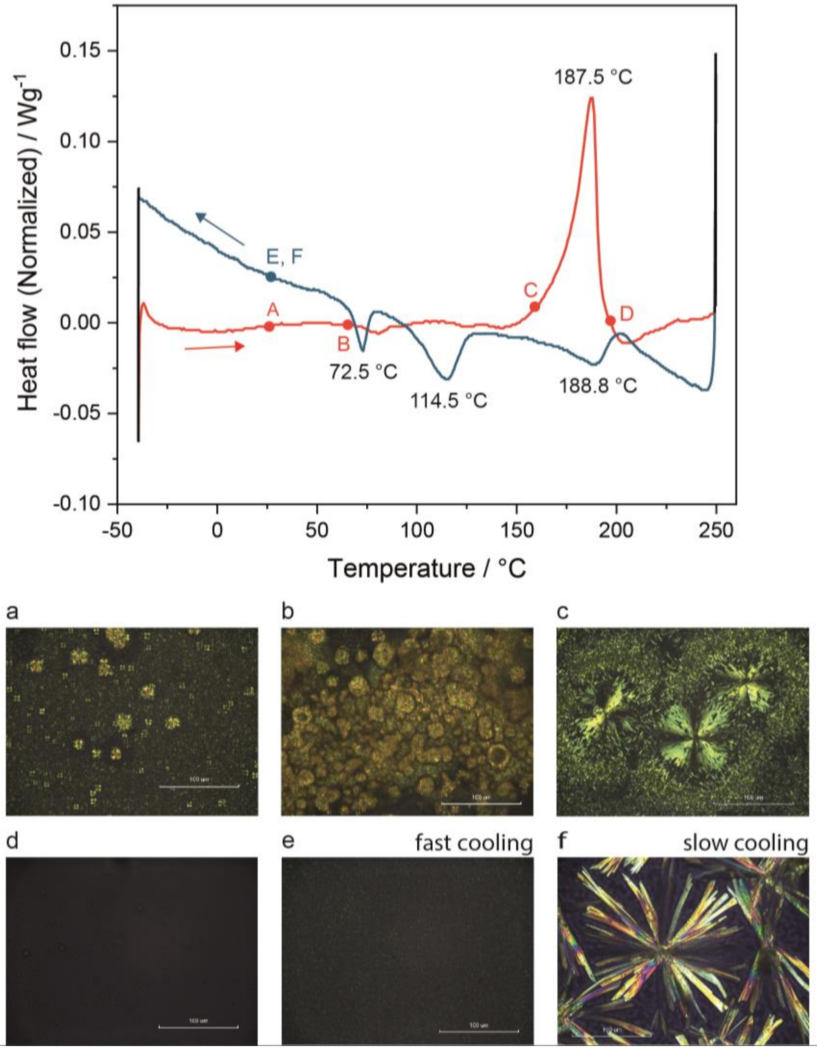

Supplementary Figure 54. DSC curve of **2H-Car-C6**. Heating up (red arrow) shows the melting point at 187.5 °C, while cooling down (blue arrow) shows how the sample undergoes several crystallization processes (188.8, 114.5 and 72.5 °C). **a–c**, Polarized optical microscopy images show the change in morphology of **2H-Car-C6** at different temperature ranges, with the occurrence of a liquid-crystal like behavior (i.e., alignment with sheering) around 100 °C; **d**, shows melting of the sample; **e**, shows the new morphology of the sample after fast cooling down to room temperature and **f**, shows the new morphology of the sample after slow cooling down to room temperature.

Infrared Spectroscopy

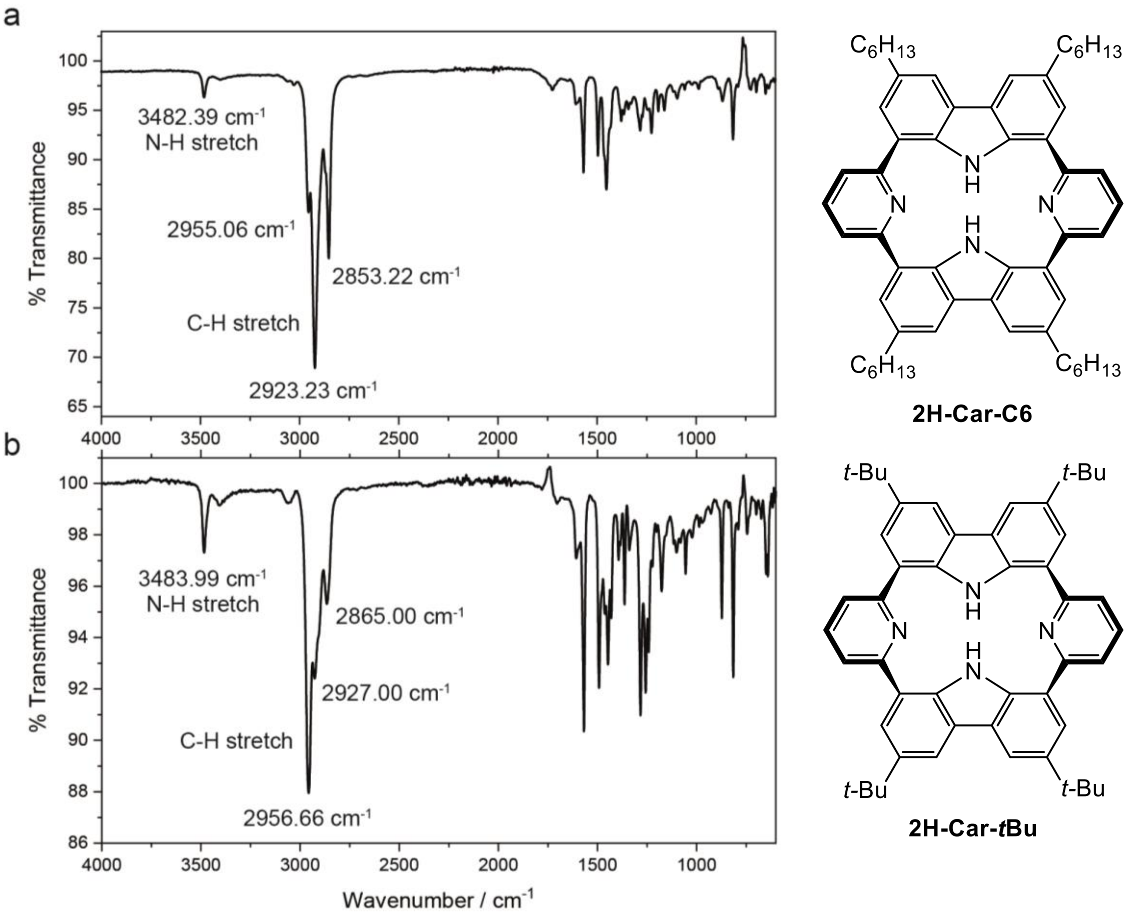

Supplementary Figure 55. IR spectra of **a**, **2H-Car-C6** and **b**, **2H-Car-*t*Bu** measured in thin film. The main stretching peaks are shown, with particular interest in the N-H stretching at around 3480 cm^{-1}, that appears to be similar in both cases.

DFT and semi-empirical calculations

Methods

All models were built using GaussView 6 and the corresponding optimizations carried out using Gaussian 16 revision C.01[S15]. For DFT calculations, the choice of functional was based on various examples from the literature. To avoid functional-specific errors, we decided to carry out the calculations using three different functionals: wB97XD[S16], PBE0[S17] and B3LYP[S18]. All three functionals cover fairly distinct approximations and include explicitly or not effects such as long-range and dispersion corrections. As such and considering our results were consistent thorough, we are confident about the presented results. In all cases, the absence of imaginary frequencies (for ground-state calculations) or the presence of a unique imaginary frequency (for transition state calculations) was confirmed. In all cases, a conservative 6-31G(d,p) was used. The main reason for using this basis set is the relatively high number of atoms on the supramolecular tetramer calculations (232). Semi-empirical calculations use the PM6 method[S19] as implemented in Gaussian.

The calculation of the stacks formed by **2H-Car-H** using the B3LYP functional without dispersion correction resulted in the disassembly of the stack. When using the D3 dispersion correction by Grimme[S20], an equivalent structure to the other functionals was obtained with a comparable energy difference. Such behavior is expected considering the critical role of dispersion in these systems.

The activation energy barrier for the isomerization of the carpyridine core was calculated on the **2H-Car-H** structure. A redundant coordinate scan was performed by stepwise changing the dihedral angle of the carbazole-pyridine bond. The scan gave increasing and unhindered energy changes (Supplementary Table 5) until the most stable conformation became another local minimum (Supplementary Figure 56). Based on this scan, the corresponding transition state was calculated starting from a few of the high energy conformations using the Berny approximation. The transition state conformation could be found in this way. To estimate the energy barrier, the energies from the thermochemistry calculation (including Gibbs free energy correcting) where used for both the ground and the transition states. To confirm that the isomerization of the carpyridine core is a stepwise isomerization of the pyridine units and not a concerted flip, a 2D scan involving both pyridine units was carried out: A concerted flipping would require significantly more energy and, thus, we report here the energy of the transition to an intermediate conformation as the activation energy for the isomerization of the carpyridine core.

Supplementary Table 5. Differences in energy relative to the global ground state by changing the dihedral angle between the carbazole and pyridine planes in **2H-Car-H**. Calculated by DFT wB97XD/6-31G(d,p).

Dihedral angle / °	Relative energy / kJ·mol^{-1}	Dihedral angle / °	Relative energy / kJ·mol^{-1}
–44.70	0.00	–2.70	28.92
–42.70	0.08	–0.70	31.53
–40.70	0.30	1.30	34.17
–38.70	0.68	3.30	36.82
–36.70	1.19	5.30	39.42
–34.70	1.85	7.30	41.90
–32.70	2.65	9.30	44.21
–30.70	3.61	11.30	46.32
–28.70	4.70	13.30	48.24
–26.70	5.93	15.30	50.00
–24.70	6.50	17.30	51.67
–22.70	7.93	19.30	53.26
–20.70	9.49	21.30	54.78
–18.70	11.18	23.30	56.23
–16.70	13.00	25.30	57.63
–14.70	14.95	27.30	59.03
–12.70	17.02	29.30	60.41
–10.70	19.20	31.30	61.74
–8.70	21.50	33.30	63.03
–6.70	23.89	35.30	64.26
–4.70	26.37		

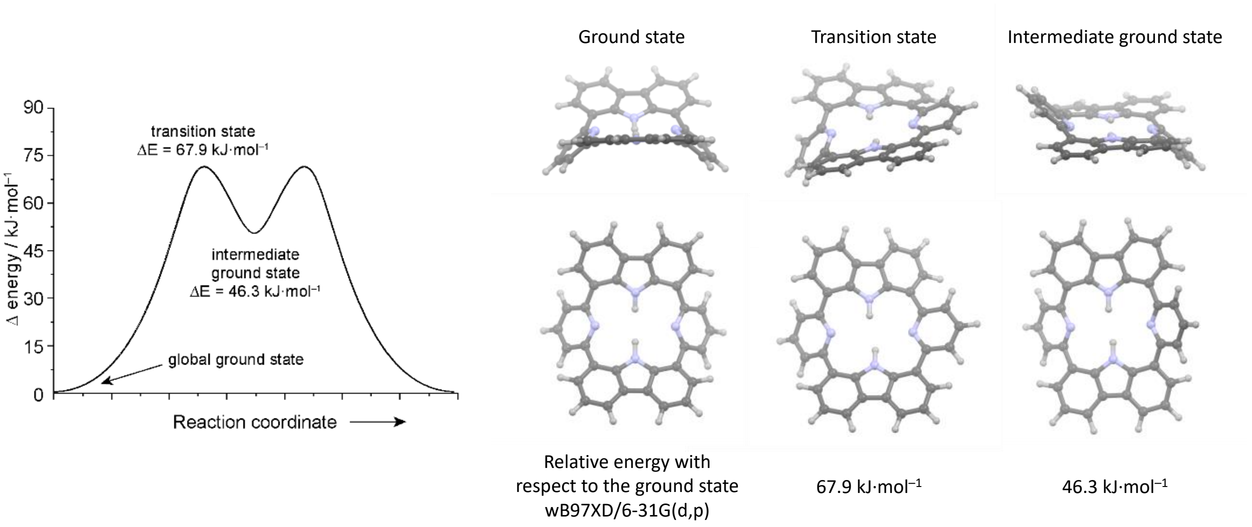

Supplementary Figure 56. Summary of the isomerization barrier for carpyridine inversion. **Left**, Potential energy surface corresponding to the isomerization process. The reaction coordinate corresponds (indirectly) to the dihedral angle formed between the pyridine and carbazole planes. **Right**, Optimized structures for the ground and transition states corresponding to the figure on the left.

Supplementary Table 6. Relative energy between the possible stack conformations for all three functionals (6-31G(d,p) as basis set). In all cases, the alternated conformation is more stable.

Relative energies<br>4 × **2H-Car-H** stack	wB97XD / kJ·mol^{-1}	PBE0 / kJ·mol^{-1}	B3LYP-D3 / kJ·mol^{-1}
Alternated	0.00	0.00	0.00
Slipped plane	2.61	3.33	3.89

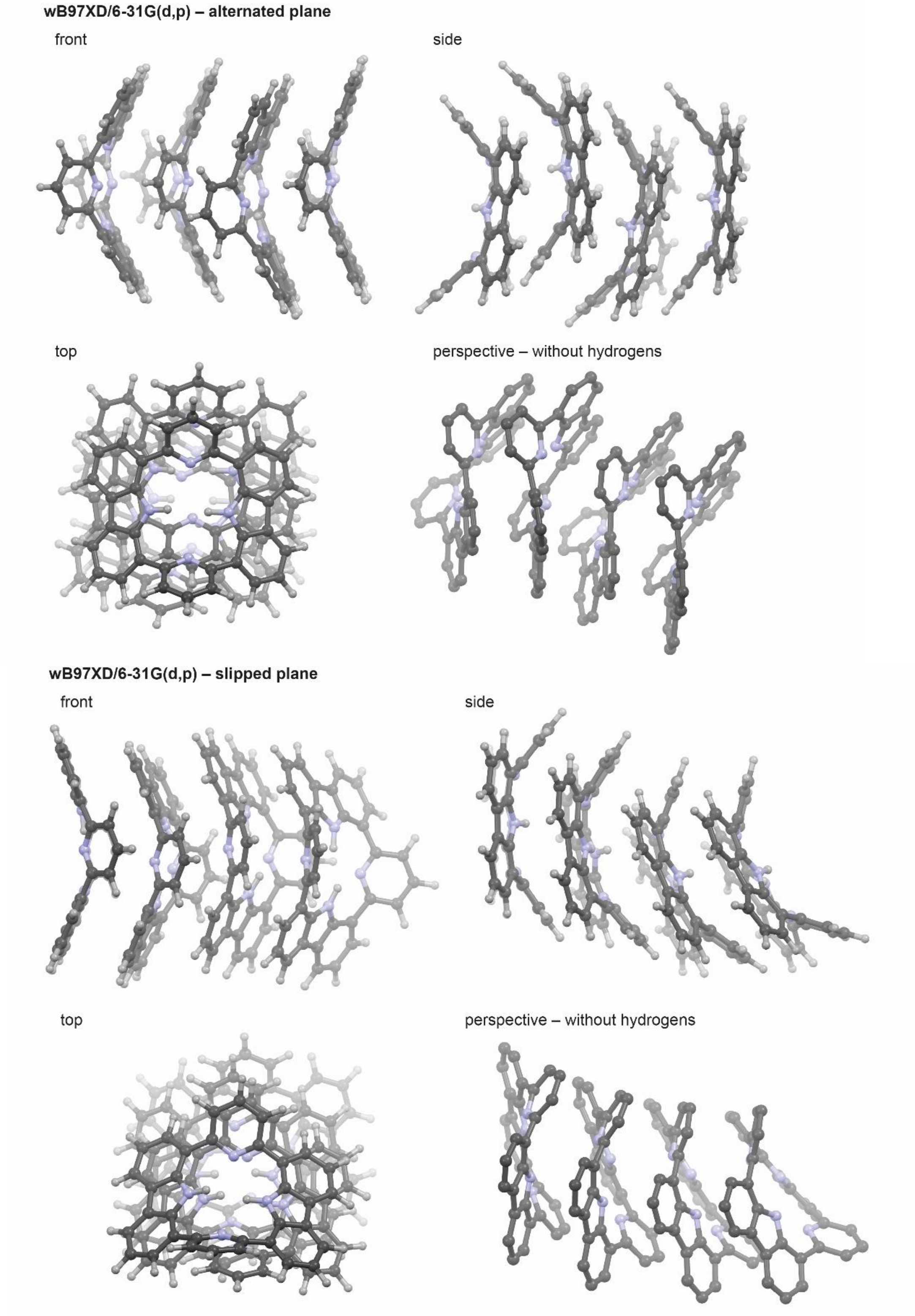

Supplementary Figure 57. Optimized structure of a stack of four **2H-Car-H** by DFT using wB97XD/6-31G(d,p). Both alternated (**top**) and slipped plane (**bottom**) are shown.

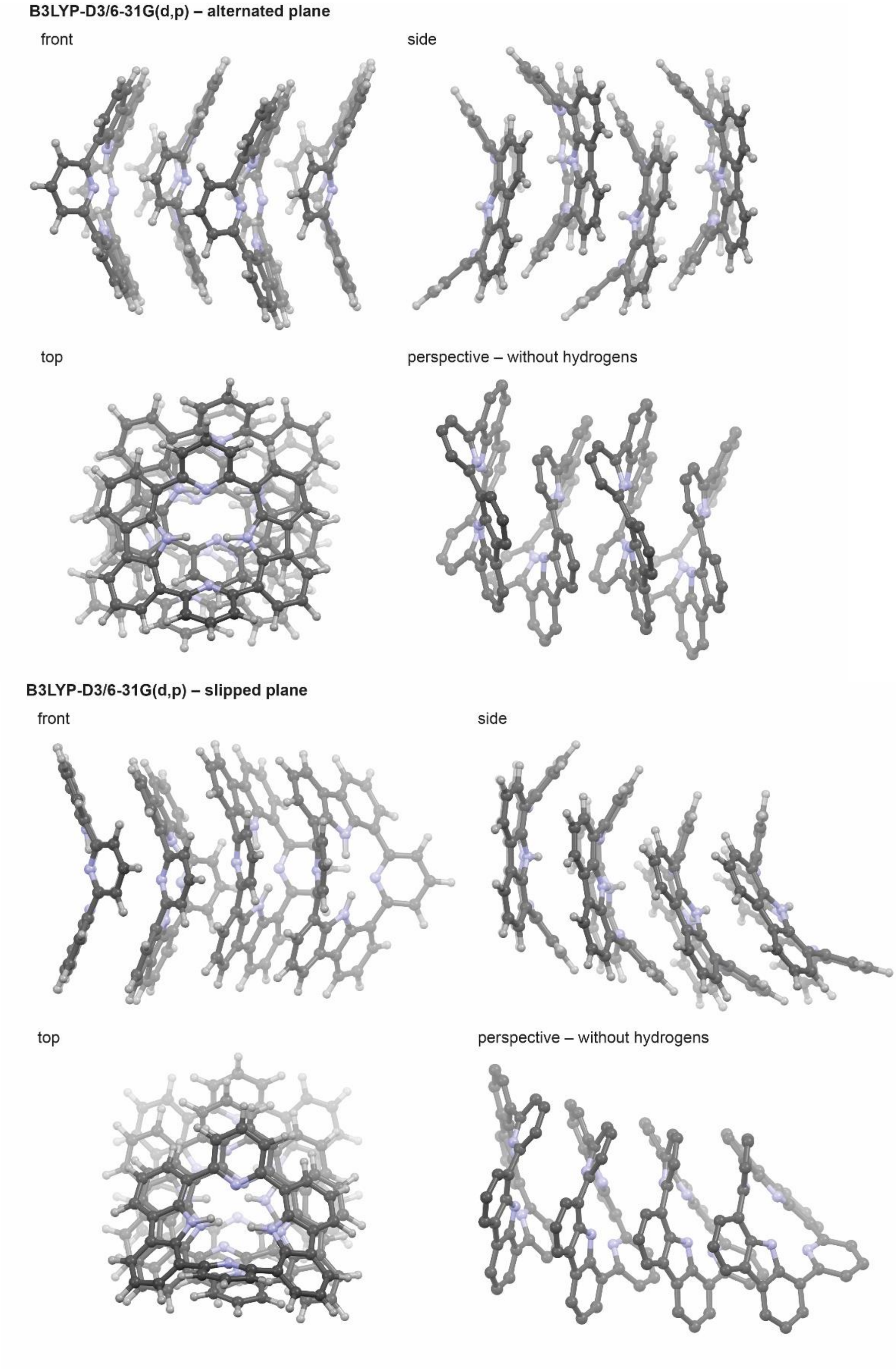

Supplementary Figure 58. Optimized structure of a stack of four **2H-Car-H** by DFT using B3LYP-D3/6-31G(d,p). Both alternated (**top**) and slipped plane (**bottom**) are shown.

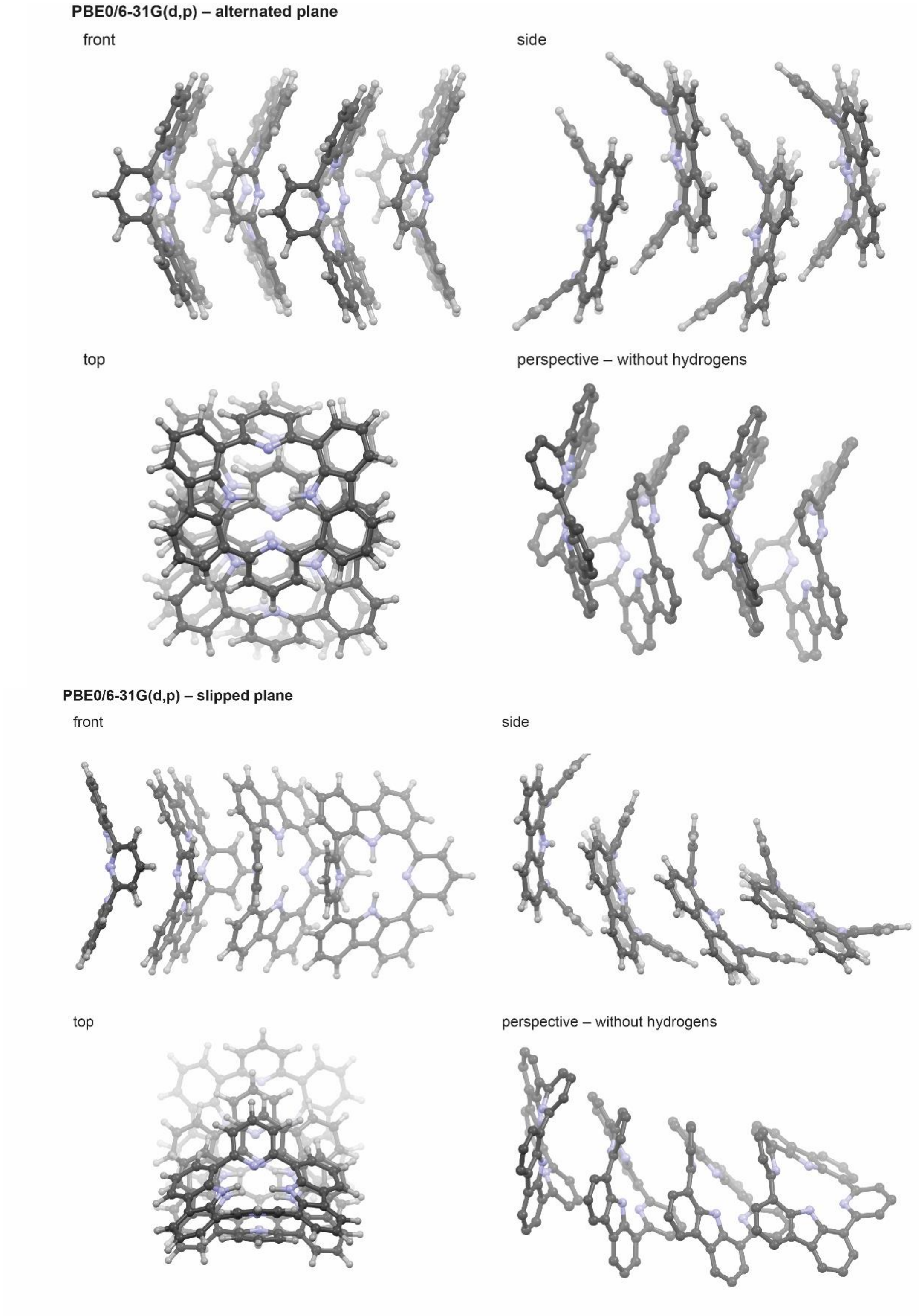

Supplementary Figure 59. Optimized structure of a stack of four **2H-Car-H** by DFT using PBE0/6-31G(d,p). Both alternated (**top**) and slipped plane (**bottom**) are shown.

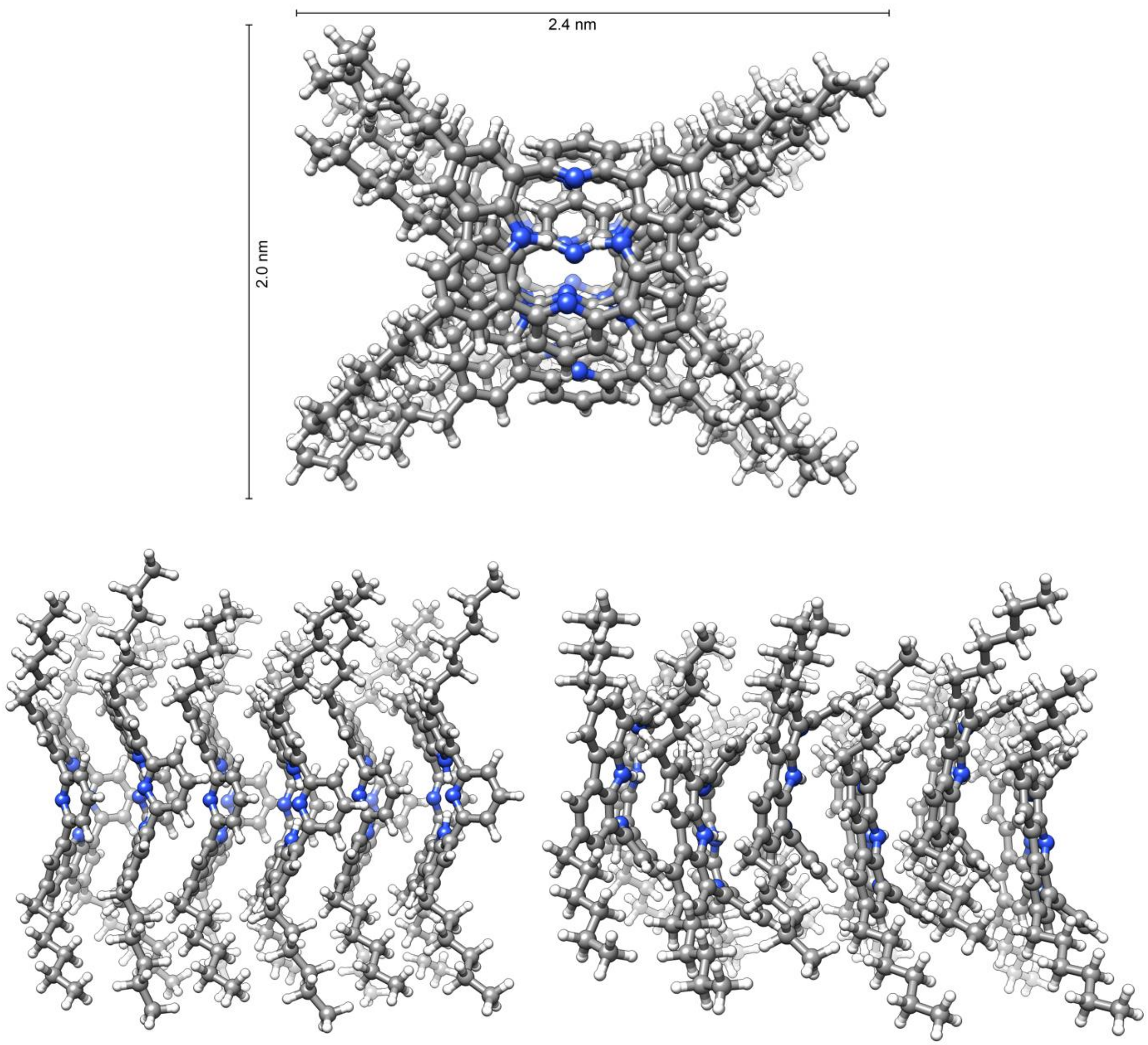

Supplementary Figure 60. Optimized structure of a stack of six **2H-Car-C6** using the semi-empirical PM6 method. **Top**, Top view. **Bottom**, Side views of the optimized stack. Slipped stacks did not yield stable solutions and resulted in collapsed or separated ensembles.

X-ray Diffraction

Methods

Definition of terms.

Function minimized: $\Sigma w(F_o^2 - F_c^2)^2$

where $w = [\sigma^2(F_o^2) + (aP)^2 + bP]^{-1}$ and $P = (F_o^2 + 2F_c^2)/3$

$F_o^2 = S(C - RB) / Lp$

and $\sigma^2(F_o^2) = S^2(C + R^2B) / Lp^2$

S = Scan rate

C = Total integrated peak count

R = Ratio of scan time to background counting time

B = Total background count

Lp = Lorentz-polarization factor

R-factors: $R_{int} = \Sigma|\langle F_o^2\rangle - F_o^2| / \Sigma F_o^2$ summed only over reflections for which more than one symmetry equivalent was measured.

$R(F) = \Sigma||F_o| - |F_c|| / \Sigma|F_o|$ summed over all observed reflections.

$wR(F^2) = [\Sigma w(F_o^2 - F_c^2)^2 / \Sigma w(F_o^2)^2]^{1/2}$ summed over all reflections.

Standard deviation of an observation of unit weight (goodness of fit):

$[\Sigma w(F_o^2 - F_c^2)^2 / (N_o - N_v)]^{1/2}$

where N_o = number of observations; N_v = number of variables

Crystal-Structure Determination. – A crystal of $C_{58}H_{68}N_4$, obtained from toluene and MeOH, was mounted on a cryo-loop and used for a low-temperature X-ray structure determination. All measurements were made on a *Rigaku Oxford Diffraction XtaLAB Synergy* diffractometer (*1*) with a *Pilatus 200K* hybrid pixel area detector using Cu *Kα* radiation (λ = 1.54184 Å) from a *PhotonJet* micro-focus X-ray source and an *Oxford Cryosystems Cryostream 800* cooler. The unit cell constants and an orientation matrix for data collection were obtained from a least-squares refinement of the setting angles of 27011 reflections in the range 5° < 2θ < 157°. A total of 3658 frames were collected using ω scans with κ offsets, 4.8-19.0 seconds exposure time and a rotation angle of 0.5° per frame, and a crystal-detector distance of 40.0 mm.

Data reduction was performed with *CrysAlisPro*[S21]. The intensities were corrected for Lorentz and polarization effects, and a numerical absorption correction[S22] was applied. The space group was uniquely determined by the systematic absences. Equivalent reflections were merged. The data collection and refinement parameters are given in *Supplementary Table 7*. A view of the molecule is shown in *Supplementary Figure 61*.

The structure was solved by dual space methods using *SHELXT-2018*[S23], which revealed the positions of all non-hydrogen atoms. Two XX groups are disordered over two conformations. Two sets of positions were defined for the atoms of each disordered XX group and the site occupation factors of the major conformations of these groups refined to 0.647(7) and 0.789(6), respectively. Two sets of positions were defined for the atoms of the XX group and the site occupation factor of the major conformation of the group refined to 0.572(4). Similarity restraints were applied to the chemically equivalent bond lengths and angles involving all disordered C-atoms, as well as to

the F···F distances, while neighboring atoms within and between each of the disordered conformations were restrained to have similar atomic displacement parameters. The non-hydrogen atoms were refined anisotropically. All of the H-atoms were placed in geometrically calculated positions and refined by using a riding model where each H-atom was assigned a fixed isotropic displacement parameter with a value equal to 1.2U_{eq} of its parent atom (1.5Ueq for the methyl groups). The refinement of the structure was carried out on F^2 by using full-matrix least-squares procedures, which minimized the function $\Sigma w(F_o^2 - F_c^2)^2$. The weighting scheme was based on counting statistics and included a factor to downweight the intense reflections. Plots of $\Sigma w(F_o^2 - F_c^2)^2$ versus $F_c/F_{c(max)}$ and resolution showed no unusual trends. A correction for secondary extinction was not applied.

Neutral atom scattering factors for non-hydrogen atoms were taken from Maslen, Fox and O'Keefe[S24], and the scattering factors for H-atoms were taken from Stewart, Davidson and Simpson[S25]. Anomalous dispersion effects were included in F_c[S26]; the values for f' and f'' were those of Creagh and McAuley[S27]. The values of the mass attenuation coefficients are those of Creagh and Hubbel[S28]. The *SHELXL-2018* program[S29] was used for all calculations.

2H-Car-C6

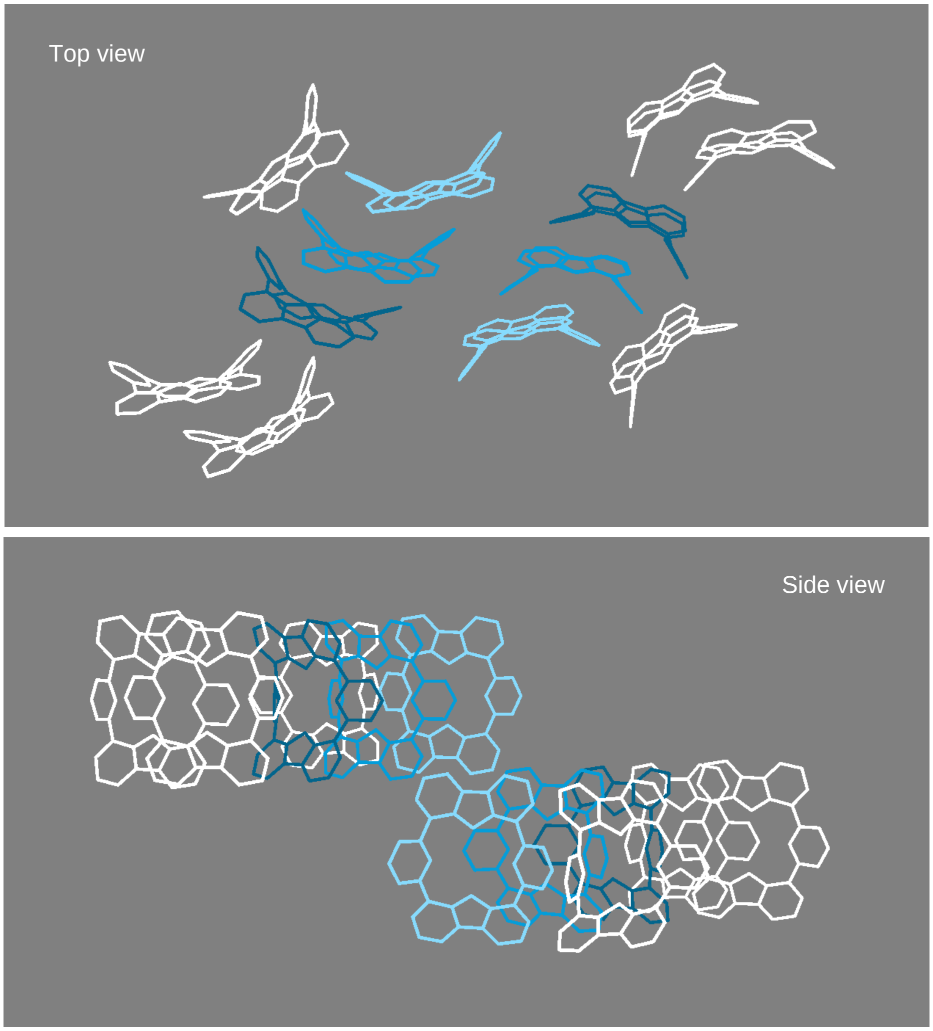

Supplementary Figure 61. X-ray structure of a crystal of **2H-Car-C6** grown from slow diffusion of methanol into toluene. Side chains and hydrogens are omitted for clarity. Blue colored molecules represent the repeating unit of packing along the crystal, with 3 carpyridines stacked and slipped on each other. The direction of the principal axis of curvature is retained across all carpyridines. The crystal was of very poor quality with high R-factor due to heavily disordered side chains.

Note: There are three symmetry-independent molecules in the asymmetric unit. Although the basic structure, connectivity and conformation of the core of the molecules is visible, they should be considered very approximate. The R-factors remain high and other quality indicators indicate very low precision and accuracy. The standard uncertainties on bond lengths between core atoms is in the range 0.008-0.012 Å. The main factors leading to these issues are overall poor crystal quality, strong streaks of diffuse scattering (a consequence of disorder), which mean that the integrated intensities are very inaccurate, and severe disorder of the hexyl sidechains, which is difficult to model adequately. Attempts to model some of the side-chain disorder were unsuccessful, because it is apparent that there might not be just two conformations of a disordered hexyl group, but multiple positions for the atoms; or this appearance might also be related to the inaccurate

intensities. Therefore, just the average positions for the atoms of some of the side chains were used. Consequently, the model has are a few chemically unreasonable short contacts between molecules, so the conformations of the side chains should be understood to be very approximate. Similarity restraints applied to the anisotropic displacement parameters of all neighboring C-atoms. Within the hexyl sidechains, all C–C bonds and 1,3 distances were restrained to be similar.

Supplementary Table 7. Crystallographic data

Crystallized from		toluene
Empirical formula		$C_{58}H_{68}N_4$
Formula weight / g mol^{-1}		821.16
Crystal color, habit		colorless, plate
Crystal dimensions / mm		0.04 × 0.13 × 0.34
Temperature / K		160(1)
Crystal system		monoclinic
Space group		$P2_1/c$ (#14)
Z		12
Reflections for cell determination		27011
2θ range for cell determination / °		5 – 157
Unit cell parameters	a / Å	20.1809(4)
	b / Å	17.2408(2)
	c / Å	42.4043(7)
	α / °	90
	β / °	101.9163(18)
	γ / °	90
	V / Å^3	14436.0(4)
$F(000)$		5328
D_x / g cm^{-3}		1.133
μ(Cu $K\alpha$) / mm^{-1}		0.495
Scan type		ω
$2\theta_{(max)}$ / °		133.2
Transmission factors (min; max)		0.525; 1.000
Total reflections measured		139382
Symmetry independent reflections		25504
R_{int}		0.069
Reflections with $I > 2\sigma(I)$		19750
Reflections used in refinement		25504
Parameters refined; restraints		1715; 1803
Final	$R(F)$ [$I > 2\sigma(I)$ reflections]	0.1928
	$wR(F^2)$ (all data)	0.5626
Weights:		$w = [\sigma^2(F_o^2) + (0.2000P)^2]^{-1}$ where $P = (F_o^2 + 2F_c^2)/3$
Goodness of fit		2.479
Final Δ_{max}/σ		0.003
$\Delta\rho$ (max; min) / e Å^{-3}		1.45; -0.97
$\sigma(d_{(C-C)})$ / Å	0.00 – 0.00	

Supplementary Table 8. Comparison of bond lengths, saddle depths and torsion angles between X-Ray structures and DFT predicted structures.

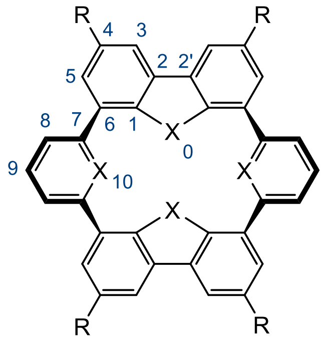

**Bond length / Å**	**2H-Car-*t*Bu X-Ray**[1] X = NH, R = *t*-Bu	**2H-Car-C6 X-Ray*** X = NH, R = C_6H_{13}	**2H-Car-C6 DFT** X = NH, R = C_6H_{13}	**4H-Car-C6 DFT** X = CH_2, R = C_6H_{13}
**X(0)–H**	1.00	0.88	1.01	1.09
**X(0)–C(1)**	1.39	1.40	1.39	1.51
**C(1)–C(2)**	1.44	1.40	1.42	1.41
**C(2)–C(2′)**	1.44	1.47	1.45	1.47
**C(2)–C(3)**	1.38	1.37	1.40	1.40
**C(3)–C(4)**	1.39	1.39	1.40	1.40
**C(4)–C(5)**	1.40	1.38	1.41	1.40
**C(5)–C(6)**	1.41	1.41	1.40	1.41
**C(1)–C(6)**	1.38	1.39	1.41	1.40
**C(6)–C(7)**	1.50	1.48	1.49	1.49
**C(7)–C(8)**	1.37	1.42	1.41	1.40
**C(8)–C(9)**	1.38	1.39	1.39	1.39
**C(7)–X(10)**	1.35	1.33	1.35	1.40
**Saddle depths / °**				
α_{cbz}	23.95	20.77	19.08	19.14
α_{py}	42.18	37.50	37.70	46.16
**Torsion angles / °**				
$P_{cbz\text{-}py}$	49.37	40.83	43.26	49.13

[1] Arnold L., Norouzi-Arasi H., Wagner M., Enkelmann V., Müllen K. A porphyrin-related macrocycle from carbazole and pyridine building blocks: synthesis and metal coordination. *Chem. Commun.* **47**, 970–972 (2011).
*crystal of very poor quality with high R-factor due to heavily disordered side chains.

References and Notes

S1. Insua, I. & Montenegro, J. 1D to 2D self assembly of cyclic peptides. *J. Am. Chem. Soc*. **142**, 300–307 (2020).

S2. Wang, Z., Li, Z., Medforth, C. J. & Shelnutt, J. A. Self-assembly and self-metallization of porphyrin nanosheets. *J. Am. Chem. Soc*. **129**, 2440–2441 (2007).

S3. Lewandowska, U. et al. A triaxial supramolecular weave. *Nat. Chem*. **9**, 1068–1072 (2017).

S4. Li, Y. et al. A bio-inspired molecular design strategy toward 2D organic semiconductor crystals with superior integrated optoelectronic properties. *Small* **17**, 2102060 (2021).

S5. Lin, Y. et al. Self-assembled 2D free-standing Janus nanosheets with single-layer thickness. *J. Am. Chem. Soc*. **139**, 13592–13595 (2017).

S6. He, C. et al. Two-dimensional nanostructures via the assembly of n-type tetraazaanthracene-based conjugated molecules. *ChemPhysChem* **14**, 2954–2960 (2013).

S7. Wang, Y., Kim, Y. & Lee, M. Static and dynamic nanosheets from selective assembly of geometric macrocycle isomers. *Angew. Chem. Int. Ed*. **55**, 13122–13126 (2016).

S8. Wang, X.-Y. et al. Postfunctionalization of BN-embedded polycyclic aromatic compounds for fine-tuning of their molecular properties. *Chem. Eur. J.* **21**, 8867–8873 (2015).

S9. Yasuda, T., Shimizu, T., Liu, F., Ungar, G. & Kato, T. Electro-functional octupolar π-conjugated columnar liquid crystals. *J. Am. Chem. Soc.* **133**, 13437–13444 (2011).

S10. Liu, Y., Nishiura, M., Wang, Y. & Hou, Z. π-conjugated aromatic enynes as a single-emitting component for white electroluminescence. *J. Am. Chem. Soc.* **128**, 5592–5593 (2006).

S11. Bennington, M. S., Feltham, H. L. C., Buxton, Z. J., White, N. G. & Brooker, S. Tuneable reversible redox of cobalt(III) carbazole complexes. *Dalton Trans.* **46**, 4696–4710 (2017).

S12. Malthus, S. J., Cameron, S. A. & Brooker, S. Improved access to 1,8-diformyl-carbazoles leads to metal-free carbazole-based [2 + 2] Schiff base macrocycles with strong turn-on fluorescence sensing of zinc(II) ions. *Inorg. Chem.* **57**, 2480–2488 (2018).

S13. Arnold, L., Norouzi-Arasi, H., Wagner, M., Enkelmann, V. & Müllen, K. A porphyrin-related macrocycle from carbazole and pyridine building blocks: synthesis and metal coordination. *Chem. Commun.* **47**, 970–972 (2011).

S14. Liyu, J. & Sperry, J. Synthesis of putative clausenal from carbazole using sequential C–H borylations. *Tetrahedron Lett*. **58**, 1699–1701 (2017).

S15. Gaussian 16, Revision C.01, Frisch, M. J. et al. Gaussian, Inc., Wallingford CT, 2016.

S16. Chai, J.-D. & Head-Gordon, M. Long-range corrected hybrid density functionals with damped atom–atom dispersion corrections. *Phys. Chem. Chem. Phys.* **10**, 6615 (2008).

S17. Adamo, C. & Barone, V. Toward reliable density functional methods without adjustable parameters: the PBE0 model. *J. Chem. Phys*. **110**, 6158–6170 (1999).

S18. Becke, A. D. Density-functional thermochemistry. III. The role of exact exchange. *J. Chem. Phys*. **98**, 5648–5652 (1993).

S19. Stewart, J. J. P. Optimization of parameters for semiempirical methods V: modification of NDDO approximations and application to 70 elements. *J. Mol. Model*. **13**, 1173–1213 (2007).

S20. Grimme, S., Antony, J., Ehrlich, S. & Krieg, H. A consistent and accurate ab initio parametrization of density functional dispersion correction (DFT-D) for the 94 elements H-Pu. *J. Chem. Phys*. **132**, 154104 (2010).

S21. Rigaku Oxford Diffraction, CrysAlisPro Software System, Version 1.171.41.116a, Rigaku Corporation, Wroclaw, Poland (2021).

S22. Coppens, P., Leiserowitz, L., Rabinovich, D. Calculation of absorption corrections for camera and diffractometer data. *Acta Crystallogr*. **18**, 1035–1038 (1965).
S23. Sheldrick, G. M. SHELXT – Integrated space-group and crystal-structure determination. *Acta Crystallogr. A: Found. Adv*. **71**, 3–8 (2015).
S24. Maslen, E. N., Fox, A. G., O'Keefe, M. A. in "International Tables for Crystallography", Ed. A. J. C. Wilson, Kluwer Acade.
S25. Stewart, R. F., Davidson, E. R., Simpson, W. T. *J. Chem. Phys*. **42**, 3175–3187 (1965).
S26. Ibers, J. A., Hamilton, W. C. *Acta Crystallogr.* **17**, 781–782 (1964).
S27. Creagh, D. C., McAuley, W. J. in "International Tables for Crystallography", Ed. A. J. C. Wilson, Kluwer Academic Publish.
S28. Creagh, D. C., Hubbell, J. H. in "International Tables for Crystallography", Ed. A. J. C. Wilson, Kluwer Academic Publish.
S29. Sheldrick, G. M. Crystal structure refinement with SHELXL. *Acta Crystallogr. C: Struct. Chem*. **71**, 3–8 (2015).
